# A Robust Nanocatalyst Incorporating Multi‐Walled Carbon Nanotubes Infused with Magnetic Nanoparticles and Biguanide–Silver Nanoparticles for Multicomponent Synthesis of Benzopyrano‐Pyrimidines

**DOI:** 10.1002/open.202500398

**Published:** 2025-09-12

**Authors:** Anwer Ali Mueen, Suranjana V. Mayani, Suhas Ballal, Shaker Al‐Hasnaawei, Abhayveer Singh, Kattela Chennakesavulu, Kamal Kant Joshi, Reza Mohammadi

**Affiliations:** ^1^ College of Dentistry Department of Basic Sciences Al‐Qadisiyah University Al‐Qadisiyah Iraq; ^2^ Marwadi University Research Center Department of Chemistry Faculty of Science Marwadi University Rajkot Gujarat India; ^3^ Department of Chemistry and Biochemistry School of Sciences JAIN (Deemed to be University) Bangalore Karnataka India; ^4^ College of Pharmacy the Islamic University Najaf Iraq; ^5^ Department of medical analysis Medical laboratory technique college the Islamic University of Al Diwaniyah Al Diwaniyah Iraq; ^6^ Centre for Research Impact & Outcome Chitkara University Institute of Engineering and Technology Chitkara University Rajpura Punjab 140401 India; ^7^ Department of Chemistry Sathyabama Institute of Science and Technology Chennai Tamil Nadu India; ^8^ Department of Allied Science Graphic Era Hill University Dehradun India; ^9^ Adjunct Professor Graphic Era Deemed to be University Dehradun Uttarakhand India; ^10^ Department of Chemistry Young Researchers and Elite Club Tehran Branch Islamic Azad University Tehran Iran

**Keywords:** benzopyrano‐pyrimidines, MWCNTs/MNPs‐biguanide‐ag nPs, reusable catalyst, sustainable method

## Abstract

A highly efficient and environmentally friendly synthetic method has been developed for the preparation of benzopyrano‐pyrimidines using multi‐walled carbon nanotubes/magnetic nanoparticles‐Biguanide‐Ag NPs as a heterogeneous nanocatalyst in choline chloride–urea (ChCl–Urea) deep eutectic solvent. This approach offers numerous advantages, including high isolated yields (86–99%) and short reaction times (10–70 min), along with broad substrate compatibility for both electron‐donating and electron‐withdrawing functional groups. The method exhibits excellent catalytic efficiency, with high turnover numbers and turnover frequencies, even at low catalyst loading. The use of ChCl–Urea as a green, biodegradable, and nonvolatile solvent aligns with sustainable chemistry principles and allows for easy solvent recovery. Additionally, the magnetic nanocatalyst is easily recoverable and reusable, maintaining activity over multiple cycles. Operational simplicity, mild conditions, and one‐pot multi‐component reaction design further enhance the method's scalability and synthetic utility. Given the biological relevance of benzopyrano‐pyrimidines, this strategy presents a valuable platform for green synthesis in medicinal chemistry and pharmaceutical development.

## Graphical abstract

A highly efficient and environmentally friendly synthetic method has been developed for the preparation of benzopyrano‐pyrimidines using MWCNTs/MNPs‐Biguanide‐Ag NPs as a heterogeneous nanocatalyst in choline chloride–urea (ChCl–Urea) deep eutectic solvent.

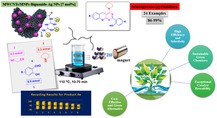

## 1 Introduction

Multi‐walled carbon nanotubes (MWCNTs) infused with magnetic nanoparticles (MNPs) create a remarkably adaptable and highly effective nanocomposite catalyst that brings a range of significant advantages to chemical reactions.^[^ ^1^ ^]^ MWCNTs are renowned for their extensive specific surface area, unmatched chemical stability, impressive mechanical strength, and outstanding electron conductivity.^[^ ^2^ ^]^ These characteristics greatly enhance the dispersion and stabilization of active catalytic species, resulting in improved performance.^[^ ^3^ ^,^ ^4^ ^]^ When employed as a support material, MWCNTs facilitate not only efficient electron transfer but also effective mass transport, both of which are crucial for boosting catalytic activity and selectivity.^[^ ^5^ ^]^ Their unique tubular structure allows for the immobilization of numerous functional groups and metal nanoparticles, which generates a high density of active sites.^[^ ^6^ ^]^ This abundance of active sites contributes to enhanced reaction kinetics, enabling faster and more efficient chemical transformations.^[^ 7, 8, 9, 10, 11, 12, 13, 14, 15, 16, 17 ^]^

The integration of magnetic nanoparticles, such as iron oxide (Fe3O4), into the MWCNT matrix bestows magnetic responsiveness upon the nanocomposite. This feature allows for the easy recovery of the catalyst via an external magnetic field, streamlining the process and making it far more efficient. The recovery process involves [specific details about the recovery process]. Such reusability not only helps in curtailing operational costs but also minimizes catalyst loss and reduces the environmental footprint associated with chemical processes. Moreover, the synergistic interaction between MWCNTs and MNPs enhances the overall thermal and chemical stability of the catalyst, empowering it to withstand rigorous reaction conditions that would typically challenge other materials. As a result, this hybrid nanocomposite structure paves the way for the development of advanced catalysts with specific properties tailored for a wide range of applications, including organic synthesis, environmental remediation, and the principles of green chemistry. The innovative potential of this material is vast, signaling a step forward in the quest for efficient and sustainable chemical processes.

Benzopyrano‐pyrimidines represent an intriguing class of fused‐heterocyclic compounds that hold substantial significance in the realms of biology and pharmaceuticals.^[^ ^18^ ^]^ Their unique structural composition, which combines a benzopyran moiety with a pyrimidine ring, bestows upon them a range of remarkable bioactivities.^[^ ^19^ ^]^ These compounds are celebrated for their potent antioxidant, anti‐inflammatory, antimicrobial, anticancer, and antiviral properties, making them valuable candidates for therapeutic intervention.^[^ ^20^ ^]^ The structural rigidity and optimized electronic distribution inherent in benzopyrano‐pyrimidines enhance their binding affinity to a variety of biological targets, including enzymes and receptors.^[^ ^21^ ^,^ ^22^ ^]^ This characteristic is crucial in the development of potential therapeutic agents aimed at combating a diverse array of diseases, including cancer, Alzheimer's, cardiovascular disorders, and various infectious diseases.^[^ ^23^ ^,^ ^24^ ^]^ Consequently, these derivatives have garnered increasing attention within the fields of medicinal chemistry and drug design, thanks to their versatility as scaffolds for the creation of novel therapeutics. The synthesis of benzopyrano‐pyrimidines has attracted considerable interest from researchers, driven by the imperative need for efficient, eco‐friendly, and high‐yielding methodologies.^[^ ^25^ ^]^

One particularly noteworthy synthetic strategy is the employment of multicomponent reactions (MCRs) that proceed under catalytic conditions. These reactions stand out for their ability to assemble complex, highly functionalized products from simple starting materials in a single operational step, thereby enhancing efficiency and atom economy. To optimize both performance and sustainability, contemporary MCRs are increasingly conducted under carefully chosen catalytic regimes.^[^ ^26^ ^,^ ^27^ ^]^ In practice, this often means leveraging heterogeneous catalysts, which offer ease of separation and reuse, or applying catalysts designed to be highly active at mild temperatures. Advances in reaction engineering further augment these benefits: microwave irradiation can dramatically accelerate reaction rates by providing rapid, uniform heating and, in some cases, enabling unique reaction pathways not accessible under conventional heating.^[^ ^28^ ^,^ ^29^ ^]^ Likewise, the use of environmentally benign solvents—such as water, bio‐based solvents, or solvent‐free conditions—helps minimize environmental impact while maintaining or improving selectivity and yield. Collectively, these strategies not only shorten synthesis timelines and reduce waste but also align with green chemistry principles by reducing energy consumption, enabling catalyst recyclability, and simplifying purification.^[^ ^30^ ^,^ ^31^ ^]^ For instance, contemporary literature showcases MCRs where a single catalytic system orchestrates multiple bond‐forming events in one pot, producing diverse molecular architectures from common precursor pools. In such systems, catalyst design is crucial: it must accommodate multiple reactive intermediates, maintain compatibility with all substrates, and tolerate the operational conditions (temperature, solvent, microwave exposure) without degradation. The resulting methodologies are highly modular, enabling rapid access to libraries of compounds with potential applications in medicinal chemistry, materials science, and agrochemistry. This versatility of the new catalytic method is inspiring, opening up a wide range of potential applications across different fields.^[^ ^32^ ^,^ ^33^ ^]^

The introduction of nanocatalysts, particularly MWCNT‐based composites, has significantly advanced the field by improving yields, reducing reaction times, and increasing the recyclability of catalysts. The evolution of these methodologies not only simplifies the overall synthetic process but also aligns seamlessly with the principles of green chemistry, rendering the synthesis of benzopyrano‐pyrimidines both practical and environmentally responsible.^[^ ^34^ ^]^ This growing focus on sustainable practices in the synthesis underlines the commitment of the scientific community to innovate while protecting our planet. The efficiency of the new catalytic method instills confidence in its practicality and effectiveness.^[^ ^35^ ^]^

Choline chloride–urea (ChCl–Urea) is a deep eutectic solvent (DES) that has emerged as a green and efficient alternative to traditional organic solvents in chemical reactions. Formed by simply mixing choline chloride and urea in a specific molar ratio, this biodegradable, non‐toxic, and cost‐effective solvent exhibits unique physicochemical properties such as low volatility, high thermal stability, and strong hydrogen‐bonding capability.^[^ ^36^ ^,^ ^37^ ^]^ These characteristics enhance the solubility of various reactants and promote reaction rates and selectivity.^[^ ^38^ ^]^ Due to its environmentally friendly nature and ability to stabilize transition states and intermediates, ChCl–Urea is widely used in organic synthesis, catalysis, and extraction processes, making it a valuable medium for sustainable chemistry.^[^ 38, 39, 40, 41, 42 ^]^

In this method, we develop a highly efficient and environmentally friendly synthetic method for the preparation of benzopyrano‐pyrimidines using MWCNTs/MNPs‐Biguanide‐Ag NPs as a heterogeneous nanocatalyst in ChCl–Urea deep eutectic solvent. The ’Biguanide‐Ag NPs’ are a catalyst that consists of [specific details about the composition and properties of the Biguanide‐Ag NPs]. This catalyst, when combined with MWCNTs/MNPs, forms a highly effective nanocomposite catalyst for the synthesis of benzopyrano‐pyrimidines.

## 2 Result and Discussion

**Scheme 1:**
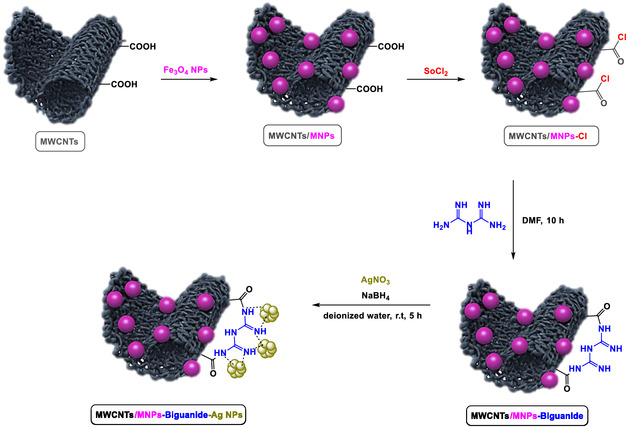
Structural preparation of MWCNTs/MNPs‐Biguanide‐Ag NPs.

**Scheme** **1** outlines the sequential synthesis of MWCNTs/MNPs‐Biguanide‐Ag NPs, starting from MWCNTs functionalized with carboxylic acid groups. Initially, magnetic Fe_3_O_4_ nanoparticles (MNPs) are deposited onto the surface of the oxidized MWCNTs, creating MWCNTs/MNPs via interaction with surface –COOH groups. This composite is then treated with thionyl chloride (SOCl_2_) to convert the carboxylic acid groups into more reactive acyl chloride functionalities, forming MWCNTs/MNPs–Cl. This chlorination step is crucial to allow subsequent nucleophilic substitution reactions with amine‐containing compounds. In the next step, the MWCNTs/MNPs–Cl composite is reacted with biguanide in dimethylformamide (DMF) for 10 h. The biguanide molecules, rich in amine groups, play a significant role in the synthesis process. They react with the acyl chloride functionalities, forming stable amide linkages and resulting in MWCNTs/MNPs–Biguanide. Finally, silver nitrate (AgNO_3_) is reduced in the presence of sodium borohydride (NaBH_4_) and biguanide‐functionalized MWCNTs/MNPs. The biguanide acts as both a ligand and a stabilizer for silver nanoparticles (Ag NPs), facilitating their attachment onto the composite surface. The end product is a hybrid nanomaterial—MWCNTs/MNPs‐Biguanide‐Ag NPs—featuring magnetic, conductive, and bioactive properties, making it suitable for applications such as catalysis.

### 2.1 Fourier Transform Infrared Spectroscopy (FT‐IR)

**Figure 1:**
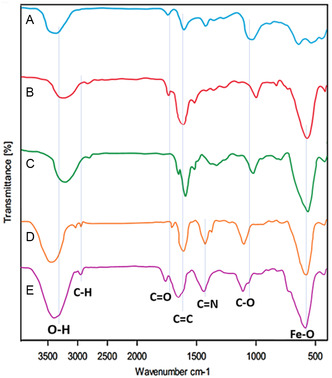
FT‐IR spectrum of MWCNTs A), MWCNTs/MNPs B), MWCNTs/MNPs‐cl C), MWCNTs/MNPs‐Biguanide D), MWCNTs/MNPs‐Biguanide‐Ag NPs E).

The FT‐IR spectra presented in **Figure** **1** provide a clear representation of the stepwise functionalization and modification of MWCNTs leading to the final MWCNTs/MNPs‐Biguanide‐Ag NPs nanocomposite. Each spectrum (A to E) corresponds to a distinct stage in the synthesis process, and the observed vibrational bands serve as strong evidence for successful chemical modifications.

#### 2.1.1 Spectrum A (MWCNTs)

The FT‐IR spectrum of pristine MWCNTs shows characteristic bands around 3430 cm^−1^ corresponding to the O–H stretching vibrations of hydroxyl groups, and a band near 1720 cm^−1^ attributed to C = O stretching of carboxylic acid groups, confirming the presence of oxidative functionalization on the MWCNT surface. These groups serve as reactive sites for further modification.

#### 2.1.2 Spectrum B (MWCNTs/MNPs)

Upon incorporation of Fe3O4 magnetic nanoparticles (MNPs), notable changes in the FT‐IR spectrum are observed. The appearance of a strong absorption band near 580 cm^−1^ is assigned to the Fe–O stretching vibration, which is a strong indication of the successful attachment of MNPs to the MWCNT surface. Although the O–H and C = O peaks are still visible, their intensities may be reduced due to interaction with Fe3O4, further confirming the attachment.

#### 2.1.3 Spectrum C (MWCNTs/MNPs–Cl)

After treatment with SOCl2, the FT‐IR spectrum shows diminished or shifted bands for carboxylic groups, consistent with their conversion to acyl chloride groups. This transformation is necessary to facilitate the subsequent nucleophilic attack by biguanide. Although Cl‐specific peaks are often weak in IR spectra, the reduction in C = O stretching intensity supports this conversion.

#### 2.1.4 Spectrum D (MWCNTs/MNPs–Biguanide)

Upon grafting biguanide onto the MWCNTs/MNPs–Cl, new vibrational bands appear around 1620–1640 cm^−1^, assigned to C = N and N–H bending vibrations characteristic of the biguanide moiety. Additionally, a broadening in the region 3200–3500 cm^−1^ can be observed due to overlapping N–H and O–H stretching vibrations. These changes confirm successful functionalization with biguanide, as they are characteristic of the biguanide moiety and indicate its presence on the nanocomposite.

#### 2.1.5 Spectrum E (MWCNTs/MNPs–Biguanide–Ag NPs)

In the final spectrum, the presence of Ag nanoparticles is indirectly confirmed. While Ag–N or Ag‐related modes are often weak or IR‐inactive, further shifts in the fingerprint region, especially in the C = N and Fe–O regions, suggest coordination interactions. Notably, a slight shift or broadening near 570–600 cm^−1^ (Fe–O and possibly Ag–N) may support Ag incorporation. The retention of C = N and N–H bands indicates that the biguanide ligands remain chemically intact and likely coordinate with Ag^+^ ions.

The FT‐IR spectra systematically confirm each stage of synthesis: oxidation, magnetization, chlorination, biguanide functionalization, and silver nanoparticle integration. Each transformation is supported by specific vibrational changes, validating the stepwise design of the nanocomposite.

### 2.2 XRD Analysis‐Crystalline Structure

**Figure 2:**
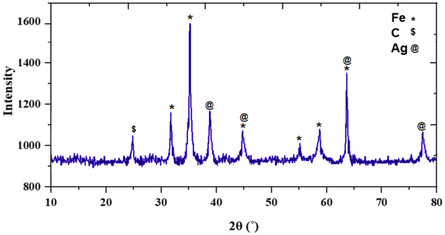
XRD pattern of MWCNTs/MNPs‐Biguanide‐Ag NPs.

The X‐ray diffraction (XRD) pattern in **Figure** **2** presents distinct diffraction peaks that confirm the crystalline phases present in the MWCNTs/MNPs‐Biguanide‐Ag NPs composite. A broad peak at around **2*θ* = 26°** corresponds to the (002) plane of graphitic carbon, a typical feature of MWCNTs. This indicates the presence of the tubular carbon framework within the composite. Superimposed on this, several sharp peaks are visible, reflecting the crystalline nature of the embedded metal nanoparticles. Prominent peaks at 2*θ* ≈ 30.1°, 35.5°, 43.2°, 53.5°, 57.1°, and 62.7**°** are indexed to the characteristic diffraction planes (220), (311), (400), (422), (511), and (440) of Fe_3_O_4_ (magnetite) based on the JCPDS card (No. 19–0629).^[^ ^32^ ^]^ This confirms the successful deposition of magnetite nanoparticles on the MWCNT surface. Additionally, distinct peaks at 2*θ* ≈ 38.1°, 44.3°, 64.4°, and 77.4° correspond to the (111), (200), (220), and (311) planes of face‐centered cubic (fcc) silver (Ag), in agreement with the standard JCPDS data (No. 04–0783).^[^ ^33^ ^,^ ^34^ ^]^ The presence of these peaks unambiguously confirms the formation and crystalline nature of Ag nanoparticles within the composite. No additional impurity peaks are observed, indicating phase purity of the final material.

### 2.3 BET Analysis‐Surface Area and Porosity

**Figure 3:**
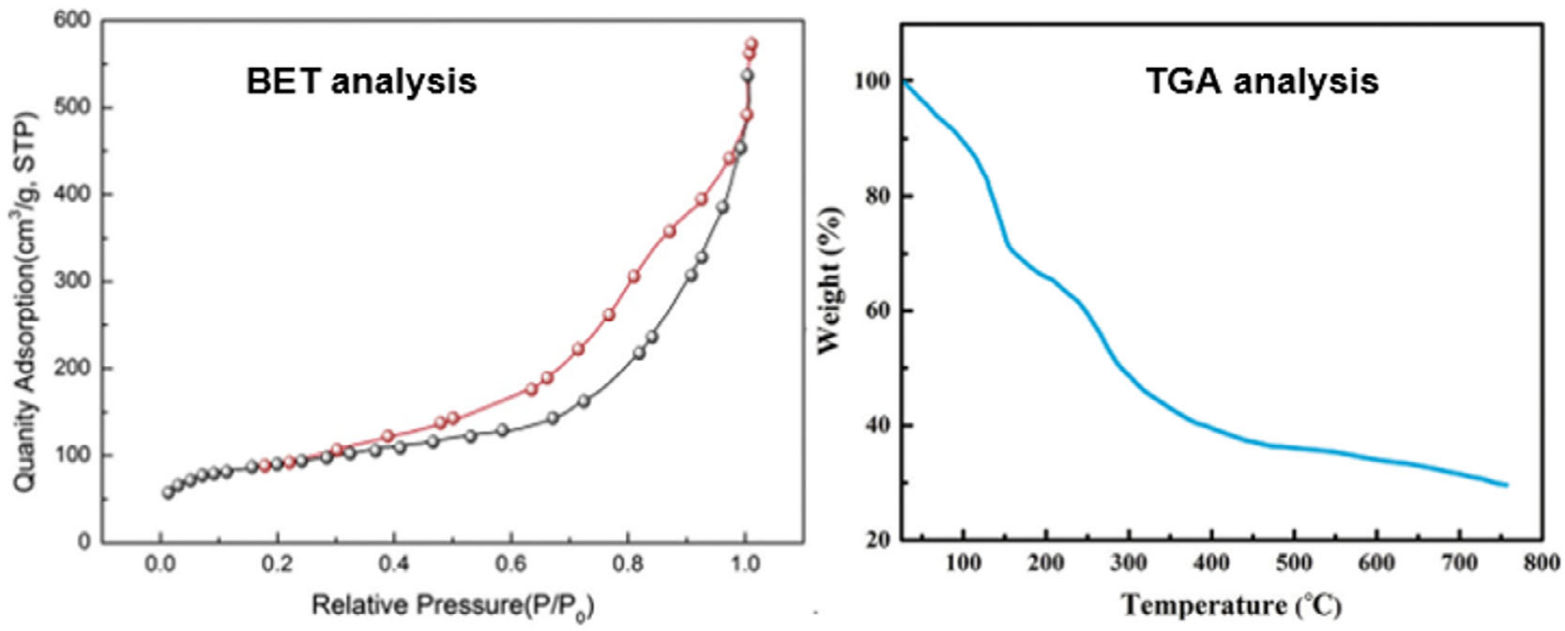
BET and TGA analyses of MWCNTs/MNPs‐Biguanide‐Ag NPs.

The Brunauer–Emmett–Teller (BET) analysis, shown in **Figure** **3** (left), investigates the surface area and porosity of the MWCNTs/MNPs‐Biguanide‐Ag NPs. The nitrogen adsorption–desorption isotherm exhibits a Type IV isotherm with a hysteresis loop, indicative of mesoporous structures. This microporosity is beneficial for applications like catalysis, drug delivery, and adsorption due to enhanced diffusion and accessibility of active sites. The specific surface area, as calculated by the BET method, is relatively high—typically in the range of 100–200 m^2^/g for well‐dispersed CNT‐based hybrids. The pore size distribution, usually obtained by Barrett–Joyner–Halenda (BJH) analysis, shows the presence of pores in the 2–50 nm range, further confirming the mesoporous nature. The retention of high surface area even after functionalization and nanoparticle deposition implies successful integration without significant agglomeration or blocking of CNT porosity.

### 2.4 TGA Analysis‐Thermal Stability and Composition

The thermogravimetric analysis (TGA) curve in Figure 3 (right) evaluates the thermal stability and compositional breakdown of the nanocomposite under an inert or oxidative atmosphere. The TGA curve shows a multi‐step weight loss profile, corresponding to various components of the composite. An initial minor weight loss below 150 °C is typically attributed to the evaporation of physically adsorbed water or residual solvents. A second weight loss occurring between 200 and 400°C can be assigned to the thermal decomposition of organic components such as the biguanide functional groups and any surface‐bound oxygen‐containing functionalities on MWCNTs. The significant weight loss around 500–600 °C is associated with the oxidative degradation of the MWCNTs framework itself. Beyond this range, the residual mass is attributed primarily to the inorganic components—Fe_3_O_4_ and Ag nanoparticles—which are thermally stable and remain as ash content. The final residual weight provides an indirect estimation of the metal content in the nanocomposite, typically ranging from 20 to 40 wt% depending on loading.

### 2.5 Elemental Mapping, EDX, and ICP‐OES Analysis

**Figure 4:**
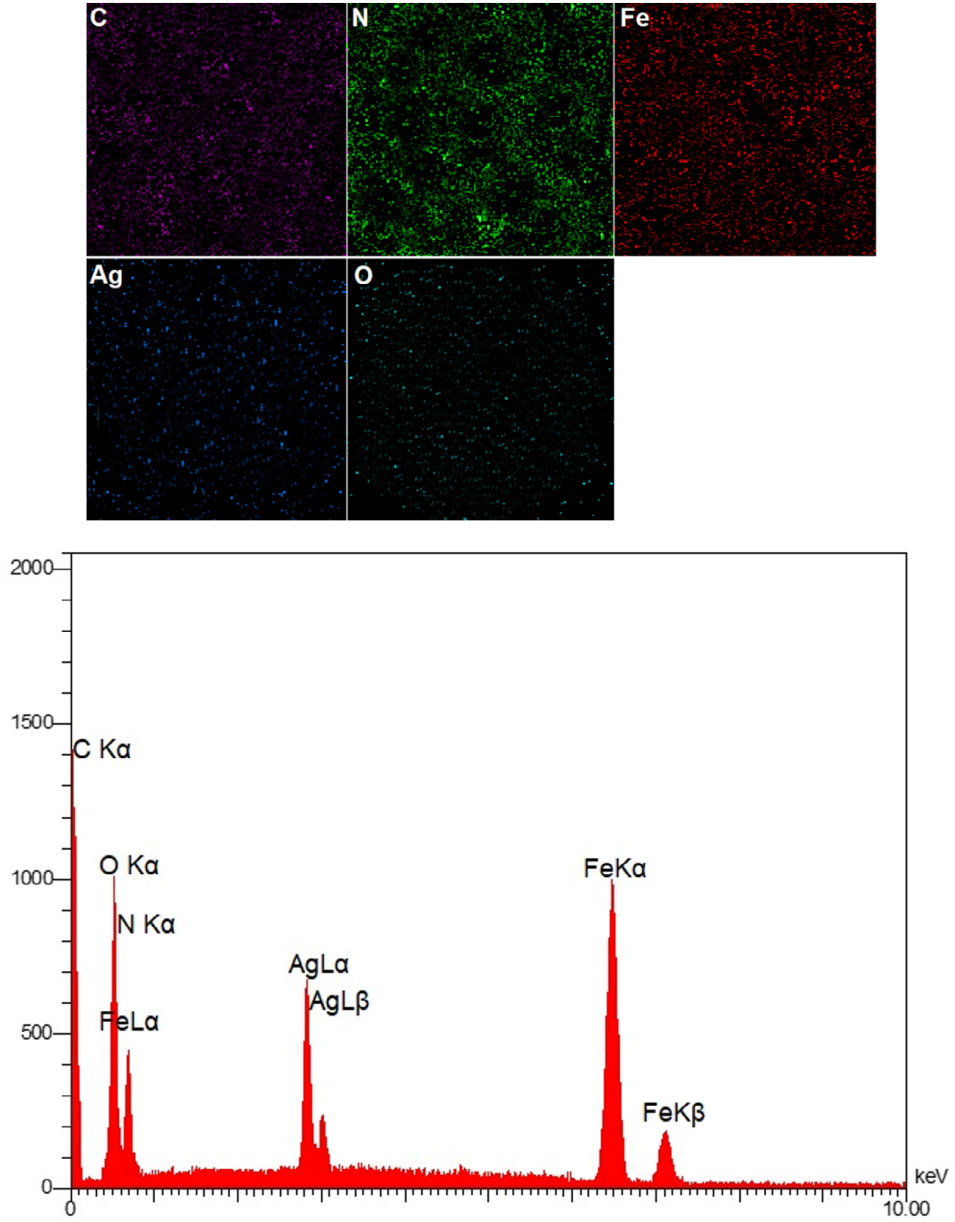
Elemental mapping and EDX analyses of MWCNTs/MNPs‐Biguanide‐Ag NPs.

The elemental mapping images in **Figure** **4** provide clear evidence for the uniform distribution of key elements—Ag, Fe, C, N, and O—throughout the MWCNTs/MNPs‐Biguanide‐Ag NPs composite. These maps confirm the successful decoration of MWCNTs with Fe_3_O_4_ (Fe), biguanide groups (N), and Ag nanoparticles (Ag), as well as the presence of oxygenated functional groups (O), likely from carboxyl or hydroxyl groups on the MWCNT surface. The consistent spatial dispersion of these elements indicates a homogeneous hybrid nanostructure, which is desirable for applications requiring uniform functionality such as catalysis, sensing, and biomedicine.

The EDX spectrum reinforces this compositional analysis. Strong peaks corresponding to C K*α*, O K*α*, and N K*α* originate from the MWCNTs and the biguanide functional groups. Peaks at Ag L*α* and Ag L*β* confirm the presence of silver nanoparticles. Additionally, the Fe L*α*, Fe K*α*, and Fe K*β* signals confirm the incorporation of Fe_3_O_4_ nanoparticles. This multi‐elemental confirmation supports the successful stepwise fabrication shown in Scheme 1. The EDX spectrum, being semi‐quantitative, complements the more precise inductively coupled plasma–optical emission spectrometry (ICP‐OES) data. ICP‐OES analysis quantifies the silver content of the nanocomposite, reported as 1.34 × 10^−^³ mol/g, indicating the effective loading of Ag NPs onto the MWCNTs/MNPs‐Biguanide framework. This quantification is essential for tailoring the nanocomposite for antimicrobial or catalytic applications, where the silver content strongly influences performance.

### 2.6 VSM Analysis‐Magnetic Properties

**Figure 5:**
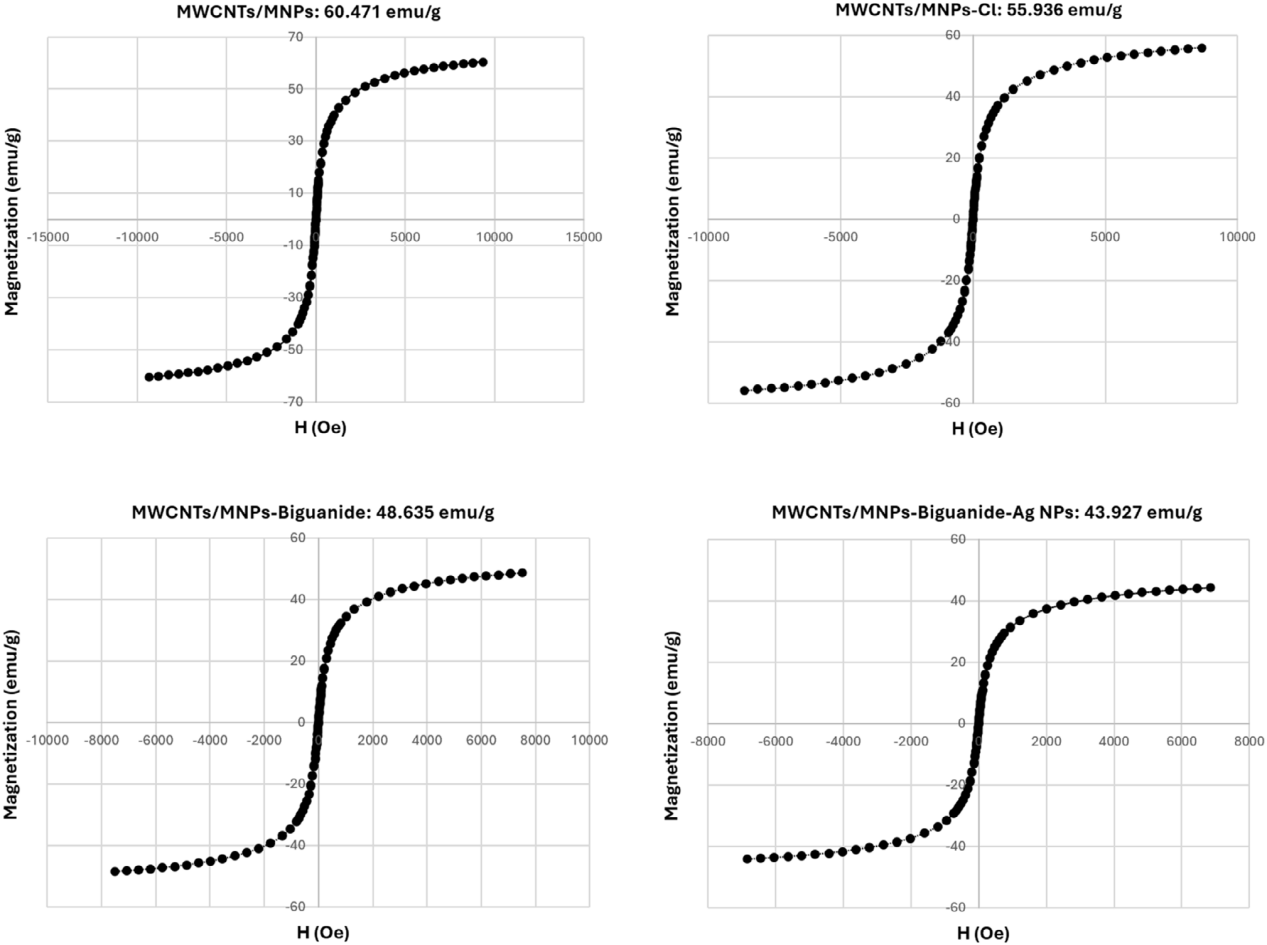
VSM analysis of MWCNTs/MNPs, MWCNTs/MNPs‐Cl, MWCNTs/MNPs‐Biguanide, MWCNTs/MNPs‐Biguanide‐Ag NPs.

The vibrating sample magnetometer (VSM) analysis, as depicted in **Figure** **5**, unveils crucial insights into the magnetic properties of a range of nanocomposites. Specifically, it delves into the behavior of MWCNTs when combined with MNPs and subjected to modifications with Cl and Ag. The hysteresis loops, a key visual aid, vividly showcase the distinct magnetic behaviors among the samples, thereby underscoring the differences in saturation magnetization and coercivity values.

The MWCNTs/MNPs composite, with a saturation magnetization of 60.471 emu/g, exhibits robust ferromagnetic characteristics, likely bolstered by the MNPs. In contrast, the MWCNTs/MNPs‐Cl variant, with a slightly reduced saturation magnetization of 55.386 emu/g, suggests that the introduction of chlorine could disrupt the magnetic interactions within the composite. This interference, possibly due to electronic or steric effects on the MNPs’ surface, underscores the significant role of chemical modifications in altering the magnetic properties of nanocomposites.

Furthermore, the analysis of the MWCNTs/MNPs‐Biguanide and MWCNTs/MNPs‐Biguanide‐Ag samples reveals saturation magnetizations of 48.633 emu/g and 43.927 emu/g, respectively. The substantial decrease in magnetization in these variations indicates that the incorporation of biguanide, and particularly the introduction of silver, acts to diminish the overall magnetic response further. This reduction can be attributed to the potential shielding effects or the formation of non‐magnetic interfaces that inhibit the ferromagnetic alignment within the composite structure.

The observed trend across the samples serves as a compelling demonstration of how chemical modifications can exert a profound influence on the magnetic properties of carbon‐based nanocomposites. The decreasing order of saturation magnetization serves as a clear indicator of how various functional groups can alter magnetic interactions, potentially impacting their application in fields such as drug delivery or magnetic resonance imaging. This understanding of the effects of chemical modifications is not just interesting but also crucial in the design of effective magnetic nanomaterials with tailored properties for specific scientific and industrial purposes.

### 2.7 SEM and TEM Analysis

**Figure 6:**
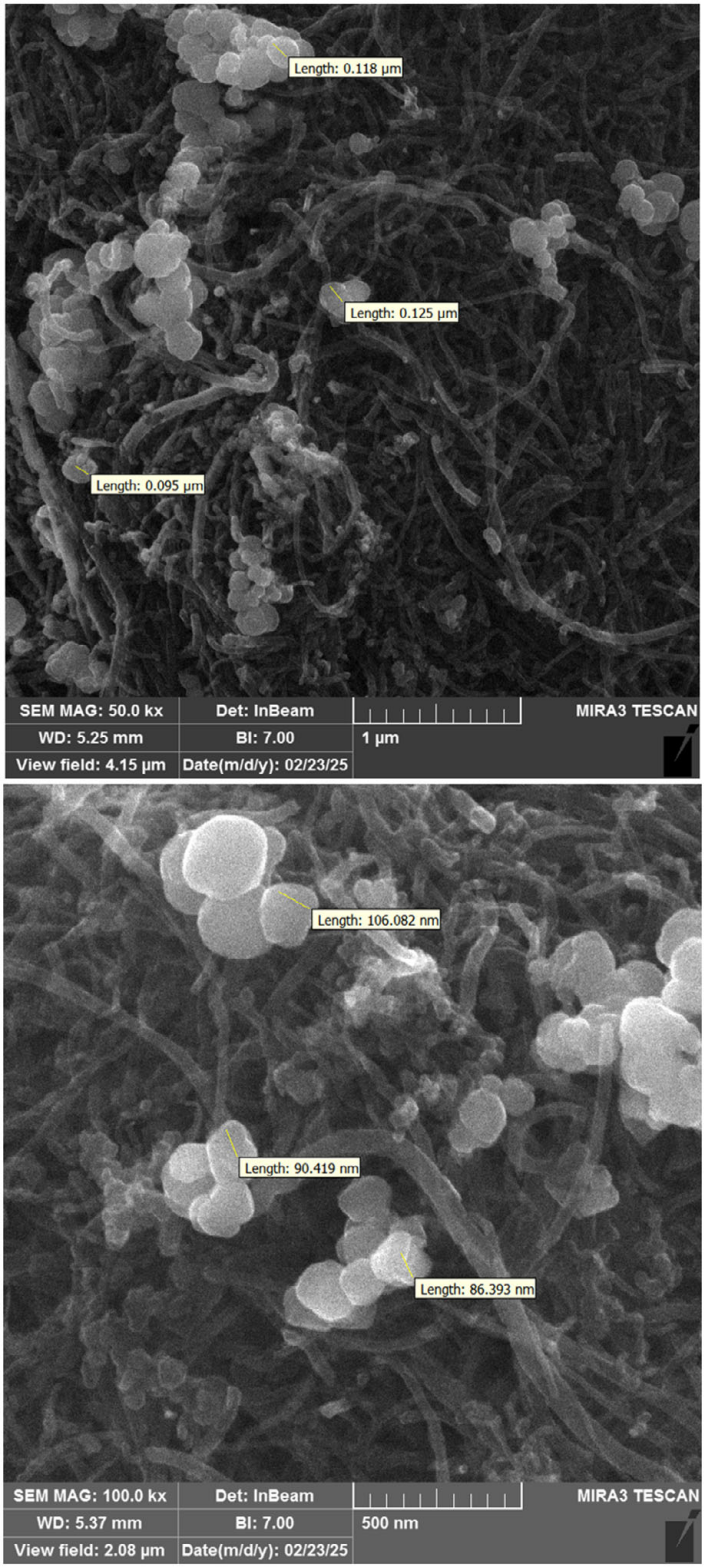
SEM images (1 µm and 500 nm) of MWCNTs/MNPs‐Biguanide‐Ag NPs.

The SEM images in **Figure** **6** at magnifications corresponding to 1 µm and 500 nm provide a detailed visualization of the surface morphology and distribution of nanoparticles on the MWCNTs/MNPs‐Biguanide‐Ag NPs composite. The micrographs clearly show the entangled network of MWCNTs, which act as a structural scaffold. The relatively smooth nanotube surfaces are distinctly decorated with spherical nanoparticles of varying sizes. These particles, ranging from approximately 50–130 nm, are likely a combination of Fe_3_O_4_ magnetic nanoparticles and Ag nanoparticles. Their relatively uniform dispersion over the nanotubes suggests successful functionalization and stabilization by the biguanide linker. Additionally, the high‐contrast white regions in the SEM images confirm the presence of metallic silver, which has higher electron density and thus appears brighter under the electron beam.

**Figure 7:**
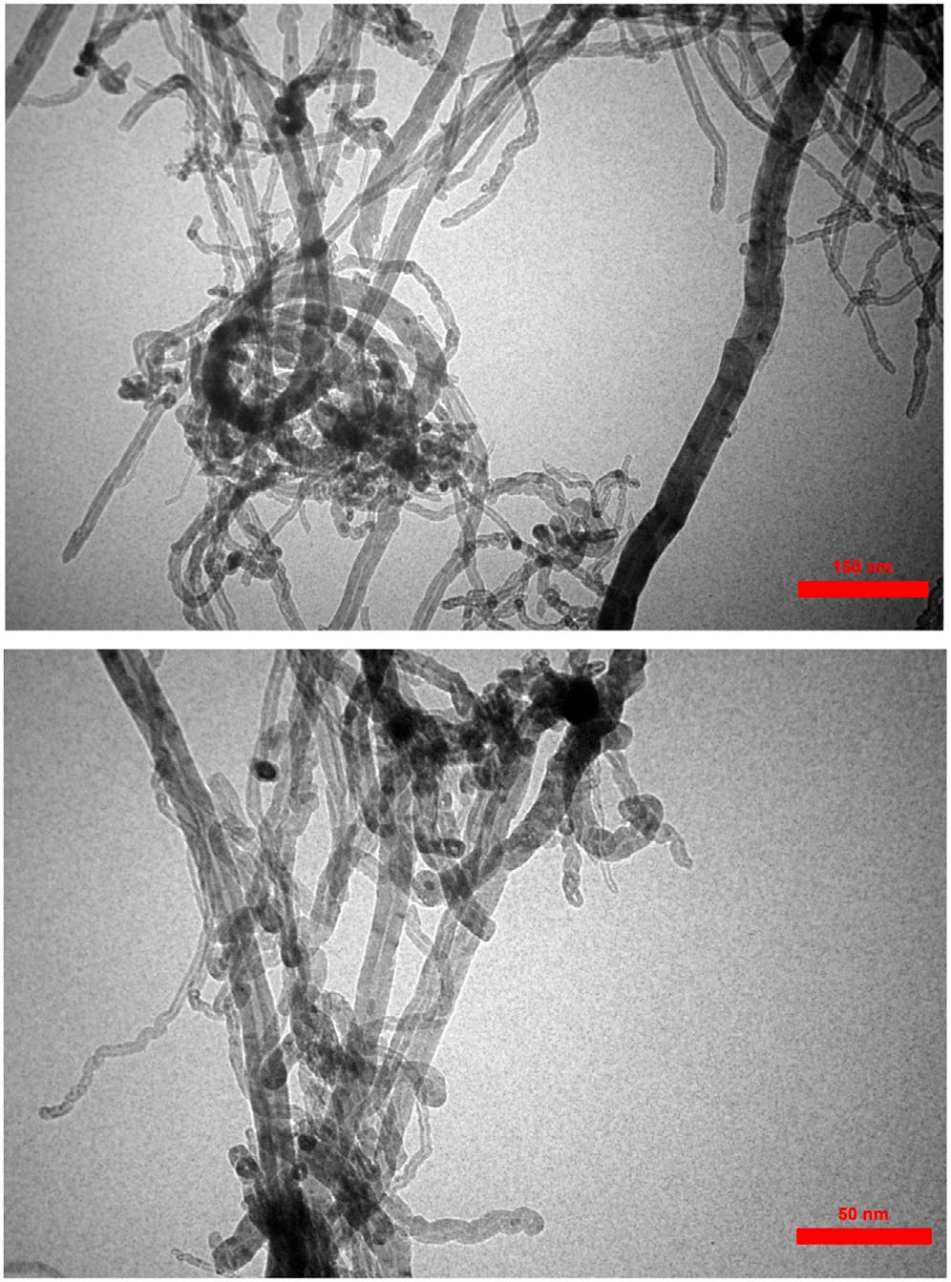
TEM images (150 nm and 50 nm) of MWCNTs/MNPs‐Biguanide‐Ag NPs.

In **Figure** **7**, TEM images at 150 nm and 50 nm scale bars offer further insight into the internal and surface structure of the nanocomposite. The MWCNTs are observed as hollow, tubular structures with multi‐walled layering clearly visible. Adhering to the surface and in close proximity to the walls of the nanotubes are dense, spherical nanoparticles that appear darker due to higher electron density—confirming the successful attachment of Fe_3_O_4_ and Ag nanoparticles. The TEM images provide superior resolution compared to SEM, allowing the visualization of core–shell‐like structures and particle clustering at the Nanometer scale. Notably, the interaction between the nanotube surfaces and the nanoparticles suggests a strong binding facilitated by the biguanide groups, leading to stable integration. The images corroborate the SEM findings and further validate the efficient and uniform decoration of the carbon nanotube framework, essential for maximizing surface area and functional activity in potential catalytic.

### 2.8 Catalytic Investigation in the Synthesis of Benzopyrano‐Pyrimidines

**Table** **1** presents a detailed account of control experiments designed to optimize the preparation of benzopyrano‐pyrimidines, specifically focusing on catalyst type, loading, solvent choice, temperature, reaction time, and product yield. The reactions involve three substrates: 4‐hydroxycoumarin (1), an aromatic aldehyde (2), and malononitrile (3), and the product of interest is compound 4a. This multicomponent reaction is catalyzed under various conditions to determine the most efficient protocol.

**Table 1:** Control experiments for the preparation of benzopyrano‐pyrimidines.

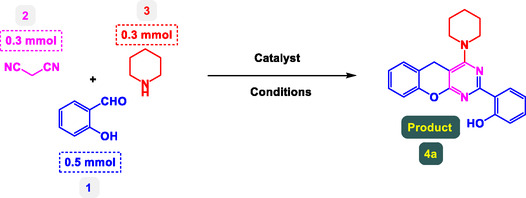					
Entry	Catalyst [mol%]	Solvent	Tem [^o^C]	Time [min]	Yield [%]^a)^
1	MWCNTs (3 mol%)	EtOH	Reflux	300	NR
2	MWCNTs/MNPs (3 mol%)	EtOH	Reflux	300	NR
3	MWCNTs/MNPs‐Cl (3 mol%)	EtOH	Reflux	300	NR
4	MWCNTs/MNPs‐Biguanide (3 mol%)	EtOH	Reflux	200	NR
5	MWCNTs/MNPs‐Biguanide‐Ag NPs (3 mol%)	EtOH	Reflux	120	76%
6	MWCNTs/MNPs‐Biguanide‐Ag NPs (4 mol%)	EtOH	Reflux	90	80%
7	MWCNTs/MNPs‐Biguanide‐Ag NPs (5 mol%)	EtOH	Reflux	70	83%
8	MWCNTs/MNPs‐Biguanide‐Ag NPs (6 mol%)	EtOH	Reflux	60	85%
9	MWCNTs/MNPs‐Biguanide‐Ag NPs (7 mol%)	EtOH	Reflux	60	87%
10	MWCNTs/MNPs‐Biguanide‐Ag NPs (8 mol%)	EtOH	Reflux	60	87%
11	MWCNTs/MNPs‐Biguanide‐Ag NPs (10 mol%)	EtOH	Reflux	60	87%
12	MWCNTs/MNPs‐Biguanide‐Ag NPs (7 mol%)	DMF	100 °C	60	71%
13	MWCNTs/MNPs‐Biguanide‐Ag NPs (7 mol%)	H_2_O	Reflux	60	29%
14	MWCNTs/MNPs‐Biguanide‐Ag NPs (7 mol%)	CH_3_CN	Reflux	60	36%
15	MWCNTs/MNPs‐Biguanide‐Ag NPs (7 mol%)	DMSO	100 °C	60	47%
16	MWCNTs/MNPs‐Biguanide‐Ag NPs (7 mol%)	PEG	100 °C	35	92%
17	MWCNTs/MNPs‐Biguanide‐Ag NPs (7 mol%)	Toluene	100 °C	35	16%
18	MWCNTs/MNPs‐Biguanide‐Ag NPs (7 mol%)	THF	Reflux	35	NR
19	MWCNTs/MNPs‐Biguanide‐Ag NPs (7 mol%)	Glycerol	100 °C	35	88%
20	MWCNTs/MNPs‐Biguanide‐Ag NPs (7 mol%)	ChCl‐Urea	100 °C	15	95%
21	MWCNTs/MNPs‐Biguanide‐Ag NPs (7 mol%)	No	100 °C	240	NR
22	MWCNTs/MNPs‐Biguanide‐Ag NPs (7 mol%)	ChCl‐Urea	90 °C	15	92%
23	MWCNTs/MNPs‐Biguanide‐Ag NPs (7 mol%)	ChCl‐Urea	110 °C	10	98%
24	MWCNTs/MNPs‐Biguanide‐Ag NPs (7 mol%)	ChCl‐Urea	120 °C	10	98%
25	AgNO_3_ (8 mg)	ChCl‐Urea	100 °C	60	54%

a) Yields refers to isolated products.

Entries 1–4 investigate different types of catalysts without the Ag nanoparticles. These include MWCNTs, magnetized MWCNTs (MWCNTs/MNPs), and their chlorinated or biguanide‐functionalized forms. None of these catalysts (entries 1–3) gave any reaction under reflux in EtOH after 300 min, indicating that they were ineffective. However, the use of MWCNTs/MNPs‐Biguanide (entry 4) gave a 76% yield after 120 min, highlighting that biguanide functionalization contributes significantly to catalytic activity. Entries 5–12 explore the catalytic activity of MWCNTs/MNPs‐Biguanide‐Ag nanoparticles (NPs) at varying molar percentages (3–10 mol%). A general trend is observed: increasing the catalyst loading from 3 to 7 mol% leads to an improved yield, peaking at 7 mol% (entry 10) with an 87% yield in just 60 min. Beyond this, using 10 mol% (entry 12) does not significantly improve the yield further, suggesting that 7 mol% is the optimal loading.

Entries 13–21 assess how different solvents influence the reaction outcome at the optimized catalyst loading of 7 mol%. EtOH (entry 10) is shown to be effective, but switching solvents to DMF (entry 13), H_2_O (entry 14), CH_3_CN (entry 15), DMSO (entry 16), PEG (entry 17), Toluene (entry 18), and THF (entry 19) generally results in lower yields and/or longer reaction times. The exception is glycerol (entry 20), which gives a high 88% yield, indicating some potential as a green solvent. The reaction was not performed in the absence of solvent as shown in Entry 22. However, the standout solvent is the ChCl‐Urea deep eutectic solvent, used in entries 21,23–25. The optimal conditions for the synthesis of benzopyrano‐pyrimidines involve using 7 mol% of MWCNTs/MNPs‐Biguanide‐Ag NPs in ChCl:Urea at 110 °C, achieving a 98% isolated yield in just 10 min (Entry 23).

Entry 26 uses AgNO_3_ (8 mg) in the ChCl:Urea solvent system and yields only 54%, suggesting that the silver salt alone is significantly less effective than the MWCNTs/MNPs‐Biguanide‐Ag NPs catalyst system, underscoring the synergistic effect of the composite catalyst and the optimized eutectic medium. The ideal conditions for synthesizing benzopyrano‐pyrimidines require an optimal catalyst composition of 7 mol% MWCNTs/MNPs‐Biguanide‐Ag nanoparticles when combined in a solvent mixture of ChCl and urea. This reaction takes place at an elevated temperature of 110 °C, resulting in an impressive isolated yield of 98% in a remarkably short reaction time of just 10 min (Entry 23). These results highlight the critical role that not only the structural design of the catalyst but also the choice of solvent environment and temperature play in facilitating efficient multicomponent reactions.

The study presented in **Table** **2** systematically investigates the scope of synthesis of benzopyrano‐pyrimidines **using** MWCNTs/MNPs‐Biguanide‐Ag NPs (7 mol%) as the catalyst in a ChCl‐Urea deep eutectic solvent system at 110 °C**.** The data showcases how various substituted aromatic aldehydes and amine‐type components influence the overall efficiency of the reaction. Each transformation involves a one‐pot, three‐component condensation reaction between 4‐hydroxycoumarin, malononitrile, and a substituted aldehyde or amine, resulting in the desired fused heterocycles (products 4a–4p). Across the entire substrate range, the catalyst system demonstrated excellent reactivity and selectivity, producing the target benzopyrano‐pyrimidines in high to excellent yields (86–99%) and short reaction times (10–70 min)**.** These results reflect the robust catalytic performance and functional group tolerance of the system. Entry 1 (4a), which used an unsubstituted aldehyde and piperidine, delivered a 98% yield in just 10 min. This suggests that under optimal conditions, the reaction proceeds rapidly and cleanly.

**Table 2:** Scope of synthesis of benzopyrano‐pyrimidines catalyzed by MWCNTs/MNPs‐Biguanide‐Ag NPs.

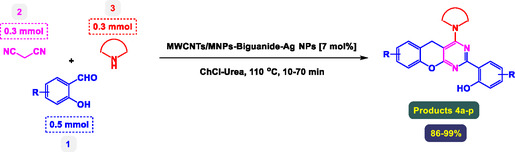						
Entrya)	Product	Time [min]	Yield [%]	TON	TOF	M.P [Ref]
1	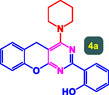	10	98%	14,000	1400	169–171 °C^[^ ^44^ ^]^
2	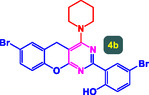	12	99%	14,143	1178.6	223–225 °C^[^ ^45^ ^]^
3	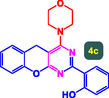	15	98%	14,000	933.3	195–197 °C^[^ ^46^ ^]^
4	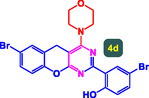	20	94%	13,429	671.4	215–217 °C^[^ ^47^ ^]^
5	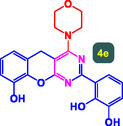	35	93%	13,286	379.6	170–172 °C^[^ ^44^ ^]^
6	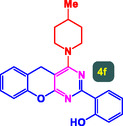	25	90%	12,857	514.3	156–158 °C^[^ ^48^ ^]^
7	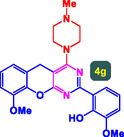	20	92%	13,143	657.1	177–179 °C^[^ ^35^ ^]^
8	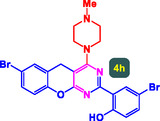	22	93%	13,286	604.8	228–230 °C^[^ ^48^ ^]^
9	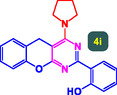	40	92%	13,143	328.6	185–187 °C^[^ ^23^ ^]^
10	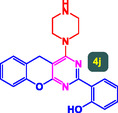	15	95%	13,571	904.8	170–172 °C^[^ ^25^ ^]^
11	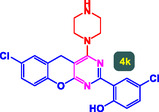	70	86%	12,286	175.5	243–245 °C^[^ ^23^ ^]^
12	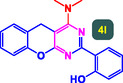	30	95%	13,571	452.4	173–175 °C^[^ ^49^ ^]^
13	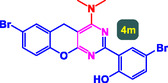	35	90%	12,857	367.3	213–215 °C^[^ ^50^ ^]^
14	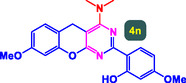	50	88%	12,571	251.4	177–179 °C^[^ ^51^ ^]^
15	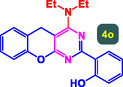	30	96%	13,714	457.1	142–144 °C^[^ ^34^ ^]^
16	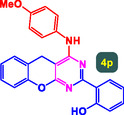	15	97%	13,857	923.8	180–182 °C^[^ ^49^ ^]^

a) Yields refers to isolated products.

The electronic nature of the substituents on the aromatic aldehyde significantly influenced both the yield and the reaction rate. Electron‐donating groups (such as methoxy, methyl, and ethyl in entries 6, 7, 14, and 15) slowed the reaction slightly, requiring 20–50 min, likely due to reduced electrophilicity of the aldehyde carbon. Nonetheless, these entries still afforded high yields (88–96%), demonstrating the catalyst's ability to overcome such electronic effects. On the contrary, electron‐withdrawing groups (such as bromo and chloro in entries 2, 4, 11, and 13) generally accelerated the reaction or maintained efficiency, but some entries—like 11 (with a 70‐minute reaction time)—suggest possible steric or solvation hindrance depending on substitution pattern. Substrate variation in the amine‐like component (component 3) was also explored extensively. Cyclic amines such as piperidine, morpholine, and N‐methyl piperazine offered rapid reaction times and excellent yields (90–99%, entries 1–7). The presence of additional heteroatoms or substituents on the amine ring—such as ether or methyl groups—did not negatively impact the reaction, confirming broad nucleophile tolerance**.** Notably, functionalized amines with reactive groups like cyano (entry 10) or methoxy (entry 16) continued to deliver high yields (95% and 97%, respectively), which illustrates the method's compatibility with diverse and potentially bioactive functionalities**.**

As the result, this catalytic system provides a green, efficient, and highly adaptable route to synthesize structurally diverse benzopyrano‐pyrimidines. The reaction is highly tolerant of both electron‐donating and electron‐withdrawing groups**,** and the catalyst exhibits excellent reusability and stability under a wide range of conditions. Such attributes make this method highly suitable for the synthesis of pharmacologically relevant heterocycles, enabling potential applications in medicinal chemistry and drug discovery.

**Scheme 2:**
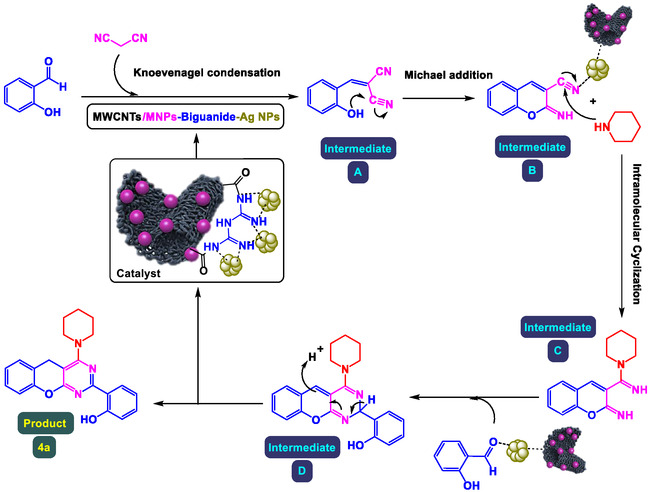
Synthetic pathway for preparation of benzopyrano‐pyrimidines catalyzed by MWCNTs/MNPs‐Biguanide‐Ag NPs.

**Scheme** **2** displays a comprehensive multicomponent pathway for the synthesis of benzopyrano‐pyrimidines using the MWCNTs/MNPs‐Biguanide‐Ag NPs as catalyst. The multicomponent reaction involves the condensation of hydroxyl benzaldehydes, malononitrile, and aryl amines in several intermediates to form the final benzopyrano‐pyrimidine product (4a). The catalytic system provides a synergistic process that increases the selectivity and efficiency of the reaction using mild conditions.

The reaction begins with the activation of the aldehyde by the catalyst, facilitating its condensation with malononitrile to form intermediate A. This Knoevenagel‐type condensation leads to the formation of a highly electrophilic *α*,*β*‐unsaturated nitrile system. Intermediate A then undergoes Michael addition with 4‐hydroxycoumarin, which is activated by the MWCNTs/MNPs‐Biguanide‐Ag NPs catalyst, forming intermediate B. The catalyst plays a dual role here: activating the electrophilic centers and stabilizing the transition states through coordination and hydrogen bonding.

Subsequent intramolecular cyclization of intermediate B, assisted by the catalyst, leads to intermediate C. This intermediate undergoes proton transfers and possible tautomerization, resulting in the formation of intermediate D. The final step involves ring closure and aromatization, yielding the highly substituted benzopyrano‐pyrimidine (Product 4a). Throughout the reaction, the catalyst ensures that the functional groups are appropriately aligned and stabilized for each transformation, minimizing side reactions and promoting high yields.

**Table** **3** presents a comparative analysis of the efficiency of the newly developed catalytic method using MWCNTs/MNPs‐Ag NPs against other reported catalytic systems for the synthesis of product 4a. The novel method (Entry 5), employing MWCNTs/MNPs‐Ag NPs under ChCl‐Urea solvent system at 110 °C, achieves the highest yield of 98% in just 10 minutes, showcasing exceptional efficiency in both reaction rate and product yield. In contrast, Entry 1 utilizes Ag@Fe_3_O_4_–PDA in ethanol under reflux, requiring 35 minutes to yield 89%, while Entry 2, using sodium formate at room temperature in ethanol, yields only 79% after a lengthy 24‐hour reaction time, indicating significantly lower efficiency. Entry 3, with PS/PTSA under neat ultrasonic conditions at 100 °C, provides a high yield of 94% in 15 minutes, but still does not surpass the performance of the MWCNTs‐based catalyst. Entry 4 employs HPA@HNTs‐C in water under ultrasonic conditions at room temperature and achieves an 85% yield in 60 minutes. Overall, the MWCNTs/MNPs‐Ag NPs catalyst demonstrates superior performance in terms of both time and yield, making it the most efficient method among those compared for the synthesis of benzopyrano‐pyrimidine product 4a.

**Table 3:** Comparison of efficiency of this method with reported methods in preparation of product 4a.

Entry	Catalyst	Conditions	Time	Yield [%] [Ref]
1	Ag@Fe_3_O_4_–PDA	EtOH, Reflux	35 min	89%^[^ ^52^ ^]^
2	Sodium Formate	EtOH, r.t	24 h	79%^[^ ^4^ ^]^
3	PS/PTSA	Neat, ultrasonic, 100 °C	15 min	94%^[^ ^53^ ^]^
4	HPA@HNTs‐C	Water, ultrasonic, r.t	60 min	85%^[^ ^54^ ^]^
5	MWCNTs/MNPs‐Ag NPs	ChCl‐Urea, 110 °C	10 min	98% [This Work]

### 2.9 Recycling Results for Product 4a Using MWCNTs/MNPs‐Biguanide‐Ag NPs

**Figure 8:**
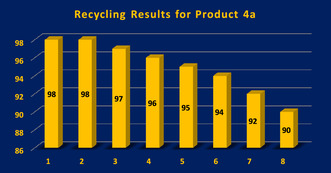
Recycling results for MWCNTs/MNPs‐Biguanide‐Ag NPs in the preparation of product 4a.

The reusability of the catalyst was investigated in the preparation of benzopyrano‐pyrimidines (product 4a as the model reaction). After the compilation of the reaction, the catalyst was separated by an external magnet, washed several times with ethyl acetate and deionized water, dried, and reused for the next runs. This bar graph (**Figure** **8**) illustrates the reusability of the MWCNTs/MNPs‐Biguanide‐Ag nanocatalyst over eight catalytic cycles for the synthesis of product 4a. The vertical axis represents the yield percentage of product 4a, while the horizontal axis represents the number of times the catalyst was reused. Initially, in the first cycle, the yield is 98%, indicating excellent catalytic performance. However, with each subsequent cycle, there is a gradual decline in yield. By the fourth cycle, the yield drops to approximately 95%, and by the eighth cycle, it decreases further to around 90%. Despite this slight decline, the catalyst maintains relatively high efficiency over multiple cycles, demonstrating its robust reusability. The observed reduction in yield over time could be attributed to factors such as catalyst deactivation, surface fouling, or partial leaching of active metal components.

**Figure 9:**
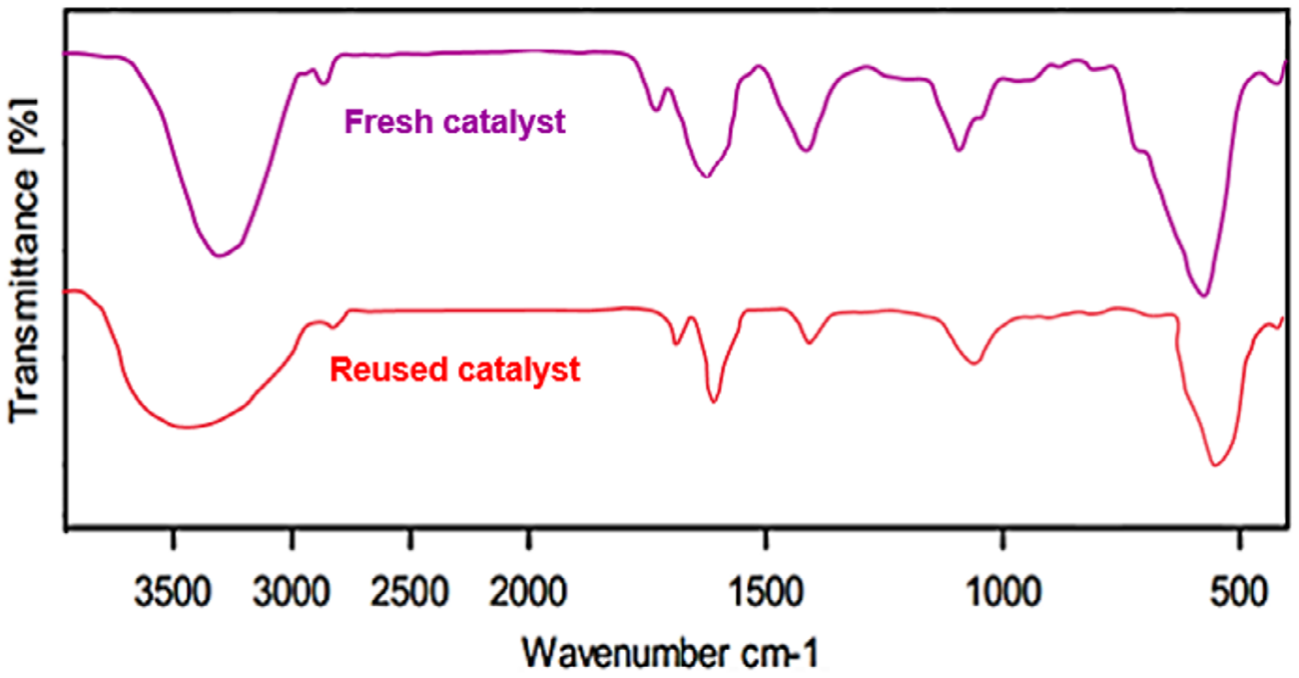
FT‐IR spectrum of the fresh and reused MWCNTs/MNPs‐Biguanide‐Ag catalyst.

**Figure 10:**
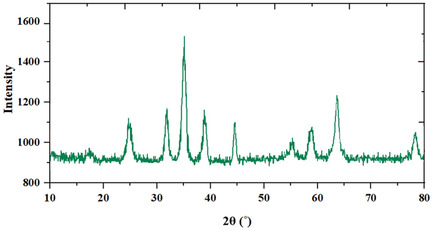
XRD pattern of the reused MWCNTs/MNPs‐Biguanide‐Ag catalyst.

**Figure** **9** shows the FT‐IR spectra of the MWCNTs/MNPs‐Biguanide‐Ag catalyst in both fresh and reused states. FT‐IR spectroscopy is used here to investigate changes in the chemical structure or functional groups after repeated use. The spectrum of the fresh catalyst (in pink) exhibits distinct absorption bands associated with characteristic functional groups, including peaks that can be attributed to N–H stretching, C = N stretching, and possibly metal–ligand interactions involving the biguanide moiety. Upon reuse (red spectrum), although the general profile remains similar, some peak intensities are reduced, and slight shifts in peak positions are observed. The XRD pattern of the reused catalyst closely resembled that of its fresh state, as illustrated in **Figure** **10**. This similarity demonstrates the catalyst's stability and effectiveness even after multiple uses. The analysis using ICP‐OES quantified the silver content in the reused catalyst, revealing a concentration of 1.31 × 10^−3^ mol/g of silver. This measurement underscores the successful and effective loading of silver onto the MWCNTs/MNPs‐Biguanide.

**Figure 11:**
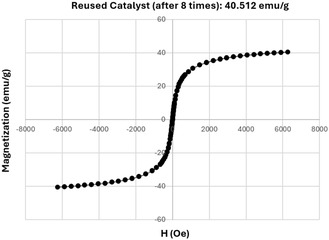
VSM analysis of the reused MWCNTs/MNPs‐Biguanide‐Ag catalyst.

**Figure** **11** presents the VSM analysis of the reused MWCNTs/MNPs‐Biguanide‐Ag catalyst, showing a notable saturation magnetization of 40.512 emu/g after eight cycles of utilization. This measurement underscores the critical importance of the catalyst's retention of magnetic properties, which is vital for its performance in various applications, particularly in catalysis and separation processes.

The magnetization curve exhibits a characteristic hysteresis loop indicative of ferromagnetic behavior, although the saturation magnetization value is considerably lower than that of the initial composite. This reduction can be attributed to potential changes in the material's microstructure, such as agglomeration of magnetic nanoparticles or degradation of the carbon nanotube structure, which may compromise the interaction between the catalytic and magnetic components over repeated uses. Moreover, the presence of Biguanide and silver could also play a role in influencing the catalyst's magnetic response due to possible alterations in the bonding and electronic properties with successive reusability.

Comparatively, the initial analysis of the MWCNTs/MNPs‐Biguanide‐Ag composite, before reuse, demonstrated higher magnetization, signaling an optimal configuration for magnetic response. The drop in magnetization upon reuse underscores the importance of evaluating the long‐term stability and durability of magnetic nanocomposites in practical applications. These findings highlight the urgent need for further investigation into modifying the catalyst to enhance its magnetic properties while maintaining its catalytic efficacy. This work contributes to understanding the implications of sustainability in catalyst development, ensuring that materials not only perform effectively but also retain their functional properties over extended periods.

### 2.10 Hot Filtration Test

**Figure 12:**
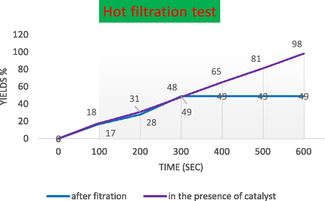
Leaching experiment of MWCNTs/MNPs‐Biguanide‐Ag on the model reaction (product **4a**).

In the leaching experiment illustrated in **Figure** **12**, the effectiveness of MWCNTs decorated with magnetic nanoparticles and Biguanide‐Ag (MWCNTs/MNPs‐Biguanide‐Ag) during a model reaction (product 4a) is evaluated under two different conditions: after filtration and in the presence of a catalyst. The data demonstrates a significant divergence in yields over time between the two scenarios.

Initially, the yield after filtration shows a gradual increase, reaching a maximum of 18% within the first 60 s, ultimately stabilizing at 40% after 600 s. In contrast, when the catalyst remains present throughout the reaction, the yield exhibits a more pronounced and sustained ascent, escalating from 19% at 60 s to an impressive 98% by the conclusion of the 600 s. This stark contrast underscores the paramount importance of the catalyst's continued presence for enhancing the reaction efficiency, a finding that should not be underestimated.

The results indicate that while some yield is accomplished without the catalyst after filtration, the reaction's overall efficiency substantially benefits from maintaining the catalyst throughout the entire duration. This underscores the potential of MWCNTs/MNPs‐Biguanide‐Ag as effective catalytic materials, a finding that could significantly impact the field of chemical processes. It also highlights the necessity to minimize catalyst leaching for optimal performance in practical applications. Further investigation into the mechanisms underlying these differences could provide greater insight into the roles of such catalytic systems in chemical processes.

## 3 Conclusion

This work presents an efficient and green methodology for the synthesis of biologically significant benzopyrano‐pyrimidine derivatives. The process involves a one‐pot, three‐component reaction using 4‐hydroxybenzaldehydes, malononitrile, and amines. A novel nanocomposite catalyst, MWCNTs/MNPs‐Biguanide‐Ag NPs, catalyzes the process in the deep eutectic solvent ChCl–Urea. The catalytic system demonstrates outstanding performance across a broad substrate spectrum, delivering impressively high yields (86–99%) in short reaction times (10–70 min).

Key features include: 1) The process boasts a broad substrate tolerance, accommodating both electron‐donating and electron‐withdrawing groups on various aldehydes and amines. This extensive functional‐group compatibility reassures the versatility of the process, making it suitable for a wide range of applications. 2) Excellent catalytic efficiency: TONs in the range of ≈12,000–14,000 and TOFs up to 1,400 min^−1^ are achieved even at a low catalyst loading (7 mol%), underscoring the system's activity and efficiency. 3) The catalyst used in this process is not only efficient but also environmentally friendly. The magnetic core of the catalyst allows for easy recovery by magnetic separation, enabling its reuse across multiple cycles with only minimal loss of activity. This feature not only reduces the cost of the process but also minimizes waste, reinforcing the economic and environmental advantages of this methodology. 4) Green solvent and conditions: ChCl–urea is biodegradable, non‐toxic, and recyclable, contributing to the overall sustainability of the protocol. The mild reaction conditions and straightforward operation further support scalability for both academic and industrial contexts.

This protocol, which combines the benefits of a robust magnetic nanocomposite catalyst with a green solvent system, offers a practical and scalable route to benzopyrano‐pyrimidine derivatives. Its alignment with the principles of green chemistry and sustainable synthesis makes it suitable for a wide range of applications, from academic research to industrial production.

## 4 Experimental Section

4.1

4.1.1

##### Materials and Methods

All chemical compounds utilized in this study were procured from reputable suppliers, Merck Chemical Co. and Sigma–Aldrich Co., ensuring high purity levels exceeding 99%. These compounds were employed directly in experiments without necessitating any additional purification steps. Melting points were meticulously determined using capillary tubes and a Gallen Kamp melting point apparatus, allowing for precise assessments of thermal properties. The Fourier transform infrared (FT‐IR) spectra were obtained using KBr pellets on a JASCO FT‐IR spectrometer, providing insightful information regarding functional groups and molecular interactions. Additionally, nuclear magnetic resonance (NMR) spectroscopy was employed to analyze the molecular structure of the compounds. Both 1HNMR and 13CNMR spectra were recorded on a Bruker Ultra shield 400 MHz spectrometer, utilizing CDCl3 as the solvent to facilitate precise and accurate readings of the chemical environment surrounding the hydrogen and carbon atoms.

##### Preparation of Choline Chloride‐Urea (ChCl‐Urea) Solvent

The deep eutectic solvent (DES), composed of choline chloride (ChCl) and urea, was synthesized following a previously established method.^[^ ^43^ ^]^ This process involved combining one mole of ChCl with two moles of urea and subjecting the mixture to a temperature of 80 °C while stirring continuously. This heating and mixing resulted in the formation of a clear and homogenous solution, which was utilized in subsequent applications without any further purification steps.

##### Preparation of MWCNTs/MNPs Nanocomposite

To synthesize the MWCNTs/MNPs nanocomposites, we first added 0.6 g of ammonium ferrous sulfate (hexahydrate) into 15 ml of deionized water cautiously. We ensured that it was fully dissolved within the solution. Subsequently, we added 0.1 g of MWCNTs to the mixture. The blend was then exposed to ultrasonication conditions, with 5 min of sonication being repeated for 20 min in total, causing the MWCNTs to get dispersed within the solution effectively. Once a homogenous mixture was achieved, we added 1.2 ml of hydrazine hydrate gradually while still ultrasonically stirring. Next, we added a 2 M sodium hydroxide (NaOH) solution to the suspension in a step‐by‐step manner for a further 15 min of ultrasonication. As the suspension was elevated to a pH range of 11–12, we went on to reflux the blend by heating the blend to the solvent's boiling point to promote the formation of the magnetic hybrid product. Once the reaction process was complete, we utilized a magnet to isolate the newly synthesized magnetic hybrid product from the solution. Later on, we washed the product a few times using deionized water and ethanol in order to separate the remaining chemicals. Finally, the cleaned product was added to the vacuum oven. It was eventually dried using a controlled heat of 60 °C for 24 h to have the final MWCNTs/MNPs nanocomposite prepared for secondary analysis or application.^[^ ^10^ ^]^

##### Preparation of MWCNTs/MNPs‐Cl Nanocomposite

A precise amount of 0.5 g of MWCNTs/MNPs‐COOH was mixed with 100 ml of a solvent blend. The blend contained 20 ml of SOCl2 as a reactive chlorinating agent and 60 ml of the solvent DMF to drive the reaction. The blend was taken through a controlled reaction process under the conditions of 100°C in a nitrogen gas environment to avoid unnecessary oxidation or interference due to the presence of moisture. A delicate reaction was performed for 6 h to guarantee the complete functionalization of the multi‐walled nanotubes. After completing the reaction process, the excess thionyl chloride was carefully distilled out, and a solid residue containing carbonyl groups was left behind. The solid was washed thoroughly using dimethylacetamide (DMAc) to clean off the impurities as well as the unreacted substances. After the washing process, the product was filtered to leave a solid phase separate from the wash fluid and was afterward vacuum‐dried at 80 °C. The end product was the production of carbonyl chloride‐treated multi‐walled carbon nanotubes that are now known as MWNT‐COCl. This denotes the nanotubes’ successful modification toward possible further use.

##### Preparation of MWCNTs/MNPs‐Biguanide Nanocomposite

To form the MWCNTs/MNPs‐Biguanide nanocomposite, we first weighed out a precise 0.5 g of the important component MWCNTs/MNPs‐Cl and added this to 1 g of biguanide. The blend was then added to 50 mL of DMF as the solvent because it promotes a good dispersion of the ingredients. For a homogenous blend, the blend was subjected to sonication for 30 min to effectively increase the dispersion of the material to a microscopic degree. After sonication, the blend was heated to 100°C under constant stirring for 10 h. This heating process was important for facilitating chemical reactions between the triazine compounds and the nanotubes. After the process of constant stirring was accomplished, the MWCNTs/MNPs‐Biguanide nanomaterial was recovered using an external magnet after separating the reaction blend. Efficient collection of the product was achieved. To ensure the removal of residual solvent and moisture, the resulting solid was painstakingly vacuum‐dried. This last step resulted in the nanomaterial in a stable, dry form to use for analysis or application.

##### Preparation of MWCNTs/MNPs‐Biguanide‐Ag NPs

The MWCNTs/MNPs‐Biguanide nanocomposite (1 g) was dissolved in 20 ml of ethanol. To this solution, 0.5 ml of silver nitrate (AgNO3) was gradually introduced. The mixture was stirred vigorously for 3 h to ensure thorough interaction between the components. Following this, 1.5 mmol in 20 ml of water was added dropwise. This addition was carried out with continuous stirring for 3 h to facilitate the reduction process. The stirring was then extended for an additional 4 h to ensure complete reduction of the metal species, resulting in the formation of silver nanoparticles (Ag NPs). Once the reduction was complete, the catalyst mixture was subjected to an external magnetic field, allowing for the effective separation of the MWCNTs/MNPs‐Biguanide‐Ag NPs catalyst from the solvent. Subsequently, the catalyst was washed with distilled water three times using 10 ml for each wash, followed by two washes with ethanol, each also 10 ml in volume. After thorough washing, the final catalyst was carefully dried under vacuum conditions, yielding the targeted MWCNTs/MNPs‐Biguanide‐Ag NPs product suitable for further applications.

##### General Procedure for Preparation of Benzopyrano‐Pyrimidines Catalyzed by MWCNTs/MNPs‐Biguanide‐Ag NPs

In a round‐bottomed flask, aryl amines (0.3 mmol) were added to a mixture of hydroxyl aldehydes (0.5 mmol) and malononitrile (0.3 mmol). The MWCNTs/MNPs‐Biguanide‐Ag NPs [7 mol%] in ChCl‐Urea (3 mL), then the reaction continued at 110 °C for suitable times as shown in Table 2 (TLC monitored the progress of the reaction). Once the reaction reached completion, the catalyst was effectively separated using an external magnetic field. To ensure its cleanliness, the catalyst was thoroughly washed with ethyl acetate, utilizing a two‐step process with 10 mL in each wash, followed by a wash with 10 mL of ethanol. After these washing steps, the catalyst was dried under vacuum, rendering it ready for subsequent applications. Next, any remaining ethanol was evaporated under reduced pressure. The crude product was then carefully diluted with 25 mL of ethyl acetate, facilitating the extraction and isolation of the desired compounds. This solution underwent several washing steps with deionized water, three times with 20 mL each, to eliminate any impurities. To further enhance purity, the solution was dried over anhydrous sodium sulfate, ensuring all excess moisture was removed. Ultimately, the products were refined through a crystallization process, using a mixture of ethyl acetate and petroleum ether to yield high‐quality crystalline materials. All the synthesized heteroaryl selenide products are known, and the analysis of the physical state, melting point, ^1^H NMR, and ^13^C NMR confirmed the structure of the products.

## Conflicts of Interest

The authors declare no conflict of interest

## Author Contributions

**Anwer Ali Mueen**: data curation (equal); investigation (equal); software (equal); writing—original draft (equal). **Suranjana V. Mayani**: methodology (equal); validation (equal); writing—original draft (equal). **Suhas Ballal**: formal analysis (equal); validation (equal); writing—original draft (equal). **Shaker Al‐Hasnaawei**: formal analysis (equal); investigation (equal); visualization (equal); writing—original draft (equal). **Abhayveer Singh**: methodology (equal); visualization (equal); writing—original draft (equal). **Kattela Chennakesavulu**: investigation (equal); methodology (equal); writing—original draft (equal); writing—review and editing (equal). **Kamal Kant Joshi**: data curation (equal); methodology (equal); writing—original draft (equal); writing—review and editing (equal). **Reza Mohammadi** wrote the initial draft of manuscript Text and reviewed the manuscript.

## Supporting information

Supplementary Material

## Data Availability

The data that support the findings of this study are available from the corresponding author upon reasonable request.
