# Supplementary material for: A Robust Nanocatalyst Incorporating Multi‐Walled Carbon Nanotubes Infused with Magnetic Nanoparticles and Biguanide–Silver Nanoparticles for Multicomponent Synthesis of Benzopyrano‐Pyrimidines
Source: ChemistryOpen. 2025 Sep 12;14(12):e202500398. doi: 10.1002/open.202500398 (PMC12680572; doi:10.1002/open.202500398)
Supplement: Supplementary file 1 — Supplementary Material [file OPEN-14-e202500398-s001.pdf]

MWCNTs/MNPs-Biguanide-Ag NPs as a Robust Nanocatalyst for Sustainable and Efficient Multicomponent Synthesis of Benzopyrano-Pyrimidines

Anwer Ali Mueen [1], Suranjana V. Mayani [2], Suhas Ballal [3], Shaker Al-Hasnaawei [4], Abhayveer Singh [5], Kattela Chennakesavulu [6], Kamal Kant Joshi [7], **Reza Mohammadi [8]***

1

Anwer Ali Mueen

Al-Qadisiyah University, College of Dentistry, Department of Basic Sciences, Al-Qadisiyah, Iraq.

Mail: anwer.ali.mueen@qu.edu.iq

2

Suranjana V. Mayani

Marwadi University Research Center, Department of Chemistry, Faculty of Science, Marwadi University, Rajkot, Gujarat

Mail: suranjana.mayani@marwadieducation.edu.in

3

Suhas Ballal

Department of Chemistry and Biochemistry, School of Sciences, JAIN (Deemed to be University), Bangalore, Karnataka, India.

Mail: suhas@jainuniversity.ac.in

4

Shaker Al-Hasnaawei (a,b)

[a] College of Pharmacy, the Islamic University, Najaf, Iraq

[b] Department of medical analysis, Medical laboratory technique college, the Islamic University of Al Diwaniyah, Al Diwaniyah, Iraq

Mail: shakeralhasnawi@iunajaf.edu.iq

5

Abhayveer Singh

Centre for Research Impact & Outcome, Chitkara University Institute of Engineering and Technology, Chitkara University, Rajpura, 140401, Punjab, India

Mail: abhayveer_singh@outlook.com

6

Kattela Chennakesavulu

Department of Chemistry, Sathyabama Institute of Science and Technology, Chennai, Tamil Nadu, India, **Mail: chennakesavulureddy.chemistry@sathyabama.ac.in**

7

Kamal Kant Joshi (a, b)

(a) Department of Allied Science, Graphic Era Hill University, Dehradun, India.

(b) Adjunct Professor, Graphic Era Deemed to be University, Dehradun, Uttarakhand, India.

Mail: kkjoshi@gehu.ac.in

8

Reza Mohammadi
Department of Chemistry, Young Researchers and Elite Club, Tehran Branch, Islamic Azad University, Tehran, Iran.
Corresponding author: rmohammadichem@gmail.com

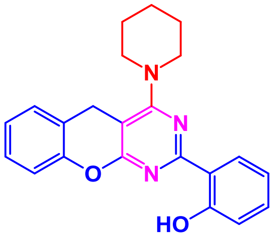

2-(4-(piperidin-1-yl)-5H-chromeno[2,3-d]pyrimidin-2-yl)phenol

(M.P: 169-171 ^{o}C)

^{1}H NMR (400 MHz, $CDCl_3$) δ 9.65 (s, 1H), 7.81 (d, *J* = 7.7 Hz, 1H), 7.61-7.53 (m, 1H), 7.48-7.42 (m, 1H), 7.33 (d, *J* = 8.0 Hz, 2H), 7.18-7.11 (m, 2H), 7.04-6.97 (m, 1H), 4.39 (s, 2H), 3.76-3.71 (m, 4H), 1.66-1.59 (m, 6H); ^{13}C NMR (100 MHz, $CDCl_3$) δ 174.5, 172.9, 160.1, 155.3, 150.1, 131.5, 130.8, 130.2, 126.3, 125.7, 124.6, 122.8, 122.4, 121.7, 121.1, 120.9, 101.6, 53.1, 27.5, 24.9, 23.0 ppm.

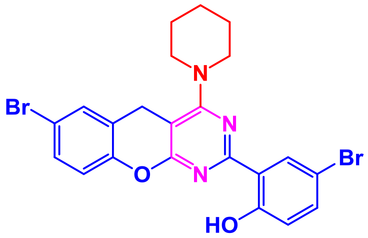

4-bromo-2-(7-bromo-4-(piperidin-1-yl)-5H-chromeno[2,3-d]pyrimidin-2-yl)phenol

(M.P: 223-225 ^{o}C)

^{1}H NMR (400 MHz, $CDCl_3$) δ 9.64 (s, 1H), 7.79 (d, *J* = 8.0 Hz, 1H), 7.65 (d, *J* = 8.6 Hz, 1H), 7.48 (s, 2H), 7.34 (d, *J* = 7.8 Hz, 1H), 7.11 (d, *J* = 9.1 Hz, 1H), 4.41 (s, 2H), 3.77-3.70 (m, 4H), 1.67-1.59 (m,

6H); ^{13}C NMR (100 MHz, $CDCl_3$) δ 174.9, 172.8, 158.1, 154.3, 149.8, 131.5, 133.7, 133.1, 132.6, 131.5, 131.0, 130.8, 130.4, 124.6, 122.7, 120.5, 118.3, 99.8, 56.7, 27.9, 26.0, 25.4 ppm.

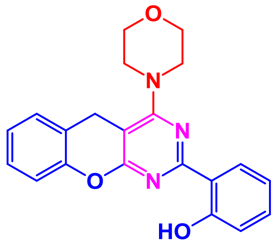

2-(4-morpholino-5H-chromeno[2,3-d]pyrimidin-2-yl)phenol

(M.P: 195-197 °C)

^{1}H NMR (400 MHz, $CDCl_3$) δ 9.63 (s, 1H), 7.77 (d, *J* = 7.4 Hz, 1H), 7.49-7.44(m, 1H), 7.23-7.18 (m, 2H), 7.01 (d, *J* = 7.8 Hz, 2H), 6.97-6.86 (m, 2H), 7.04-6.97 (m, 1H), 4.42 (s, 2H), 3.69-3.64 (m, 4H), 3.23-3.17 (m, 4H); ^{13}C NMR (100 MHz, $CDCl_3$) δ 174.9, 173.6, 159.4, 155.6, 150.1, 130.7, 129.4, 128.0, 126.5, 125.7, 123.4, 122.6, 121.2, 120.9, 120.5, 100.1, 65.9, 52.3, 25.7 ppm.

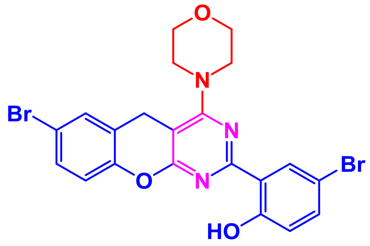

4-bromo-2-(7-bromo-4-morpholino-5H-chromeno[2,3-d]pyrimidin-2-yl)phenol

(M.P: 215-217 °C)

^{1}H NMR (400 MHz, $CDCl_3$) δ 9.63 (s, 1H), 7.88 (d, *J* = 8.6 Hz, 1H), 7.67 (d, *J* = 7.9 Hz, 1H), 7.54 (s, 1H), 7.47 (d, *J* = 8.8 Hz, 2H), 7.01 (d, *J* = 7.3 Hz, 1H), 4.51 (s, 2H), 3.88-3.82 (m, 4H), 3.43-3.31 (m, 4H); ^{13}C NMR (100 MHz, $CDCl_3$) δ 174.5, 172.7, 159.8, 155.6, 150.3, 132.1, 130.9, 130.1, 129.5, 128.0, 125.8, 125.2, 124.1, 123.7, 122.8, 121.5, 120.2, 118.9, 100.7, 66.0, 52.6, 28.7 ppm.

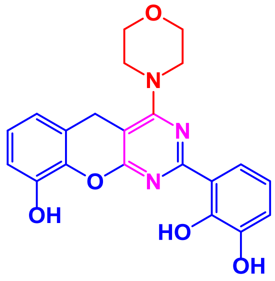

3-(9-hydroxy-4-morpholino-5H-chromeno[2,3-d]pyrimidin-2-yl)benzene-1,2-diol

(M.P: 170-172 °C)

^{1}H NMR (400 MHz, $CDCl_3$) δ 10.49 (s, 1H), 9.52 (s, 1H), 7.79 (d, *J* = 7.7 Hz, 1H), 7.53 (d, *J* = 8.0 Hz, 1H), 7.39-7.31 (m, 1H), 7.09-7.02 (m, 1H), 6.98-6.91 (m, 2H), 4.41 (s, 2H), 3.65-3.58 (m, 4H), 3.28-3.19 (m, 4H); ^{13}C NMR (100 MHz, $CDCl_3$) δ 174.2, 172.1, 158.0, 155.7, 145.3, 143.7, 142.5, 130.6, 124.3, 124.1, 123.9, 123.4, 122.0, 121.4, 119.5, 118.4, 113.2, 100.8, 67.1, 38.6, 28.3 ppm.

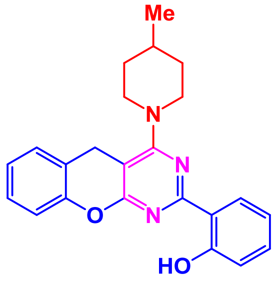

2-(4-(4-methylpiperidin-1-yl)-5H-chromeno[2,3-d]pyrimidin-2-yl)phenol

(M.P: 156-158 °C)

^{1}H NMR (400 MHz, $CDCl_3$) δ 9.65 (s, 1H), 7.76 (d, *J* = 8.5 Hz, 1H), 7.56-7.40 (m, 2H), 7.12-7.09 (m, 3H), 7.00-6.94 (m, 2H), 4.47 (s, 2H), 3.11-3.07 (m, 4H), 1.85-1.80 (m, 4H), 1.48-1.40 (m, 1H), 0.86 (d, *J* = 8.0 Hz, 3H); ^{13}C NMR (100 MHz, $CDCl_3$) δ 174.3, 172.1, 159.70, 155.0, 148.6, 130.9, 127.5, 124.8, 124.3, 123.2, 122.9, 122.6, 121.7, 121.3, 120.8, 117.5, 116.0, 113.9, 100.7, 51.7, 36.9, 33.4, 28.6, 18.1 ppm.

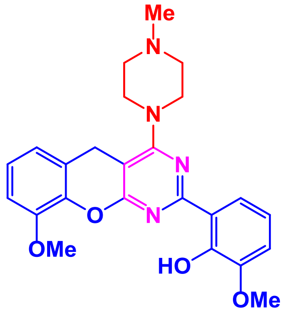

2-methoxy-6-(9-methoxy-4-(4-methylpiperazin-1-yl)-5H-chromeno[2,3-d]pyrimidin-2-yl)phenol

(M.P: 177-179 °C)

^{1}H NMR (400 MHz, $CDCl_3$) δ 9.61 (s, 1H), 7.84 (d, *J* = 7.6 Hz, 1H), 7.55-7.48 (m, 1H), 7.39 (d, *J* = 8.6 Hz, 1H), 7.23-7.16 (m, 1H), 7.00 (d, *J* = 7.4 Hz, 2H), 4.49 (s, 2H), 3.81 (s, 6H), 3.54-3.46 (m, 4H), 2.59-2.49 (m, 6H), 2.21 (s, 3H); ^{13}C NMR (100 MHz, $CDCl_3$) δ 174.7, 172.3, 158.3, 151.2, 150.7, 142.6, 140.8, 131.4, 124.5, 124.1, 123.7, 123.0, 122.6, 122.1, 120.3, 110.2, 100.6, 58.4, 57.1, 48.9, 47.0, 28.1 ppm.

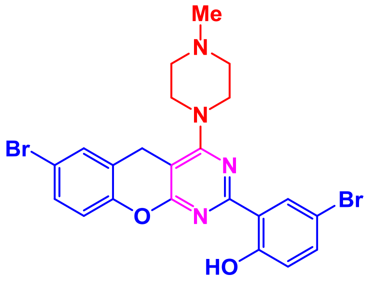

4-bromo-2-(7-bromo-4-(4-methylpiperazin-1-yl)-5H-chromeno[2,3-d]pyrimidin-2-yl)phenol

(M.P: 228-230 °C)

^{1}H NMR (400 MHz, $CDCl_3$) δ 9.62 (s, 1H), 7.79 (d, *J* = 7.2 Hz, 1H), 7.53 (d, *J* = 7.8 Hz, 1H), 7.44 (s, 1H), 7.23 (d, *J* = 8.0 Hz, 2H), 7.02 (d, *J* = 7.7 Hz, 1H), 4.45 (s, 2H), 3.83-3.76 (m, 4H), 2.56-2.47 (m, 4H), 2.23 (s, 3H); ^{13}C NMR (100 MHz, $CDCl_3$) δ 174.8, 172.2, 158.0, 155.6, 150.9, 133.1, 132.5, 131.0, 130.6, 126.7, 124.3, 124.1, 123.2, 122.8, 120.9, 120.1, 118.7, 100.6, 58.0, 47.2, 46.1, 28.6 ppm.

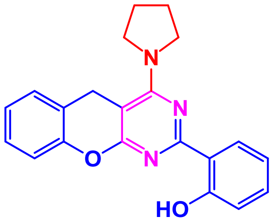

2-(4-(pyrrolidin-1-yl)-5H-chromeno[2,3-d]pyrimidin-2-yl)phenol

(M.P: 185-187 °C)

^{1}H NMR (400 MHz, $CDCl_3$) δ 9.60 (s, 1H), 7.81 (d, *J* = 7.3 Hz, 1H), 7.57-7.52 (m, 1H), 7.38-7.34 (m, 1H), 7.08 (d, *J* = 8.0 Hz, 2H), 6.95-6.79 (m, 3H), 4.39 (s, 2H), 3.78-3.72 (m, 4H), 1.94-1.88 (m, 4H); ^{13}C NMR (100 MHz, $CDCl_3$) δ 174.7, 172.6, 159.0, 155.8, 150.7, 130.1, 129.6, 128.1, 125.4, 125.1, 124.4, 124.2, 123.8, 122.3, 122.0, 121.1, 120.8, 100.1, 51.2, 47.0, 27.8 ppm.

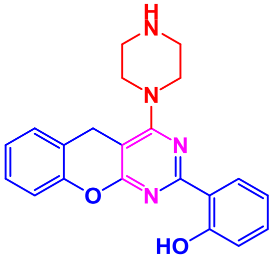

2-(4-(piperazin-1-yl)-5H-chromeno[2,3-d]pyrimidin-2-yl)phenol

(M.P: 170-172 °C)

^{1}H NMR (400 MHz, $CDCl_3$) δ 9.61(s, 1H), 7.82 (d, *J* = 7.0 Hz, 1H), 7.55-7.49 (m, 1H), 7.37-7.30 (m, 2H), 7.13-7.02 (m, 2H), 6.94-6.89 (m, 2H), 4.43 (s, 2H), 3.76-3.71 (m, 4H), 3.16-3.11 (m, 4H), 2.74-2.70 (m, 4H), 1.06 (s, 1H); ^{13}C NMR (100 MHz, $CDCl_3$) δ 174.5, 172.9, 160.1, 155.3, 150.1, 131.5, 130.8, 130.2, 126.3, 125.7, 124.6, 122.8, 122.4, 121.7, 121.1, 120.9, 101.6, 53.1, 27.5, 24.9, 23.0 ppm.

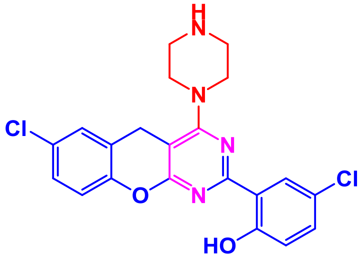

4-chloro-2-(7-chloro-4-(piperazin-1-yl)-5H-chromeno[2,3-d]pyrimidin-2-yl)phenol

(M.P: 243-245 °C)

^{1}H NMR (400 MHz, $CDCl_3$) δ 9.63 (s, 1H), 7.82 (s, 1H), 7.75 (d, *J* = 8.0 Hz, 2H), 7.41 (s, 1H), 7.29 (d, *J* = 7.6 Hz, 1H), 4.38 (s, 2H), 3.18-3.12 (m, 4H), 2.82-2.75 (m, 4H), 1.06 (s, 1H); ^{13}C NMR (100 MHz, $CDCl_3$) δ 174.5, 172.3, 160.1, 155.6, 150.3, 133.1, 129.5, 128.7, 127.6, 126.8, 126.2, 125.9, 125.5, 122.6, 119.7, 118.4, 100.7, 51.7, 47.9, 28.0 ppm.

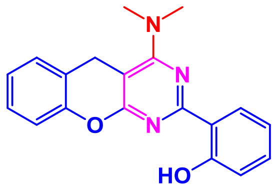

2-(4-(dimethylamino)-5H-chromeno[2,3-d]pyrimidin-2-yl)phenol

M.P: 173-175 °C)

^{1}H NMR (400 MHz, $CDCl_3$) δ 9.59 (s, 1H), 7.84 (d, *J* = 8.0 Hz, 1H), 7.75-7.66 (m, 1H), 7.49-7.43 (m, 2H), 7.19-7.05 (m, 4H), 4.47 (s, 2H), 3.09 (s, 6H); ^{13}C NMR (100 MHz, $CDCl_3$) δ 174.5, 172.3, 159.9, 155.2, 150.8, 130.5, 130.2, 129.7, 129.5, 128.1, 127.6, 127.3, 126.9, 124.8, 123.6, 123.2, 120.8, 119.5, 100.4, 40.8, 28.7 ppm.

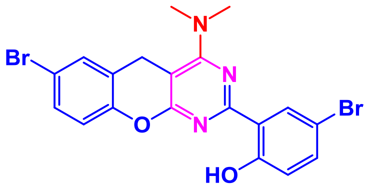

4-bromo-2-(7-bromo-4-(dimethylamino)-5H-chromeno[2,3-d]pyrimidin-2-yl)phenol

M.P: 213-215 °C)

^{1}H NMR (400 MHz, $CDCl_3$) δ 9.63 (s, 1H), 7.93 (d, *J* = 8.2 Hz, 1H), 7.85 (d, *J* = 8.6 Hz, 2H), 7.51 (s, 1H), 7.37 (s, 1H), 7.28 (d, *J* = 7.7 Hz, 2H), 4.44 (s, 2H), 3.05 (s, 6H); ^{13}C NMR (100 MHz, $CDCl_3$) δ 174.7, 172.8, 160.9, 156.8, 149.4, 131.2, 130.4, 130.1, 129.8, 128.4, 123.8, 122.6, 122.0, 121.8, 120.3, 118.7, 101.7, 40.1, 26.3 ppm.

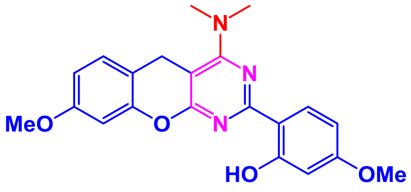

2-(4-(dimethylamino)-8-methoxy-5H-chromeno[2,3-d]pyrimidin-2-yl)-5-methoxyphenol

M.P: 177-179 °C)

^{1}H NMR (400 MHz, $CDCl_3$) δ 9.56 (s, 1H), 7.83 (d, *J* = 8.0 Hz, 1H), 7.49 (d, *J* = 7.8 Hz, 1H), 7.07 (s, 2H), 7.00 (d, *J* = 7.4 Hz, 1H), 6.91 (d, *J* = 7.8 Hz, 1H), 4.47 (s, 2H), 3.86 (s, 6H), 3.02 (s, 6H); ^{13}C NMR (100 MHz, $CDCl_3$) δ 174.3, 172.8, 162.9, 159.3, 156.7, 148.4, 130.5, 128.6, 111.6, 110.2, 108.4, 104.3, 100.7, 58.7, 40.3, 28.9 ppm.

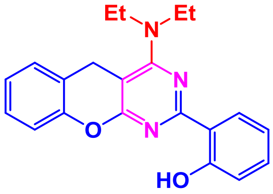

2-(4-(diethylamino)-5H-chromeno[2,3-d]pyrimidin-2-yl)phenol

M.P: 142-144 °C)

^{1}H NMR (400 MHz, $CDCl_3$) δ 9.64 (s, 1H), 7.82 (d, *J* = 7.6 Hz, 1H), 7.55-7.50 (m, 2H), 7.34-7.28 (m, 2H), 7.00-6.95 (m, 2H), 6.77-6.70 (m, 2H), 4.41 (s, 2H), 3.89-3.82 (m, 4H), 1.42-1.38 (m, 6H); ^{13}C NMR (100 MHz, $CDCl_3$) δ 174.9, 172.4, 160.5, 155.1, 150.8, 128.7, 127.6, 126.3, 124.1, 123.4, 1228, 122.1, 121.6, 121.2, 120.6, 119.5, 118.7, 101.6, 48.7, 25.4, 15.8 ppm.

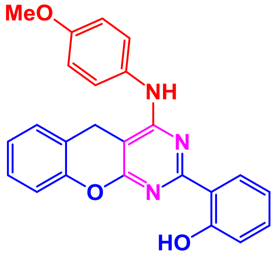

2-(4-((4-methoxyphenyl)amino)-5H-chromeno[2,3-d]pyrimidin-2-yl)phenol

M.P: 180-182 °C)

^{1}H NMR (400 MHz, $CDCl_3$) δ 9.55 (s, 1H), 7.81 (d, *J* = 8.2 Hz, 1H), 7.48-7.42 (m, 1H), 7.33-7.28 (m, 2H), 7.089-7.03 (m, 2H), 6.98-6.92 (m, 2H), 4.48 (s, 2H), 3.85 (s, 3H); ^{13}C NMR (100 MHz, $CDCl_3$) δ 174.5, 172.6, 160.7, 155.4, 150.3, 130.9, 129.5, 128.7, 127.3, 125.4, 123.6, 122.7, 121.9, 121.3, 120.2, 119.8, 118.6, 101.3, 58.4, 28.9 ppm.

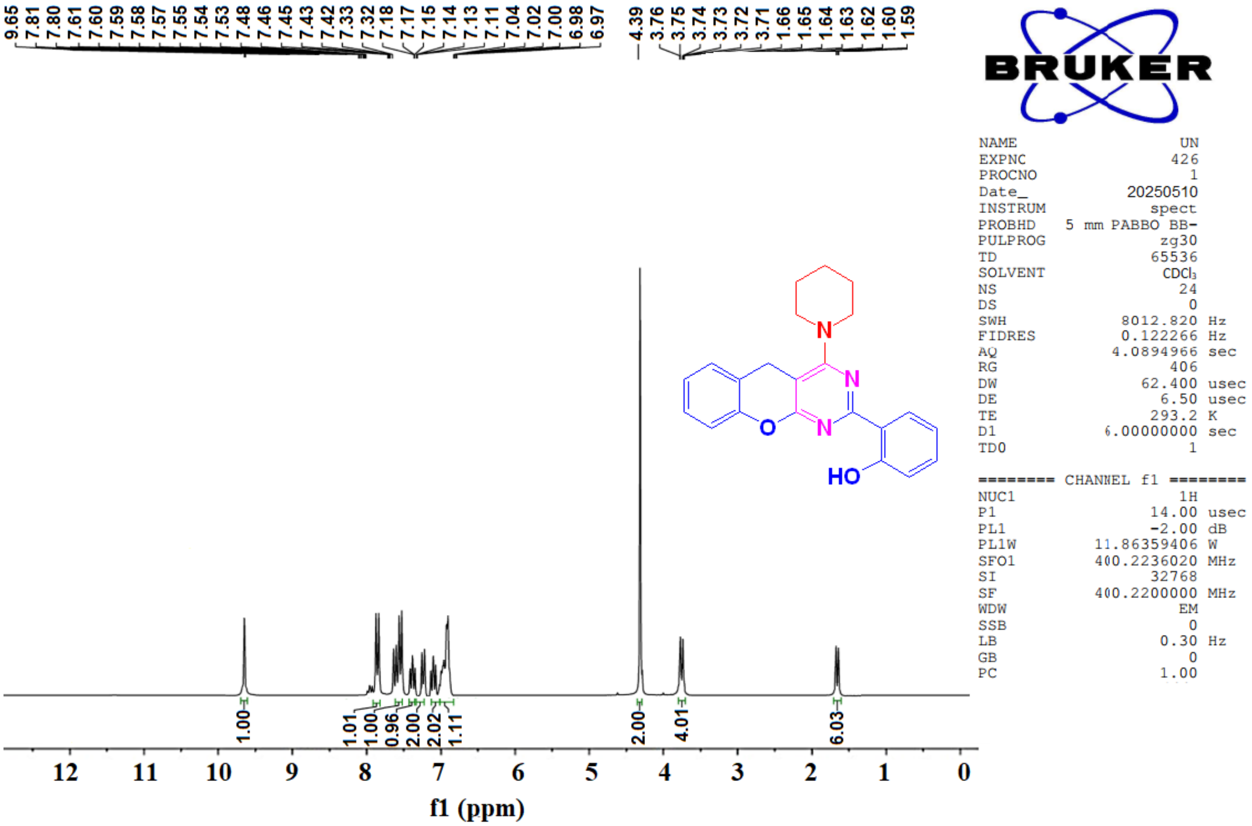
9.65
7.81
7.80
7.61
7.60
7.59
7.58
7.57
7.55
7.54
7.53
7.48
7.46
7.45
7.43
7.42
7.33
7.32
7.18
7.17
7.15
7.14
7.13
7.11
7.04
7.02
7.00
6.98
6.97
4.39
3.76
3.75
3.74
3.73
3.72
3.71
1.66
1.65
1.64
1.63
1.62
1.60
1.59
1.00
1.01
1.00
0.96
2.00
2.02
1.11
2.00
4.01
6.03
12 11 10 9 8 7 6 5 4 3 2 1 0
f1 (ppm)
N
N
N
O
HO
BRUKER
NAME UN
EXPNO 426
PROCNO 1
Date_ 20250510
INSTRUM spect
PROBHD 5 mm PABBO BB-
PULPROG zg30
TD 65536
SOLVENT CDCl3
NS 24
DS 0
SWH 8012.820 Hz
FIDRES 0.122266 Hz
AQ 4.0894966 sec
RG 406
DW 62.400 usec
DE 6.50 usec
TE 293.2 K
D1 6.00000000 sec
TD0 1
======== CHANNEL f1 ========
NUC1 1H
P1 14.00 usec
PL1 -2.00 dB
PL1W 11.86359406 W
SFO1 400.2236020 MHz
SI 32768
SF 400.2200000 MHz
WDW EM
SSB 0
LB 0.30 Hz
GB 0
PC 1.00

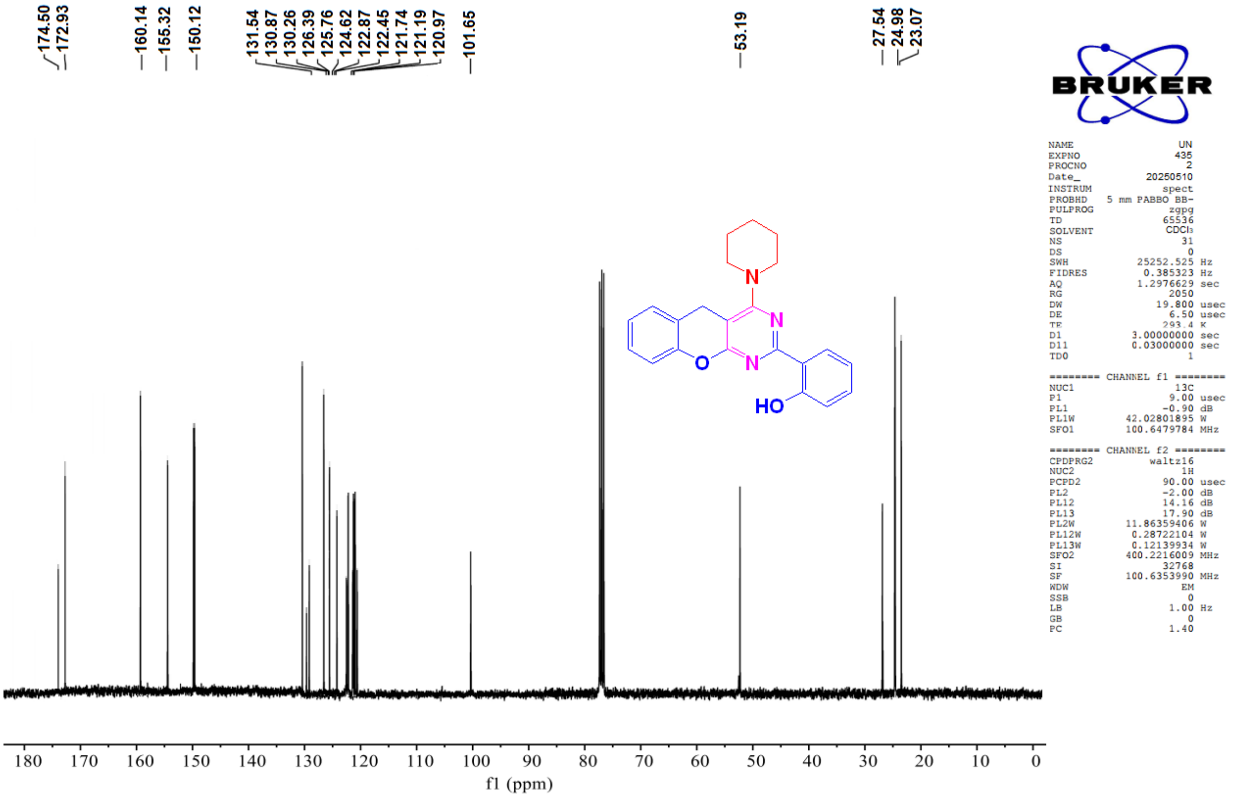
174.50
172.93
160.14
155.32
150.12
131.54
130.87
130.26
126.39
125.76
124.62
122.87
122.45
121.74
121.19
120.97
101.65
53.19
27.54
24.98
23.07
180 170 160 150 140 130 120 110 100 90 80 70 60 50 40 30 20 10 0
f1 (ppm)
N
N
N
O
HO
BRUKER
NAME UN
EXPNO 435
PROCNO 2
Date_ 20250510
INSTRUM spect
PROBHD 5 mm PABBO BB-
PULPROG zgpg
TD 65536
SOLVENT CDCl3
NS 31
DS 0
SWH 25252.525 Hz
FIDRES 0.385323 Hz
AQ 1.2976629 sec
RG 2050
DW 19.800 usec
DE 6.50 usec
D1 3.00000000 sec
D11 0.03000000 sec
TD0 1
======== CHANNEL f1 ========
NUC1 13C
P1 9.00 usec
PL1 -0.90 dB
PL1W 42.02801895 W
SFO1 100.6479784 MHz
======== CHANNEL f2 ========
CPDPRG2 waltz16
NUC2 1H
PCPD2 90.00 usec
PL2 -2.00 dB
PL12 14.16 dB
PL13 17.90 dB
PL2W 11.86359406 W
PL12W 0.28722104 W
PL13W 0.12139934 W
SFO2 400.2216009 MHz
SI 32768
SF 100.6353990 MHz
WDW EM
SSB 0
LB 1.00 Hz
GB 0
PC 1.40

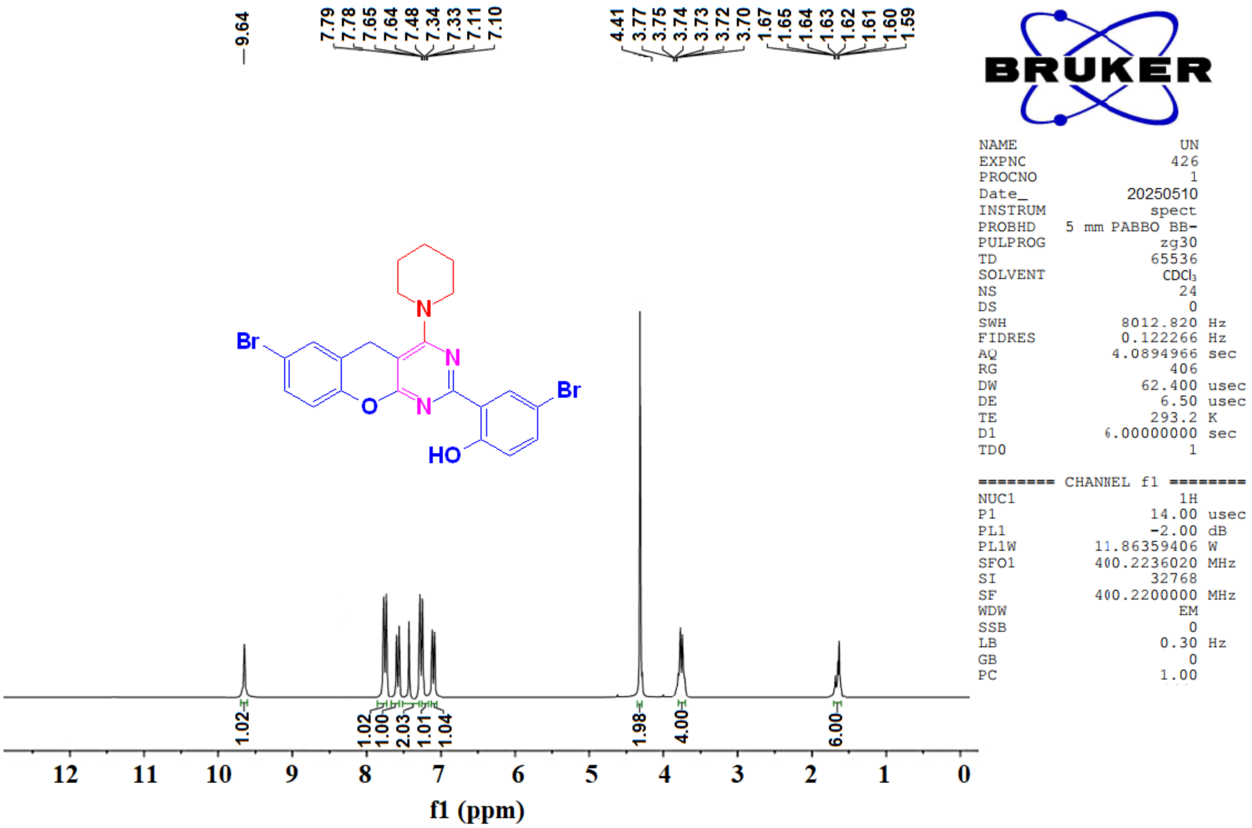

9.64
7.79 7.78 7.65 7.64 7.48 7.34 7.33 7.11 7.10
4.41 3.77 3.75 3.74 3.73 3.72 3.70 1.67 1.65 1.64 1.63 1.62 1.61 1.60 1.59
Br
N
N
N
O
Br
HO
1.02
1.02 1.00 2.03 1.01 1.04
1.98
4.00
6.00
12 11 10 9 8 7 6 5 4 3 2 1 0
f1 (ppm)
NAME UN
EXPNC 426
PROCNO 1
Date_ 20250510
INSTRUM spect
PROBHD 5 mm PABBO BB-
PULPROG zg30
TD 65536
SOLVENT CDCl3
NS 24
DS 0
SWH 8012.820 Hz
FIDRES 0.122266 Hz
AQ 4.0894966 sec
RG 406
DW 62.400 usec
DE 6.50 usec
TE 293.2 K
D1 6.00000000 sec
TD0 1
======== CHANNEL f1 ========
NUC1 1H
P1 14.00 usec
PL1 -2.00 dB
PL1W 11.86359406 W
SFO1 400.2236020 MHz
SI 32768
SF 400.2200000 MHz
WDW EM
SSB 0
LB 0.30 Hz
GB 0
PC 1.00

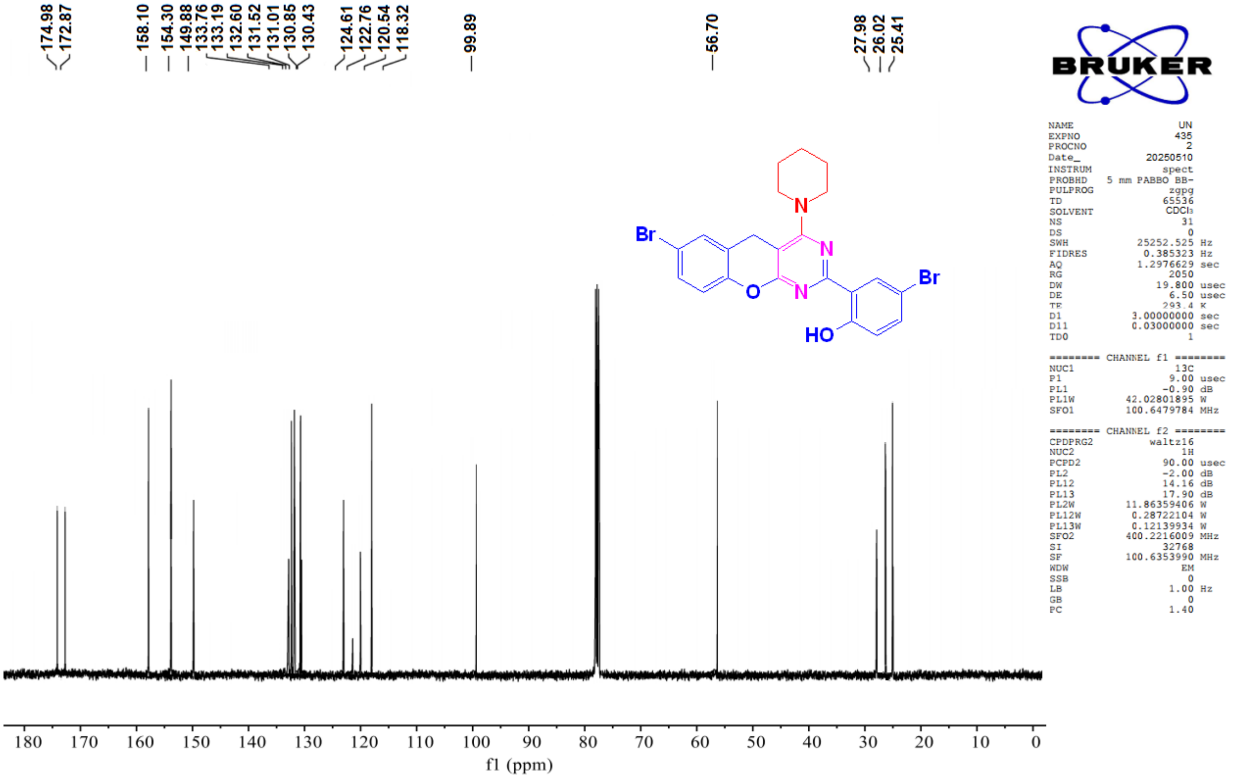

174.98 172.87
158.10 154.30 149.88 133.76 133.19 132.60 131.52 131.01 130.85 130.43
124.61 122.76 120.54 118.32
99.89
56.70
27.98 26.02 25.41
Br
N
N
N
O
Br
HO
180 170 160 150 140 130 120 110 100 90 80 70 60 50 40 30 20 10 0
f1 (ppm)
NAME UN
EXPNO 435
PROCNO 2
Date_ 20250510
INSTRUM spect
PROBHD 5 mm PABBO BB-
PULPROG zgpg
TD 65536
SOLVENT CDCl3
NS 31
DS 0
SWH 25252.525 Hz
FIDRES 0.385323 Hz
AQ 1.2976629 sec
RG 2050
DW 19.800 usec
DE 6.50 usec
TE 293.4 K
D1 3.00000000 sec
D11 0.03000000 sec
TD0 1
======== CHANNEL f1 ========
NUC1 13C
P1 9.00 usec
PL1 -0.90 dB
PL1W 42.02801895 W
SFO1 100.6479784 MHz
======== CHANNEL f2 ========
CPDPRG2 waltz16
NUC2 1H
PCPD2 90.00 usec
PL2 -2.00 dB
PL12 14.16 dB
PL13 17.90 dB
PL2W 11.86359406 W
PL12W 0.28722104 W
PL13W 0.12139934 W
SFO2 400.2216009 MHz
SI 32768
SF 100.6353990 MHz
WDW EM
SSB 0
LB 1.00 Hz
GB 0
PC 1.40

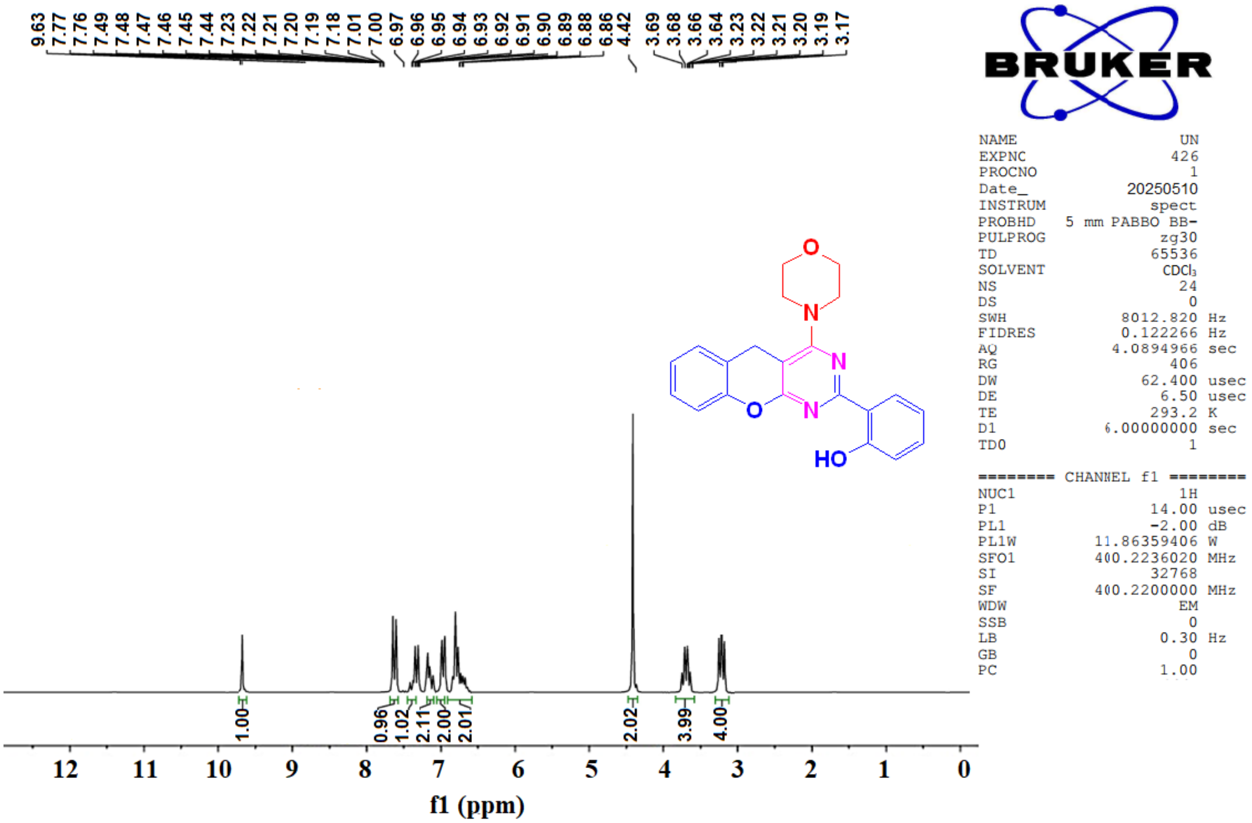
9.63
7.77
7.76
7.49
7.48
7.47
7.46
7.45
7.44
7.23
7.22
7.21
7.20
7.19
7.18
7.01
7.00
6.97
6.96
6.95
6.94
6.93
6.92
6.91
6.90
6.89
6.88
6.86
4.42
3.69
3.68
3.66
3.64
3.23
3.22
3.21
3.20
3.19
3.17
BRUKER
NAME UN
EXPNC 426
PROCNO 1
Date_ 20250510
INSTRUM spect
PROBHD 5 mm PABBO BB-
PULPROG zg30
TD 65536
SOLVENT CDCl3
NS 24
DS 0
SWH 8012.820 Hz
FIDRES 0.122266 Hz
AQ 4.0894966 sec
RG 406
DW 62.400 usec
DE 6.50 usec
TE 293.2 K
D1 6.00000000 sec
TD0 1
======== CHANNEL f1 ========
NUC1 1H
P1 14.00 usec
PL1 -2.00 dB
PL1W 11.86359406 W
SFO1 400.2236020 MHz
SI 32768
SF 400.2200000 MHz
WDW EM
SSB 0
LB 0.30 Hz
GB 0
PC 1.00
O
N
N
N
O
HO
1.00
0.96
1.02
2.11
2.00
2.01
2.02
3.99
4.00
12 11 10 9 8 7 6 5 4 3 2 1 0
f1 (ppm)

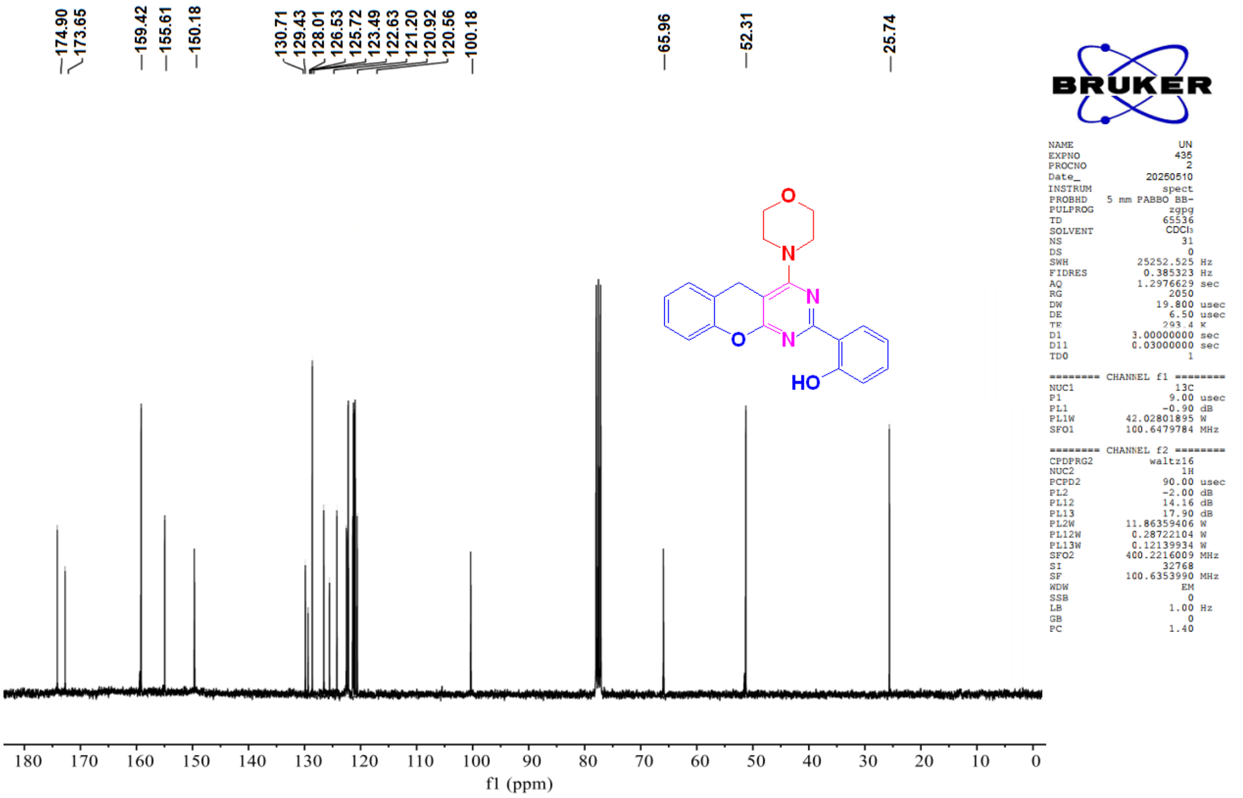
174.90
173.65
159.42
155.61
150.18
130.71
129.43
128.01
126.53
125.72
123.49
122.63
121.20
120.92
120.56
100.18
65.96
52.31
25.74
BRUKER
NAME UN
EXPNO 435
PROCNO 2
Date_ 20250510
INSTRUM spect
PROBHD 5 mm PABBO BB-
PULPROG zgpg
TD 65536
SOLVENT CDCl3
NS 31
DS 0
SWH 25252.525 Hz
FIDRES 0.385323 Hz
AQ 1.2976629 sec
RG 2050
DW 19.800 usec
DE 6.50 usec
D1 3.00000000 sec
D11 0.03000000 sec
TD0 1
======= CHANNEL f1 =======
NUC1 13C
P1 9.00 usec
PL1 -0.90 dB
PL1W 42.02801895 W
SFO1 100.6479784 MHz
======= CHANNEL f2 =======
CPDPRG2 waltz16
NUC2 1H
PCPD2 90.00 usec
PL2 -2.00 dB
PL12 14.16 dB
PL13 17.90 dB
PL2W 11.86359406 W
PL12W 0.28722104 W
PL13W 0.12139934 W
SFO2 400.2216009 MHz
SI 32768
SF 100.6353990 MHz
WDW EM
SSB 0
LB 1.00 Hz
GB 0
PC 1.40
O
N
N
N
O
HO
180 170 160 150 140 130 120 110 100 90 80 70 60 50 40 30 20 10 0
f1 (ppm)

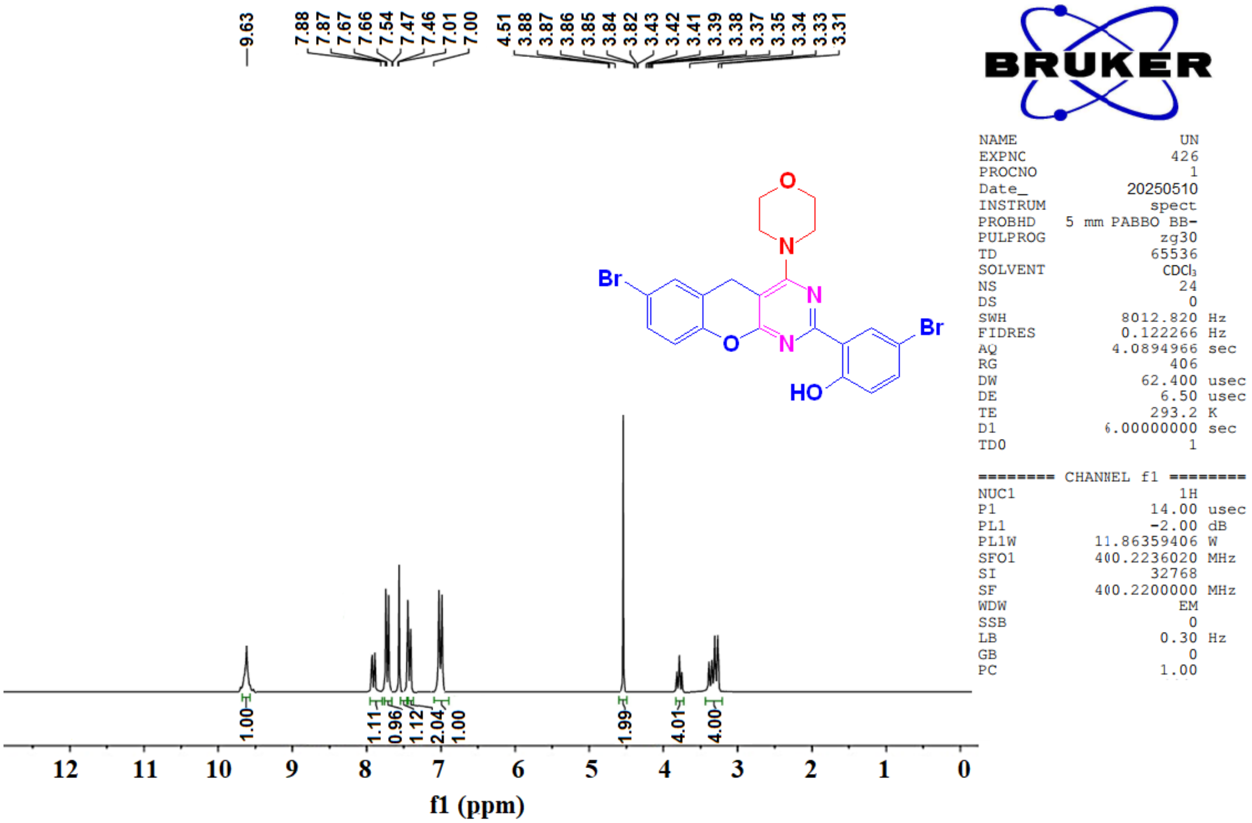
9.63
7.88
7.87
7.67
7.66
7.54
7.47
7.46
7.01
7.00
4.51
3.88
3.87
3.86
3.85
3.84
3.82
3.43
3.42
3.41
3.39
3.38
3.37
3.35
3.34
3.33
3.31
BRUKER
NAME UN
EXPNO 426
PROCNO 1
Date_ 20250510
INSTRUM spect
PROBHD 5 mm PABBO BB-
PULPROG zg30
TD 65536
SOLVENT CDCl3
NS 24
DS 0
SWH 8012.820 Hz
FIDRES 0.122266 Hz
AQ 4.0894966 sec
RG 406
DW 62.400 usec
DE 6.50 usec
TE 293.2 K
D1 6.00000000 sec
TD0 1
======== CHANNEL f1 ========
NUC1 1H
P1 14.00 usec
PL1 -2.00 dB
PL1W 11.86359406 W
SFO1 400.2236020 MHz
SI 32768
SF 400.2200000 MHz
WDW EM
SSB 0
LB 0.30 Hz
GB 0
PC 1.00
O
N
Br
N
N
O
Br
HO
1.00
1.11
0.96
1.12
2.04
1.00
1.99
4.01
4.00
12
11
10
9
8
7
6
5
4
3
2
1
0
f1 (ppm)

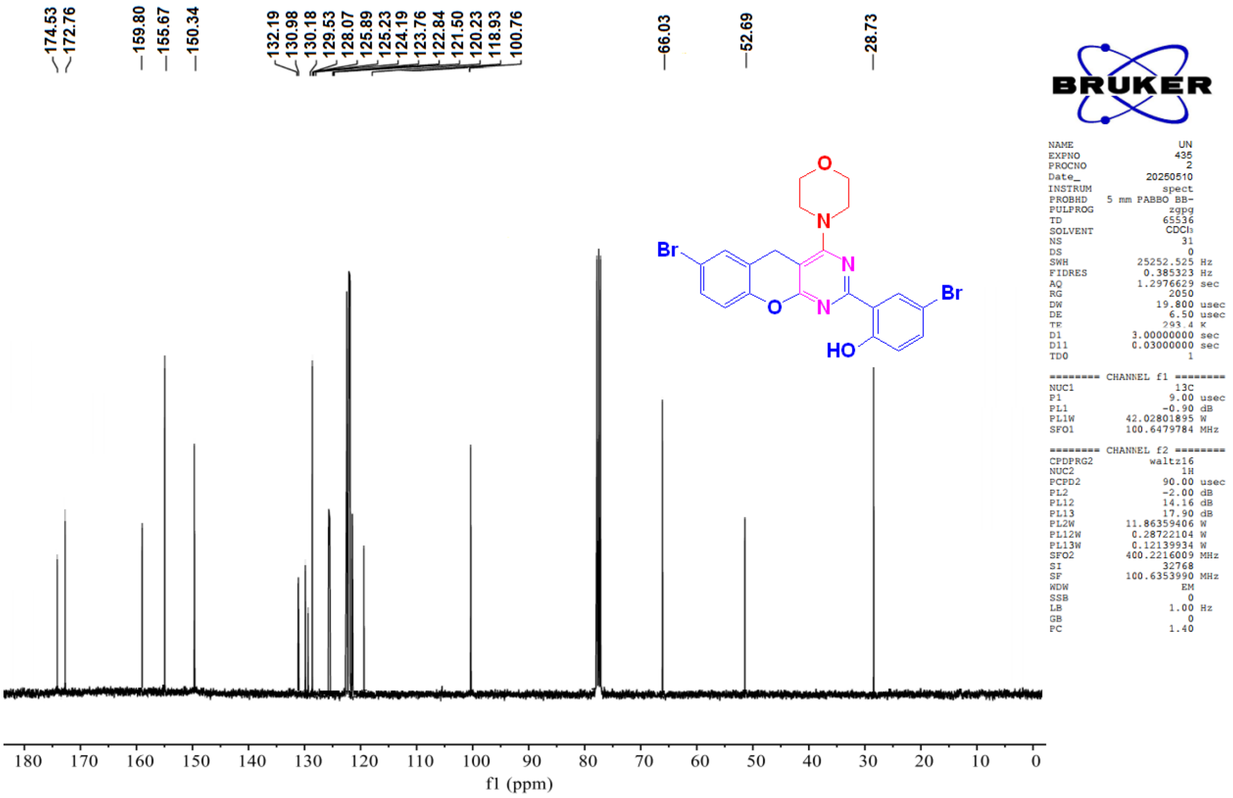
174.53
172.76
159.80
155.67
150.34
132.19
130.98
130.18
129.53
128.07
125.89
125.23
124.19
123.76
122.84
121.50
120.23
118.93
100.76
66.03
52.69
28.73
BRUKER
NAME UN
EXPNO 435
PROCNO 2
Date_ 20250510
INSTRUM spect
PROBHD 5 mm PABBO BB-
PULPROG zgpg
TD 65536
SOLVENT CDCl3
NS 31
DS 0
SWH 25252.525 Hz
FIDRES 0.385323 Hz
AQ 1.2976629 sec
RG 2050
DW 19.800 usec
DE 6.50 usec
D1 3.00000000 sec
D11 0.03000000 sec
TD0 1
======== CHANNEL f1 ========
NUC1 13C
P1 9.00 usec
PL1 -0.90 dB
PL1W 42.02801895 W
SFO1 100.6479784 MHz
======== CHANNEL f2 ========
CPDPRG2 waltz16
NUC2 1H
PCPD2 90.00 usec
PL2 -2.00 dB
PL12 14.16 dB
PL13 17.90 dB
PL2W 11.86359406 W
PL12W 0.28722104 W
PL13W 0.12139934 W
SFO2 400.2216009 MHz
SI 32768
SF 100.6353990 MHz
WDW EM
SSB 0
LB 1.00 Hz
GB 0
PC 1.40
O
N
Br
N
N
O
Br
HO
180
170
160
150
140
130
120
110
100
90
80
70
60
50
40
30
20
10
0
f1 (ppm)

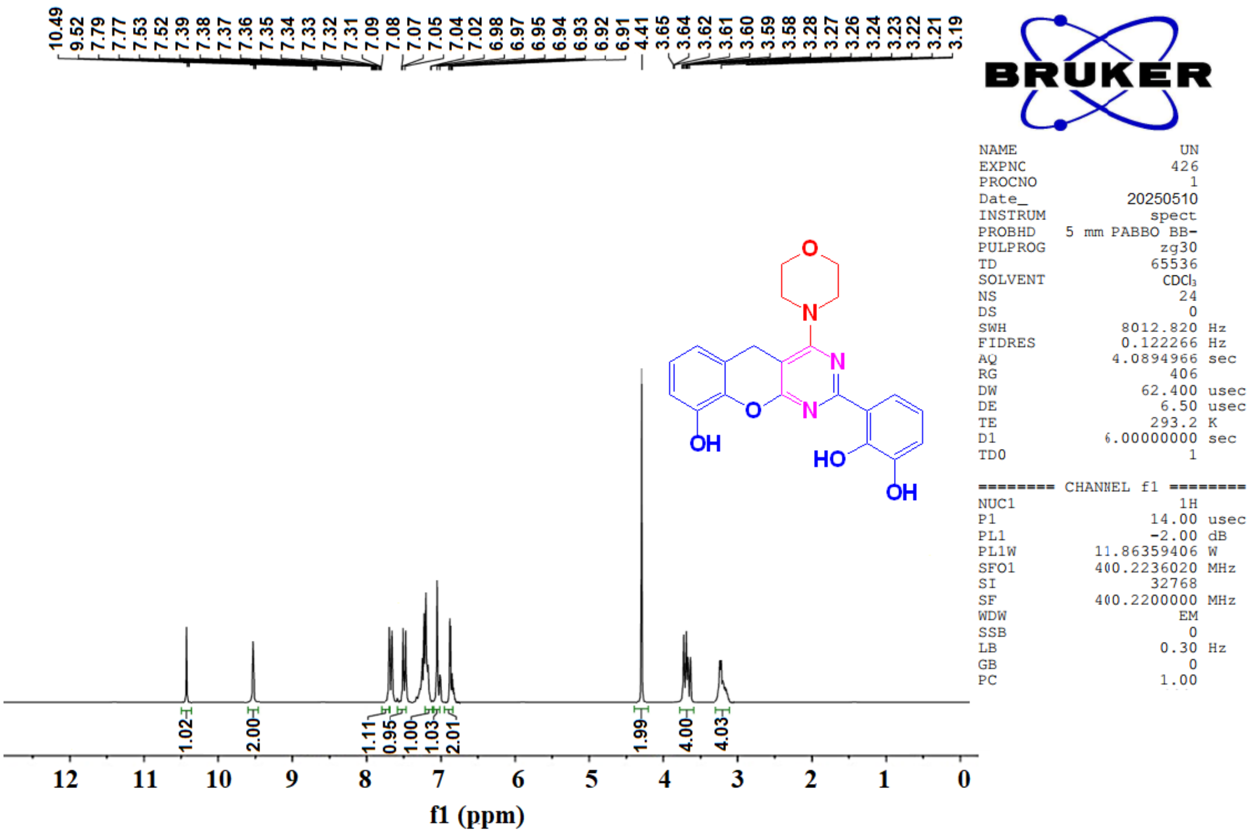
10.49
9.52
7.79
7.77
7.53
7.52
7.39
7.38
7.37
7.36
7.35
7.34
7.33
7.32
7.31
7.09
7.08
7.07
7.05
7.04
7.02
6.98
6.97
6.95
6.94
6.93
6.92
6.91
4.41
3.65
3.64
3.62
3.61
3.60
3.59
3.58
3.28
3.27
3.26
3.24
3.23
3.22
3.21
3.19
BRUKER
NAME UN
EXPNC 426
PROCNO 1
Date_ 20250510
INSTRUM spect
PROBHD 5 mm PABBO BB-
PULPROG zg30
TD 65536
SOLVENT CDCl3
NS 24
DS 0
SWH 8012.820 Hz
FIDRES 0.122266 Hz
AQ 4.0894966 sec
RG 406
DW 62.400 usec
DE 6.50 usec
TE 293.2 K
D1 6.00000000 sec
TD0 1
======== CHANNEL f1 ========
NUC1 1H
P1 14.00 usec
PL1 -2.00 dB
PL1W 11.86359406 W
SFO1 400.2236020 MHz
SI 32768
SF 400.2200000 MHz
WDW EM
SSB 0
LB 0.30 Hz
GB 0
PC 1.00
O
N
N
N
O
OH
HO
OH
1.02
2.00
1.11
0.95
1.00
1.03
2.01
1.99
4.00
4.03
12
11
10
9
8
7
6
5
4
3
2
1
0
f1 (ppm)

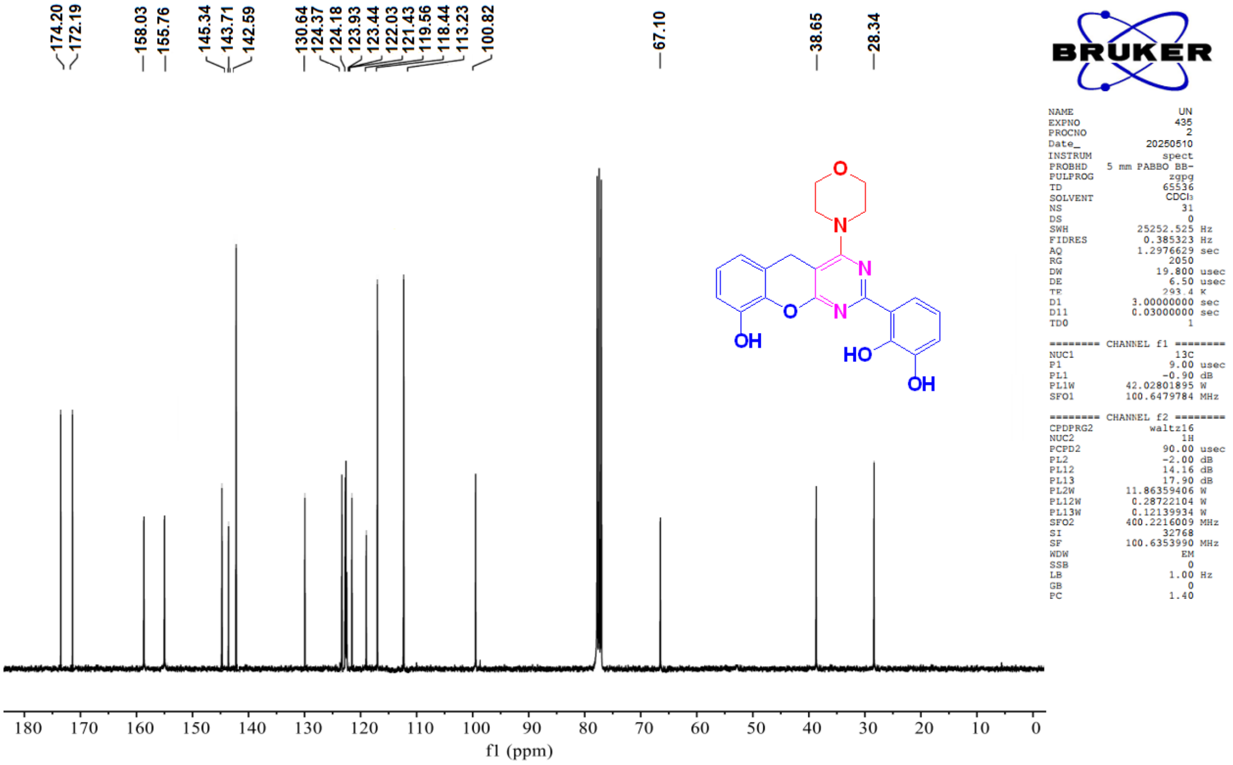
174.20
172.19
158.03
155.76
145.34
143.71
142.59
130.64
124.37
124.18
123.93
123.44
122.03
121.43
119.56
118.44
113.23
100.82
67.10
38.65
28.34
BRUKER
O
N
N
N
O
OH
HO
OH
180
170
160
150
140
130
120
110
100
90
80
70
60
50
40
30
20
10
0
f1 (ppm)

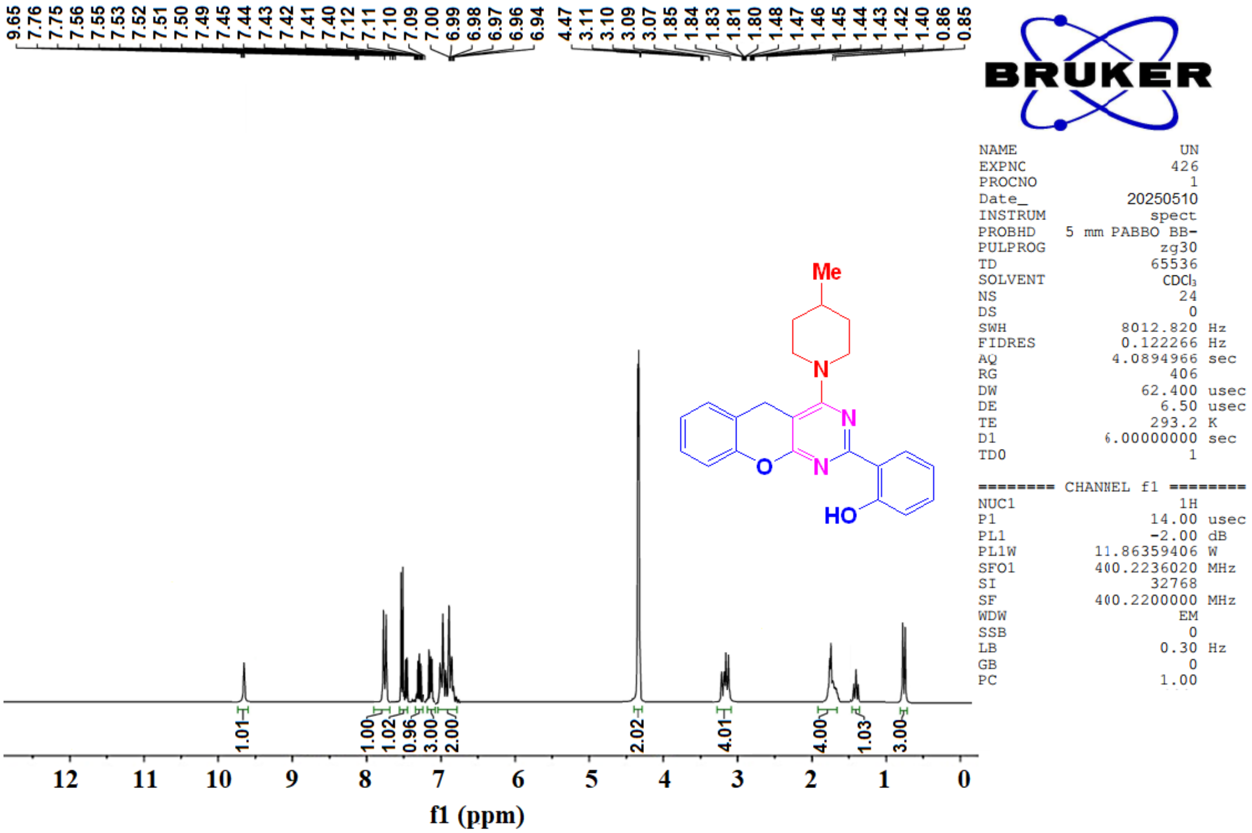

9.65 7.76 7.75 7.56 7.55 7.53 7.52 7.51 7.50 7.49 7.45 7.44 7.43 7.42 7.41 7.40 7.12 7.11 7.10 7.09 7.00 6.99 6.98 6.97 6.96 6.94 4.47 3.11 3.10 3.09 3.07 1.85 1.84 1.83 1.81 1.80 1.48 1.47 1.46 1.45 1.44 1.43 1.42 1.40 0.86 0.85
BRUKER
NAME UN
EXPNC 426
PROCNO 1
Date_ 20250510
INSTRUM spect
PROBHD 5 mm PABBO BB-
PULPROG zg30
TD 65536
SOLVENT CDCl3
NS 24
DS 0
SWH 8012.820 Hz
FIDRES 0.122266 Hz
AQ 4.0894966 sec
RG 406
DW 62.400 usec
DE 6.50 usec
TE 293.2 K
D1 6.00000000 sec
TD0 1
======== CHANNEL f1 ========
NUC1 1H
P1 14.00 usec
PL1 -2.00 dB
PL1W 11.86359406 W
SFO1 400.2236020 MHz
SI 32768
SF 400.2200000 MHz
WDW EM
SSB 0
LB 0.30 Hz
GB 0
PC 1.00
Me
N
N
N
O
HO
1.01 1.00 1.02 0.96 3.00 3.00 2.00 2.02 4.01 4.00 1.03 3.00
12 11 10 9 8 7 6 5 4 3 2 1 0
f1 (ppm)

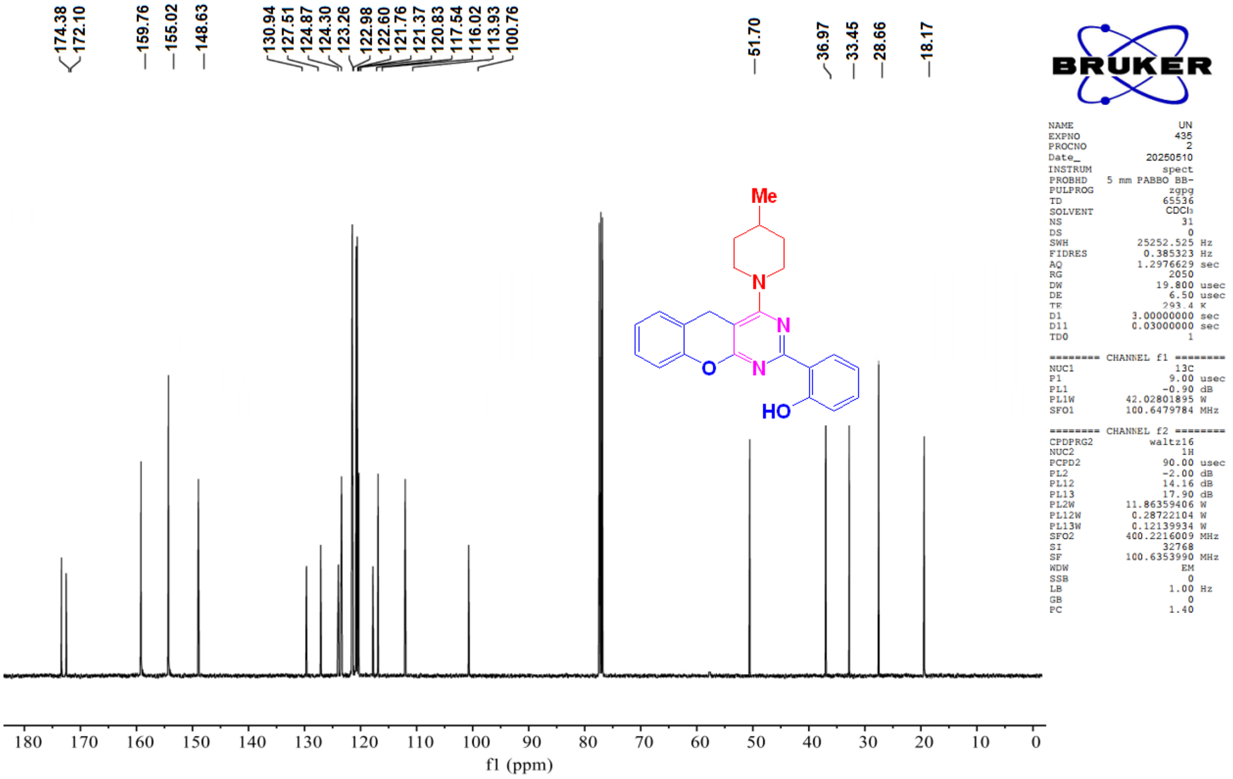

174.38 172.10 159.76 155.02 148.63 130.94 127.51 124.87 124.30 123.26 122.98 122.60 121.76 121.37 120.83 117.54 116.02 113.93 100.76 51.70 36.97 33.45 28.66 18.17
BRUKER
NAME UN
EXPNO 435
PROCNO 2
Date_ 20250510
INSTRUM spect
PROBHD 5 mm PABBO BB-
PULPROG zgpg
TD 65536
SOLVENT CDCl3
NS 31
DS 0
SWH 25252.525 Hz
FIDRES 0.385323 Hz
AQ 1.2976629 sec
RG 2050
DW 19.800 usec
DE 6.50 usec
TE 293.4 K
D1 3.00000000 sec
D11 0.03000000 sec
TD0 1
======== CHANNEL f1 ========
NUC1 13C
P1 9.00 usec
PL1 -0.90 dB
PL1W 42.02801895 W
SFO1 100.6479784 MHz
======== CHANNEL f2 ========
CPDPRG2 waltz16
NUC2 1H
PCPD2 90.00 usec
PL2 -2.00 dB
PL12 14.16 dB
PL13 17.90 dB
PL2W 11.86359406 W
PL12W 0.28722104 W
PL13W 0.12139934 W
SFO2 400.2216009 MHz
SI 32768
SF 100.6353990 MHz
WDW EM
SSB 0
LB 1.00 Hz
GB 0
PC 1.40
Me
N
N
N
O
HO
180 170 160 150 140 130 120 110 100 90 80 70 60 50 40 30 20 10 0
f1 (ppm)

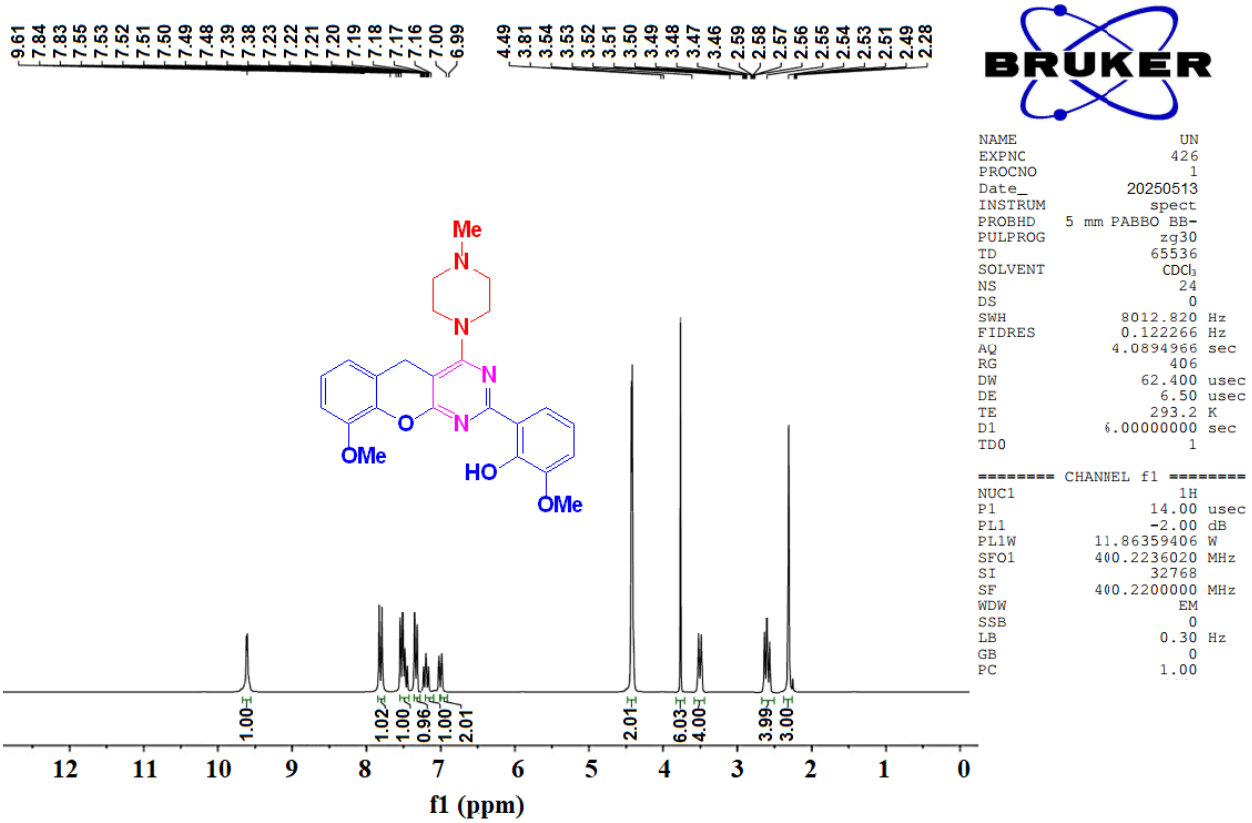
9.61
7.84
7.83
7.55
7.53
7.52
7.51
7.50
7.49
7.48
7.39
7.38
7.23
7.22
7.21
7.20
7.19
7.18
7.17
7.16
7.00
6.99
4.49
3.81
3.54
3.53
3.52
3.51
3.50
3.49
3.48
3.47
3.46
2.59
2.58
2.57
2.56
2.55
2.54
2.53
2.51
2.49
2.28
Me
N
N
N
N
O
OMe
HO
OMe
1.00
1.02
1.00
0.96
1.00
2.01
2.01
6.03
4.00
3.99
3.00
12
11
10
9
8
7
6
5
4
3
2
1
0
f1 (ppm)
NAME UN
EXPNC 426
PROCNO 1
Date_ 20250513
INSTRUM spect
PROBHD 5 mm PABBO BB-
PULPROG zg30
TD 65536
SOLVENT CDCl3
NS 24
DS 0
SWH 8012.820 Hz
FIDRES 0.122266 Hz
AQ 4.0894966 sec
RG 406
DW 62.400 usec
DE 6.50 usec
TE 293.2 K
D1 6.00000000 sec
TD0 1
======== CHANNEL f1 ========
NUC1 1H
P1 14.00 usec
PL1 -2.00 dB
PL1W 11.86359406 W
SFO1 400.2236020 MHz
SI 32768
SF 400.2200000 MHz
WDW EM
SSB 0
LB 0.30 Hz
GB 0
PC 1.00

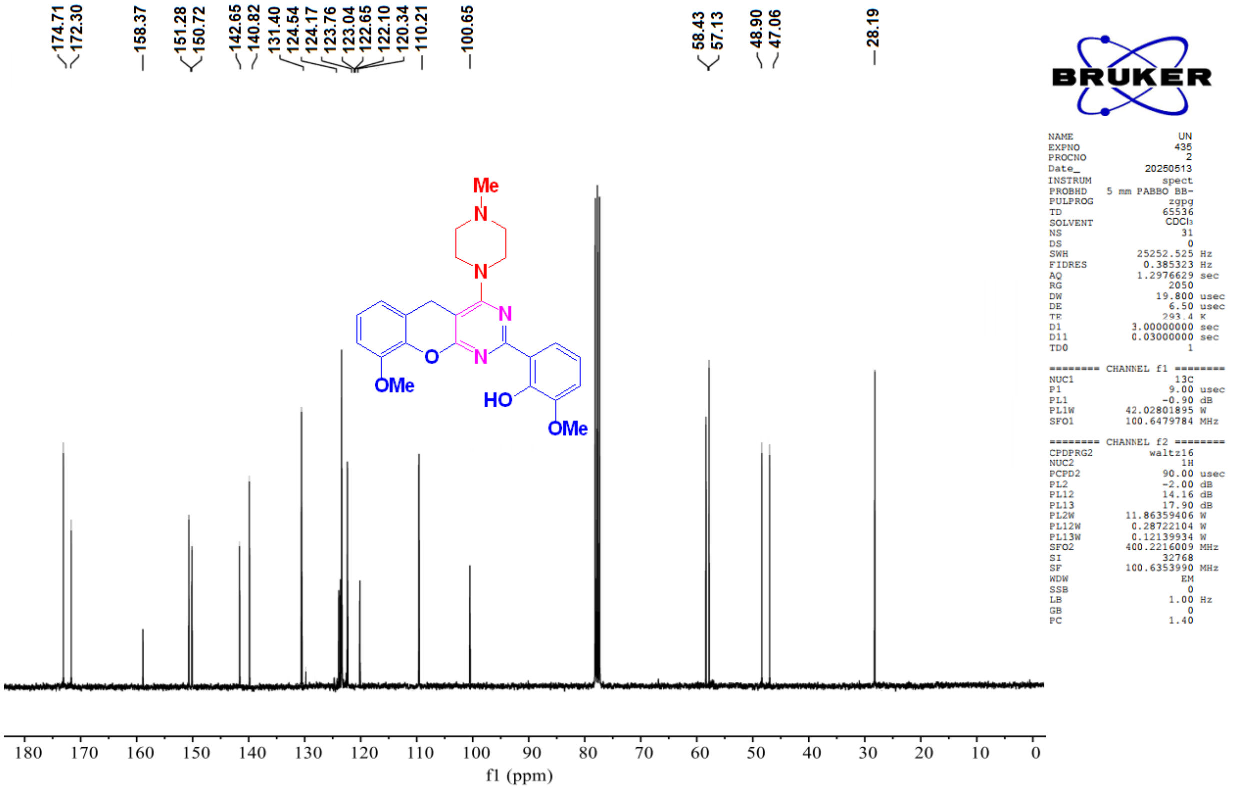
174.71
172.30
158.37
151.28
150.72
142.65
140.82
131.40
124.54
124.17
123.76
123.04
122.65
122.10
120.34
110.21
100.65
58.43
57.13
48.90
47.06
28.19
Me
N
N
N
N
O
OMe
HO
OMe
180
170
160
150
140
130
120
110
100
90
80
70
60
50
40
30
20
10
0
f1 (ppm)
NAME UN
EXPNO 435
PROCNO 2
Date_ 20250513
INSTRUM spect
PROBHD 5 mm PABBO BB-
PULPROG zgpg
TD 65536
SOLVENT CDCl3
NS 31
DS 0
SWH 25252.525 Hz
FIDRES 0.385323 Hz
AQ 1.2976629 sec
RG 2050
DW 19.800 usec
DE 6.50 usec
TE 293.4 K
D1 3.00000000 sec
D11 0.03000000 sec
TD0 1
======== CHANNEL f1 ========
NUC1 13C
P1 9.00 usec
PL1 -0.90 dB
PL1W 42.02801895 W
SFO1 100.6479784 MHz
======== CHANNEL f2 ========
CPDPRG2 waltz16
NUC2 1H
PCPD2 90.00 usec
PL2 -2.00 dB
PL12 14.16 dB
PL13 17.90 dB
PL2W 11.86359406 W
PL12W 0.28722104 W
PL13W 0.12139934 W
SFO2 400.2216009 MHz
SI 32768
SF 100.6353990 MHz
WDW EM
SSB 0
LB 1.00 Hz
GB 0
PC 1.40

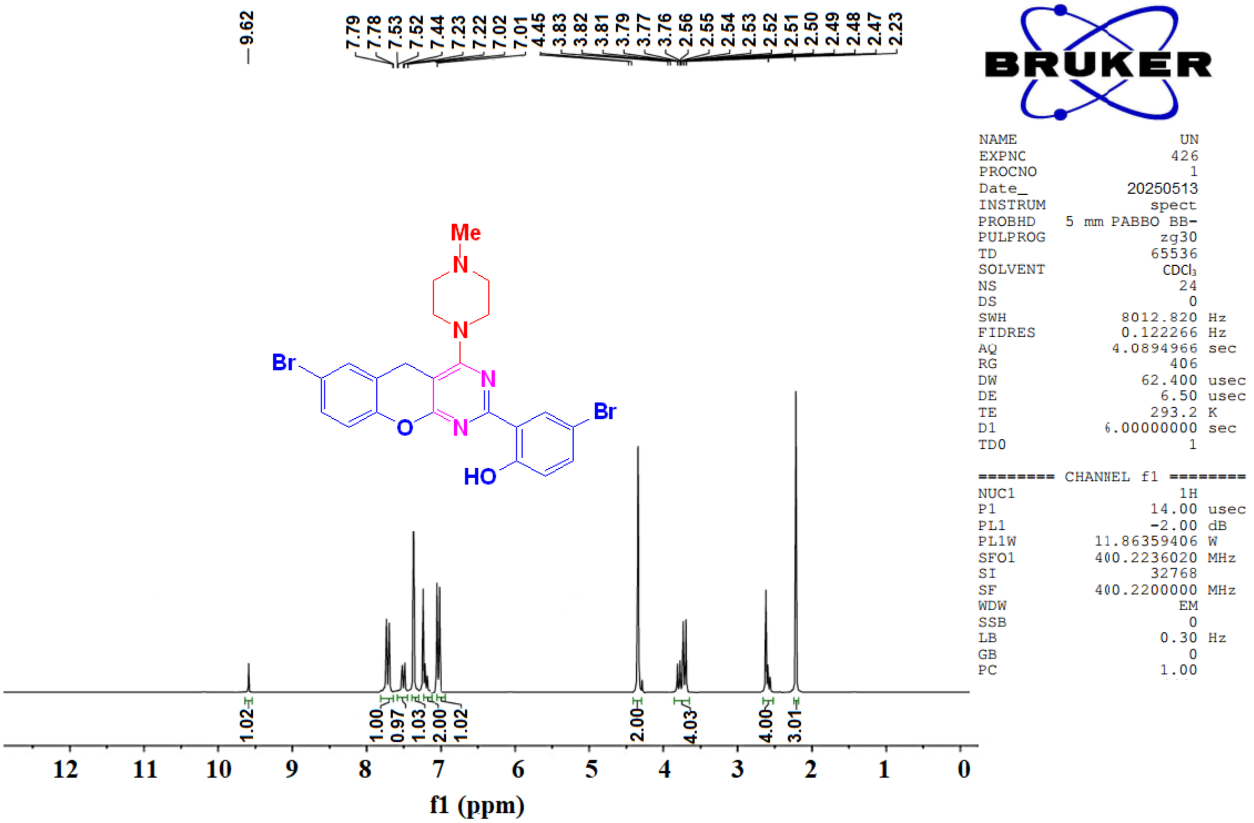

9.62
7.79 7.78 7.53 7.52 7.44 7.23 7.22 7.02 7.01 4.45 3.83 3.82 3.81 3.79 3.77 3.76 2.56 2.55 2.54 2.53 2.52 2.51 2.50 2.49 2.48 2.47 2.23
BRUKER
NAME UN
EXPNC 426
PROCNO 1
Date_ 20250513
INSTRUM spect
PROBHD 5 mm PABBO BB-
PULPROG zg30
TD 65536
SOLVENT CDCl3
NS 24
DS 0
SWH 8012.820 Hz
FIDRES 0.122266 Hz
AQ 4.0894966 sec
RG 406
DW 62.400 usec
DE 6.50 usec
TE 293.2 K
D1 6.00000000 sec
TD0 1
======== CHANNEL f1 ========
NUC1 1H
P1 14.00 usec
PL1 -2.00 dB
PL1W 11.86359406 W
SFO1 400.2236020 MHz
SI 32768
SF 400.2200000 MHz
WDW EM
SSB 0
LB 0.30 Hz
GB 0
PC 1.00
Me
N
N
Br
N
N
O
Br
HO
1.02
1.00 0.97 1.03 2.00 1.02
2.00
4.03
4.00
3.01
12 11 10 9 8 7 6 5 4 3 2 1 0
f1 (ppm)

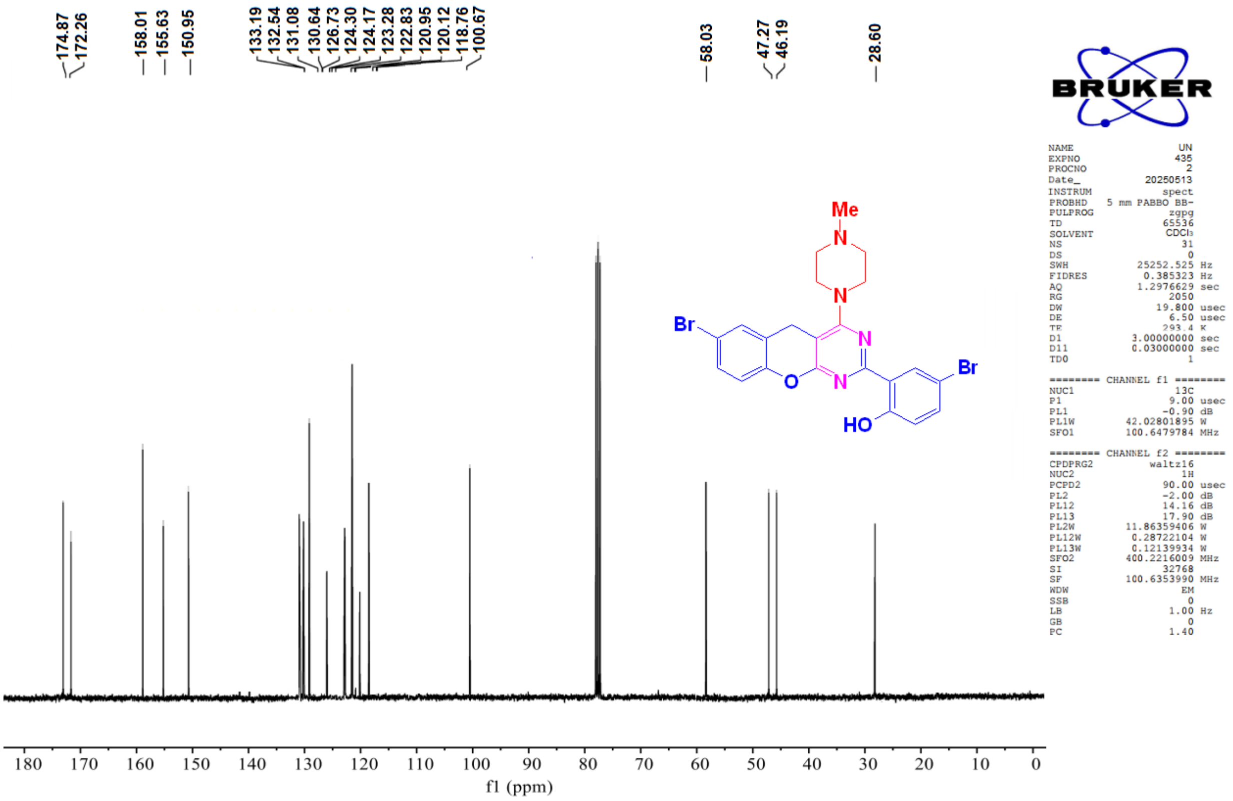

174.87 172.26
158.01 155.63 150.95
133.19 132.54 131.08 130.64 126.73 124.30 124.17 123.28 122.83 120.95 120.12 118.76 100.67
58.03
47.27 46.19
28.60
BRUKER
Me
N
N
Br
N
N
O
Br
HO
180 170 160 150 140 130 120 110 100 90 80 70 60 50 40 30 20 10 0
f1 (ppm)

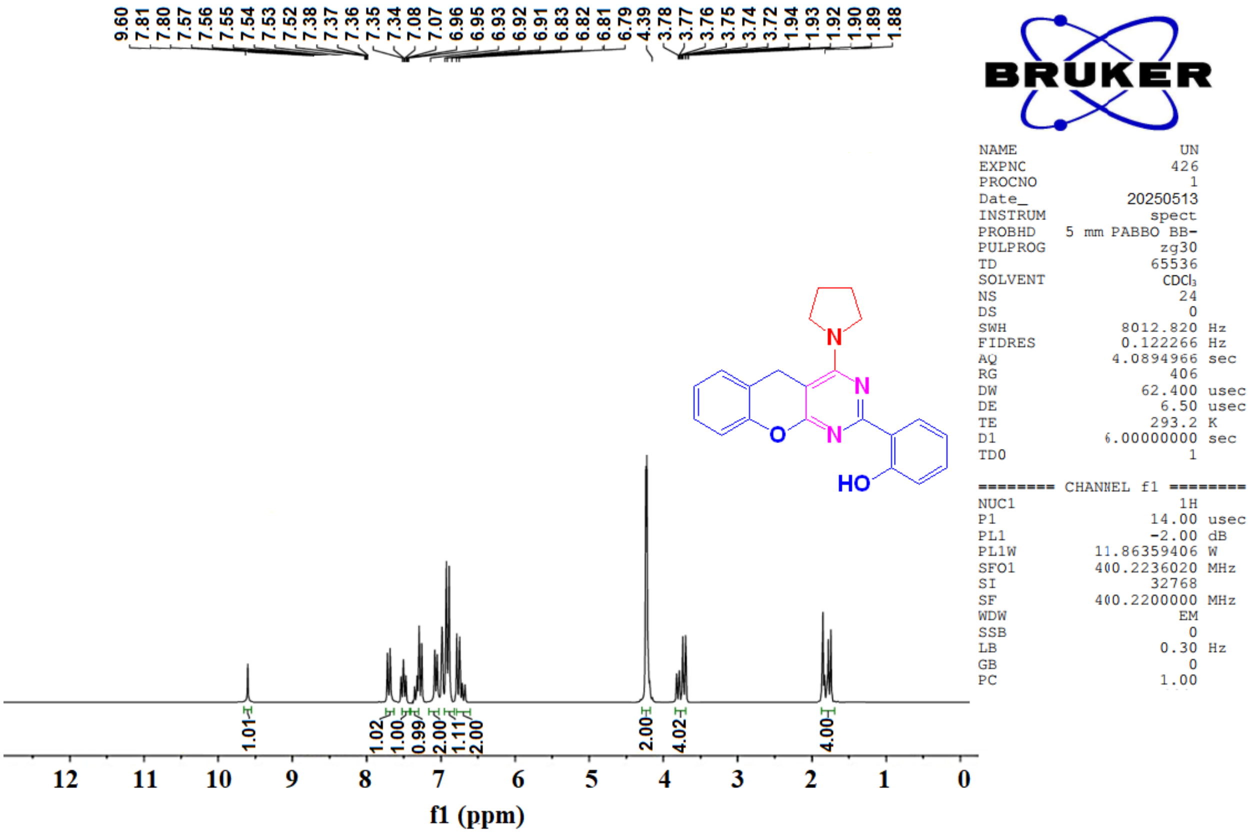
9.60 7.81 7.80 7.57 7.56 7.55 7.54 7.53 7.52 7.38 7.37 7.36 7.35 7.34 7.08 7.07 6.96 6.95 6.93 6.92 6.91 6.83 6.82 6.81 6.79 4.39 3.78 3.77 3.76 3.75 3.74 3.72 1.94 1.93 1.92 1.90 1.89 1.88
BRUKER
NAME UN
EXPNC 426
PROCNO 1
Date_ 20250513
INSTRUM spect
PROBHD 5 mm PABBO BB-
PULPROG zg30
TD 65536
SOLVENT CDCl3
NS 24
DS 0
SWH 8012.820 Hz
FIDRES 0.122266 Hz
AQ 4.0894966 sec
RG 406
DW 62.400 usec
DE 6.50 usec
TE 293.2 K
D1 6.00000000 sec
TD0 1
======== CHANNEL f1 ========
NUC1 1H
P1 14.00 usec
PL1 -2.00 dB
PL1W 11.86359406 W
SFO1 400.2236020 MHz
SI 32768
SF 400.2200000 MHz
WDW EM
SSB 0
LB 0.30 Hz
GB 0
PC 1.00
N
N
N
O
HO
1.01 1.02 1.00 0.99 2.00 1.11 2.00 2.00 4.02 4.00
12 11 10 9 8 7 6 5 4 3 2 1 0
f1 (ppm)

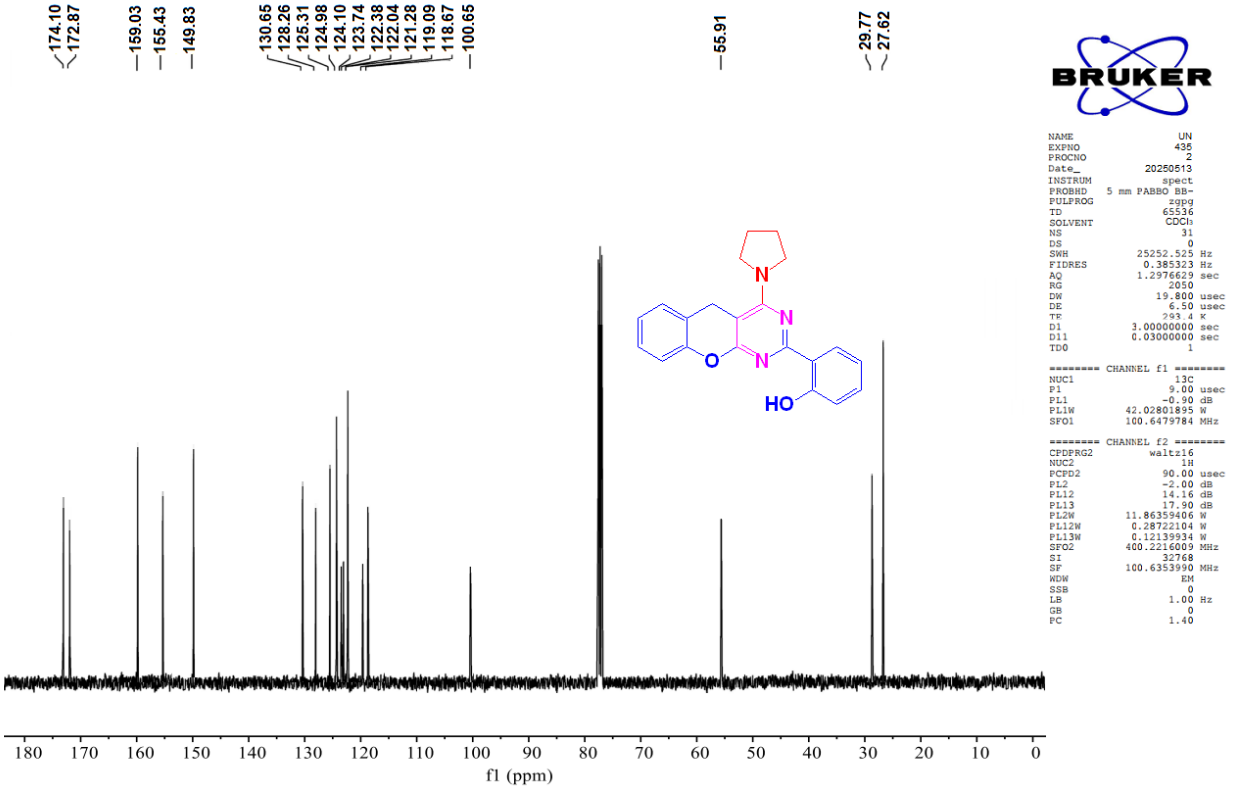
174.10 172.87 159.03 155.43 149.83 130.65 128.26 125.31 124.98 124.10 123.74 122.38 122.04 121.28 119.09 118.67 100.65 55.91 29.77 27.62
BRUKER
NAME UN
EXPNO 435
PROCNO 2
Date_ 20250513
INSTRUM spect
PROBHD 5 mm PABBO BB-
PULPROG zgpg
TD 65536
SOLVENT CDCl3
NS 31
DS 0
SWH 25252.525 Hz
FIDRES 0.385323 Hz
AQ 1.2976629 sec
RG 2050
DW 19.800 usec
DE 6.50 usec
D1 3.00000000 sec
D11 0.03000000 sec
TD0 1
======== CHANNEL f1 ========
NUC1 13C
P1 9.00 usec
PL1 -0.90 dB
PL1W 42.02801895 W
SFO1 100.6479784 MHz
======== CHANNEL f2 ========
CPDPRG2 waltz16
NUC2 1H
PCPD2 90.00 usec
PL2 -2.00 dB
PL12 14.16 dB
PL13 17.90 dB
PL2W 11.86359406 W
PL12W 0.28722104 W
PL13W 0.12139934 W
SFO2 400.2216009 MHz
SI 32768
SF 100.6353990 MHz
WDW EM
SSB 0
LB 1.00 Hz
GB 0
PC 1.40
N
N
N
O
HO
180 170 160 150 140 130 120 110 100 90 80 70 60 50 40 30 20 10 0
f1 (ppm)

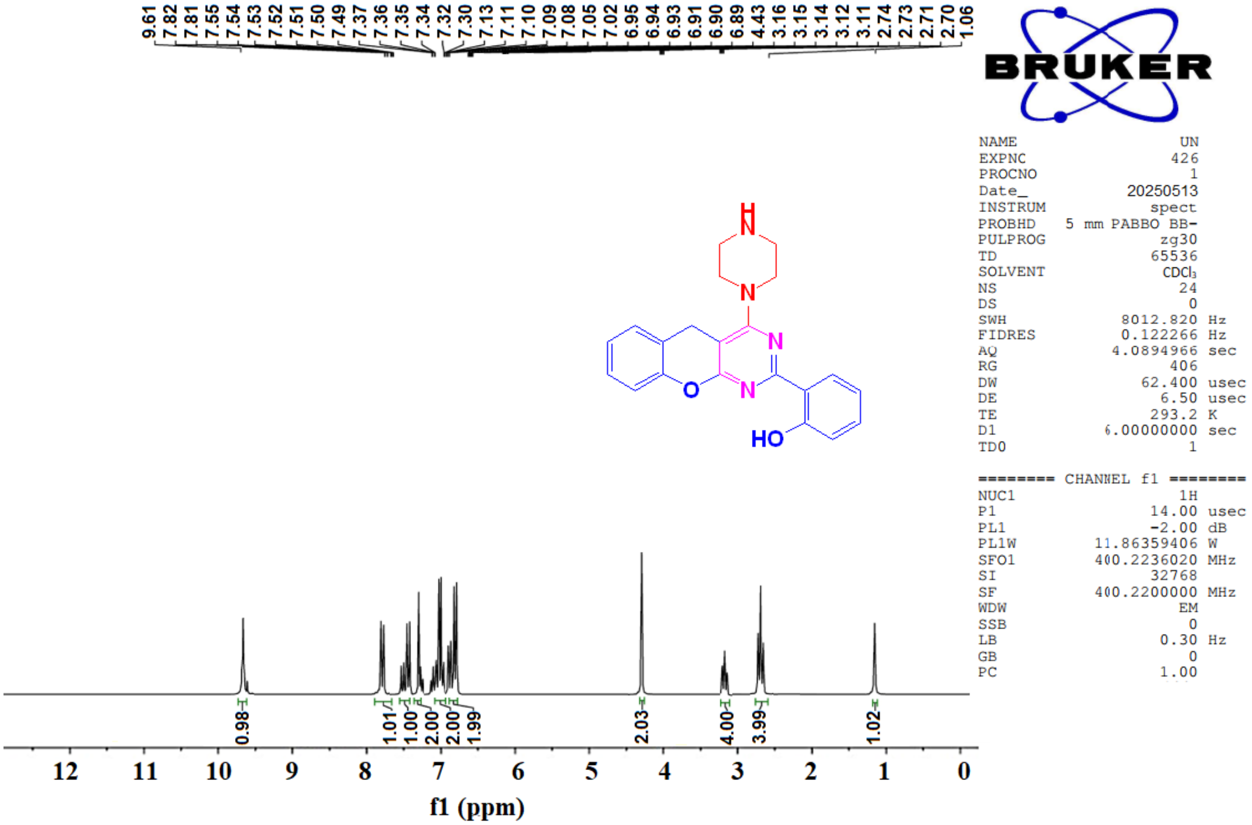
9.61 7.82 7.81 7.55 7.54 7.53 7.52 7.51 7.50 7.49 7.37 7.36 7.35 7.34 7.32 7.30 7.13 7.11 7.10 7.09 7.08 7.05 7.02 6.95 6.94 6.93 6.91 6.90 6.89 4.43 3.16 3.15 3.14 3.12 3.11 2.74 2.73 2.71 2.70 1.06
H
N
N
N
N
O
HO
BRUKER
NAME UN
EXPNO 426
PROCNO 1
Date_ 20250513
INSTRUM spect
PROBHD 5 mm PABBO BB-
PULPROG zg30
TD 65536
SOLVENT CDCl3
NS 24
DS 0
SWH 8012.820 Hz
FIDRES 0.122266 Hz
AQ 4.0894966 sec
RG 406
DW 62.400 usec
DE 6.50 usec
TE 293.2 K
D1 6.00000000 sec
TD0 1
======== CHANNEL f1 ========
NUC1 1H
P1 14.00 usec
PL1 -2.00 dB
PL1W 11.86359406 W
SFO1 400.2236020 MHz
SI 32768
SF 400.2200000 MHz
WDW EM
SSB 0
LB 0.30 Hz
GB 0
PC 1.00
0.98 1.01 1.00 2.00 2.00 1.99 2.03 4.00 3.99 1.02
12 11 10 9 8 7 6 5 4 3 2 1 0
f1 (ppm)

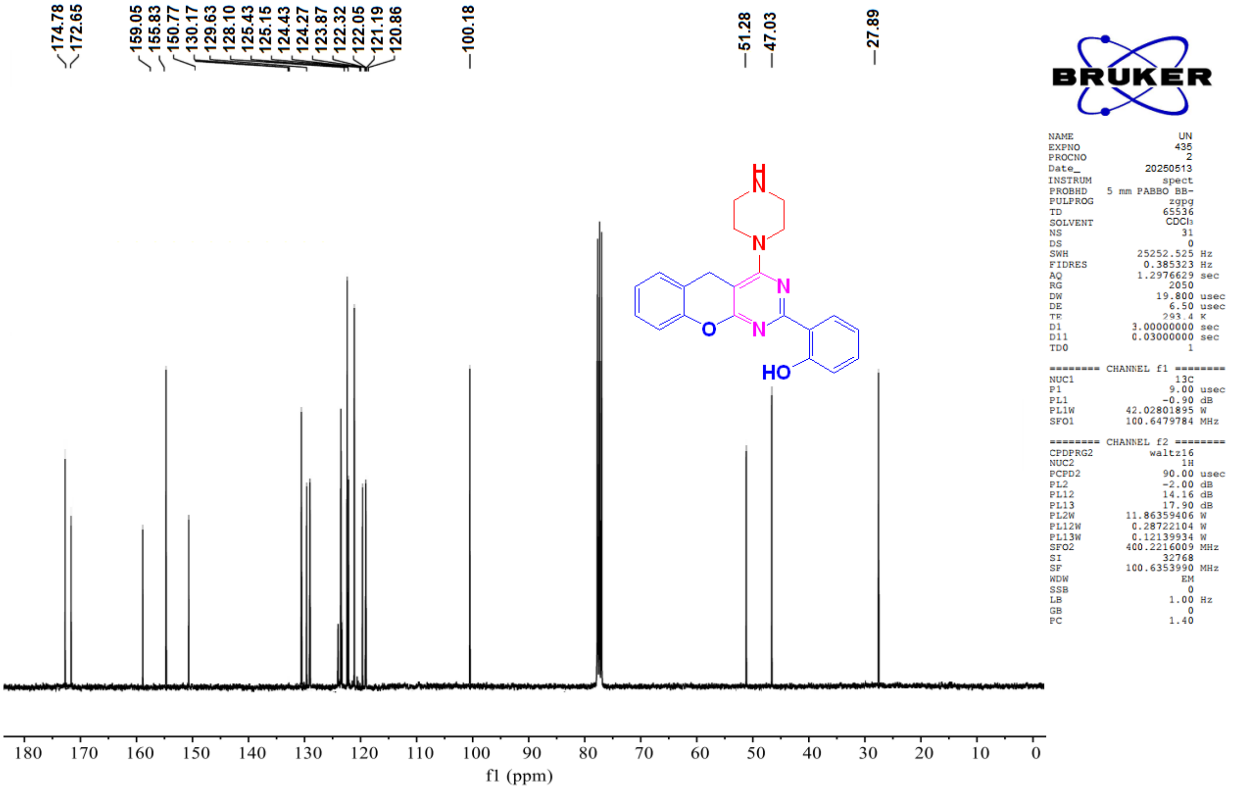
174.78 172.65 159.05 155.83 150.77 130.17 129.63 128.10 125.43 125.15 124.43 124.27 123.87 122.32 122.05 121.19 120.86 100.18 51.28 47.03 27.89
H
N
N
N
N
O
HO
BRUKER
NAME UN
EXPNO 435
PROCNO 2
Date_ 20250513
INSTRUM spect
PROBHD 5 mm PABBO BB-
PULPROG zgpg
TD 65536
SOLVENT CDCl3
NS 31
DS 0
SWH 25252.525 Hz
FIDRES 0.385323 Hz
AQ 1.2976629 sec
RG 2050
DW 19.800 usec
DE 6.50 usec
D1 3.00000000 sec
D11 0.03000000 sec
TD0 1
======== CHANNEL f1 ========
NUC1 13C
P1 9.00 usec
PL1 -0.90 dB
PL1W 42.02801895 W
SFO1 100.6479784 MHz
======== CHANNEL f2 ========
CPDPRG2 waltz16
NUC2 1H
PCPD2 90.00 usec
PL2 -2.00 dB
PL12 14.16 dB
PL13 17.90 dB
PL2W 11.86359406 W
PL12W 0.28722104 W
PL13W 0.12139934 W
SFO2 400.2216009 MHz
SI 32768
SF 100.6353990 MHz
WDW EM
SSB 0
LB 1.00 Hz
GB 0
PC 1.40
180 170 160 150 140 130 120 110 100 90 80 70 60 50 40 30 20 10 0
f1 (ppm)

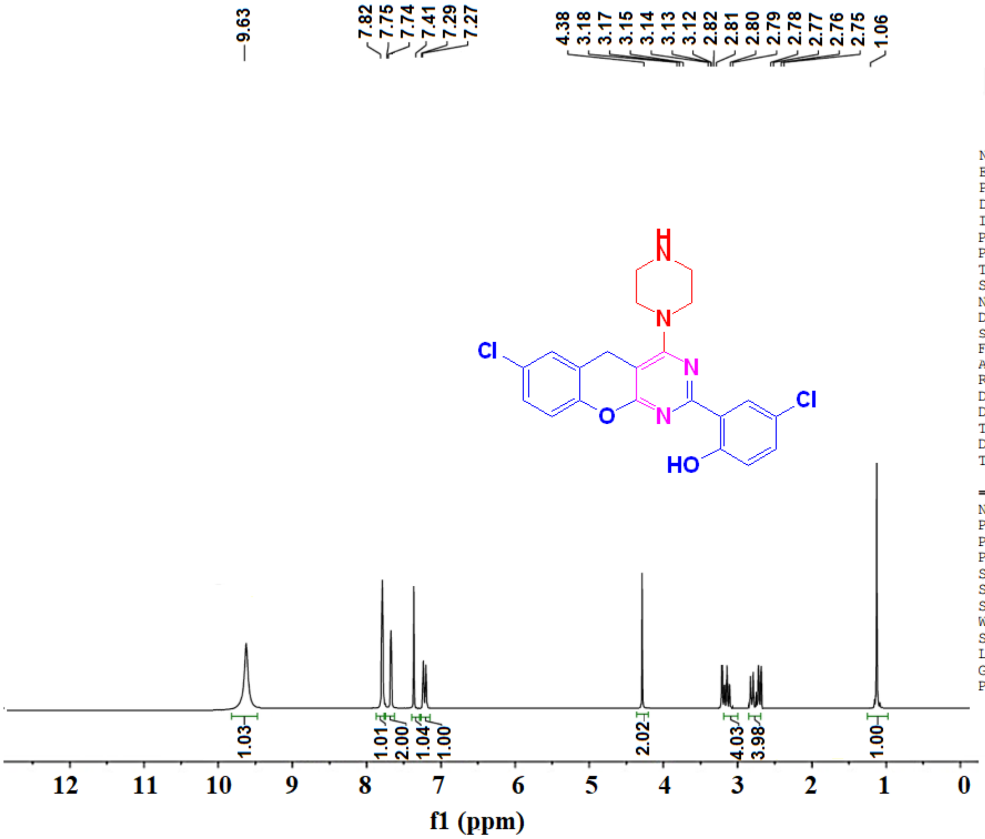

NAME	UN
EXPNC	426
PROCNO	1
Date_	20250513
INSTRUM	spect
PROBHD	5 mm PABBO BB-
PULPROG	zg30
TD	65536
SOLVENT	$CDCl_3$
NS	24
DS	0
SWH	8012.820 Hz
FIDRES	0.122266 Hz
AQ	4.0894966 sec
RG	406
DW	62.400 usec
DE	6.50 usec
TE	293.2 K
D1	6.00000000 sec
TD0	1

======== CHANNEL f1 ========

NUC1	1H
P1	14.00 usec
PL1	-2.00 dB
PL1W	11.86359406 W
SFO1	400.2236020 MHz
SI	32768
SF	400.2200000 MHz
WDW	EM
SSB	0
LB	0.30 Hz
GB	0
PC	1.00

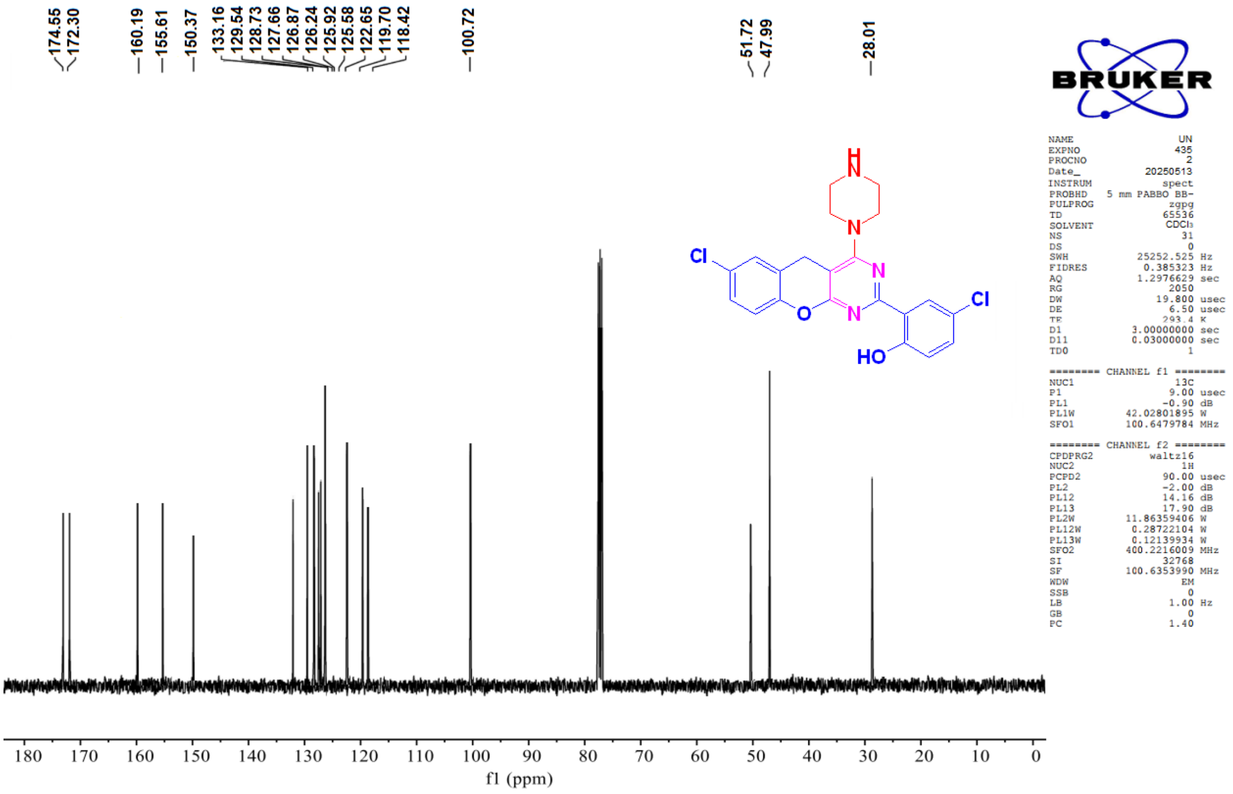

174.55
172.30
160.19
155.61
150.37
133.16
129.54
128.73
127.66
126.87
126.24
125.92
125.58
122.65
119.70
118.42
100.72
51.72
47.99
28.01
Cl
Cl
HO
O
N
N
N
H
BRUKER
NAME UN
EXPNO 435
PROCNO 2
Date_ 20250513
INSTRUM spect
PROBHD 5 mm PABBO BB-
PULPROG zgpg
TD 65536
SOLVENT CDCl3
NS 31
DS 0
SWH 25252.525 Hz
FIDRES 0.385323 Hz
AQ 1.2976629 sec
RG 2050
DW 19.800 usec
DE 6.50 usec
TE 293.4 K
D1 3.00000000 sec
D11 0.03000000 sec
TD0 1
======== CHANNEL f1 ========
NUC1 13C
P1 9.00 usec
PL1 -0.90 dB
PL1W 42.02801895 W
SFO1 100.6479784 MHz
======== CHANNEL f2 ========
CPDPRG2 waltz16
NUC2 1H
PCPD2 90.00 usec
PL2 -2.00 dB
PL12 14.16 dB
PL13 17.90 dB
PL2W 11.86359406 W
PL12W 0.28722104 W
PL13W 0.12139934 W
SFO2 400.2216009 MHz
SI 32768
SF 100.6353990 MHz
WDW EM
SSB 0
LB 1.00 Hz
GB 0
PC 1.40
180 170 160 150 140 130 120 110 100 90 80 70 60 50 40 30 20 10 0
f1 (ppm)

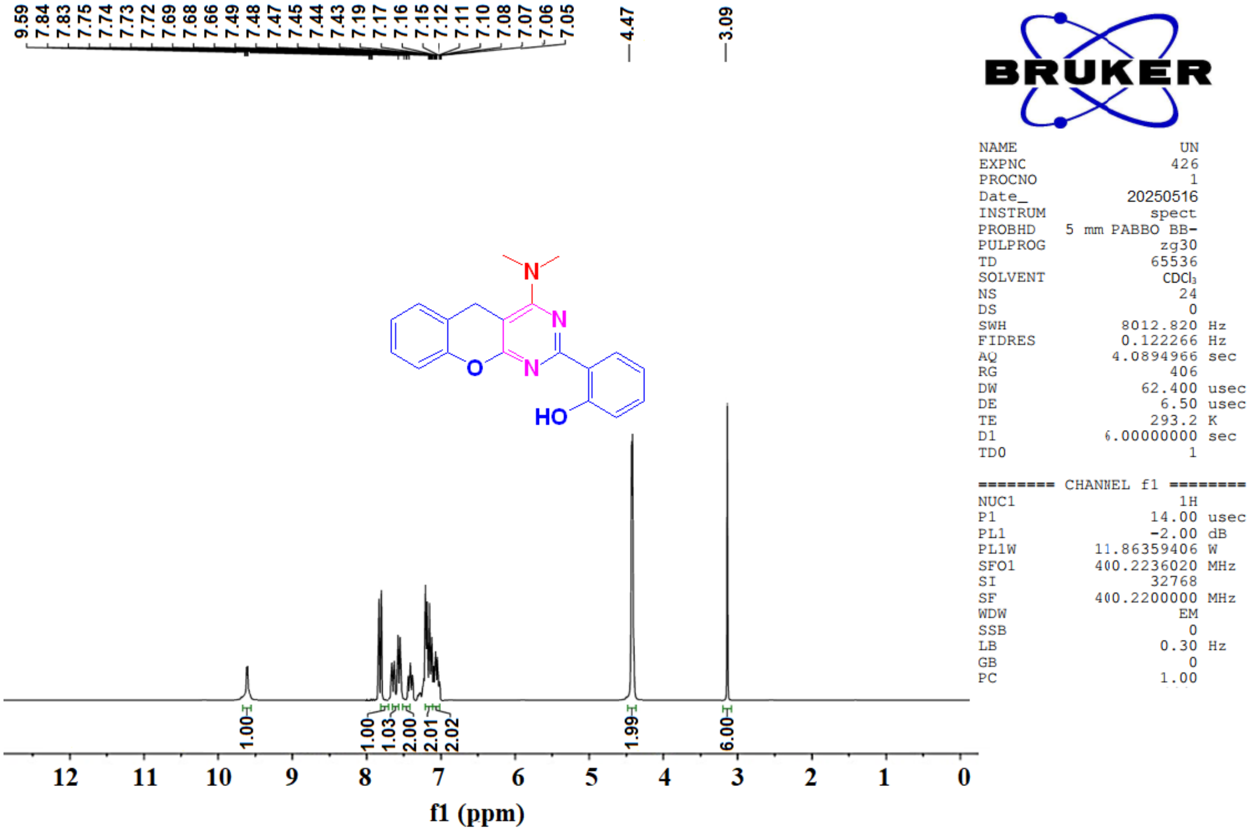

9.59
7.84
7.83
7.75
7.74
7.73
7.72
7.69
7.68
7.66
7.49
7.48
7.47
7.45
7.44
7.43
7.19
7.17
7.16
7.15
7.12
7.11
7.10
7.08
7.07
7.06
7.05
4.47
3.09
N
N
N
O
HO
BRUKER
NAME UN
EXPNO 426
PROCNO 1
Date_ 20250516
INSTRUM spect
PROBHD 5 mm PABBO BB-
PULPROG zg30
TD 65536
SOLVENT CDCl3
NS 24
DS 0
SWH 8012.820 Hz
FIDRES 0.122266 Hz
AQ 4.0894966 sec
RG 406
DW 62.400 usec
DE 6.50 usec
TE 293.2 K
D1 6.00000000 sec
TD0 1
======== CHANNEL f1 ========
NUC1 1H
P1 14.00 usec
PL1 -2.00 dB
PL1W 11.86359406 W
SFO1 400.2236020 MHz
SI 32768
SF 400.2200000 MHz
WDW EM
SSB 0
LB 0.30 Hz
GB 0
PC 1.00
1.00
1.00
1.03
2.00
2.01
2.02
1.99
6.00
12 11 10 9 8 7 6 5 4 3 2 1 0
f1 (ppm)

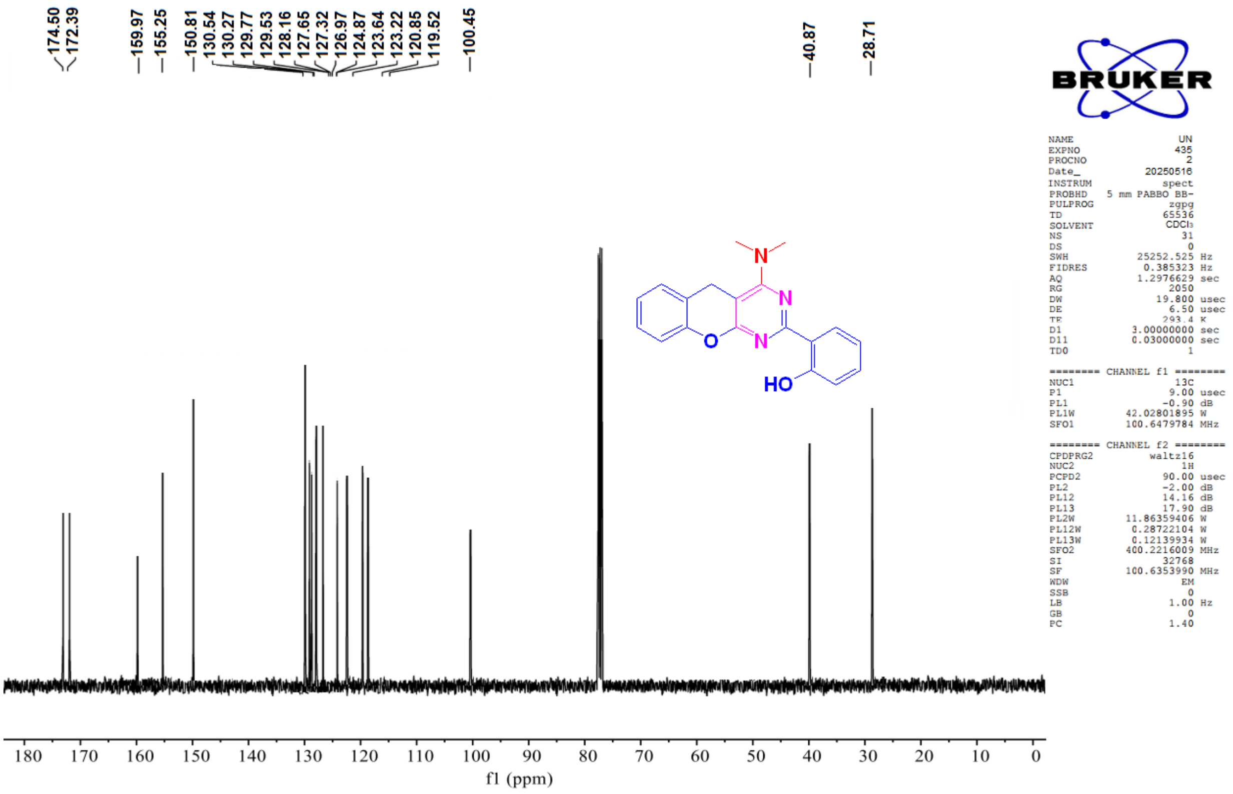
174.50
172.39
159.97
155.25
150.81
130.54
130.27
129.77
129.53
128.16
127.65
127.32
126.97
124.87
123.64
123.22
120.85
119.52
100.45
40.87
28.71
N
N
N
O
HO
BRUKER
NAME UN
EXPNO 435
PROCNO 2
Date_ 20250516
INSTRUM spect
PROBHD 5 mm PABBO BB-
PULPROG zgpg
TD 65536
SOLVENT CDCl3
NS 31
DS 0
SWH 25252.525 Hz
FIDRES 0.385323 Hz
AQ 1.2976629 sec
RG 2050
DW 19.800 usec
DE 6.50 usec
D1 3.00000000 sec
D11 0.03000000 sec
TD0 1
======== CHANNEL f1 ========
NUC1 13C
P1 9.00 usec
PL1 -0.90 dB
PL1W 42.02801895 W
SFO1 100.6479784 MHz
======== CHANNEL f2 ========
CPDPRG2 waltz16
NUC2 1H
PCPD2 90.00 usec
PL2 -2.00 dB
PL12 14.16 dB
PL13 17.90 dB
PL2W 11.86359406 W
PL12W 0.28722104 W
PL13W 0.12139934 W
SFO2 400.2216009 MHz
SI 32768
SF 100.6353990 MHz
WDW EM
SSB 0
LB 1.00 Hz
GB 0
PC 1.40
180 170 160 150 140 130 120 110 100 90 80 70 60 50 40 30 20 10 0
f1 (ppm)

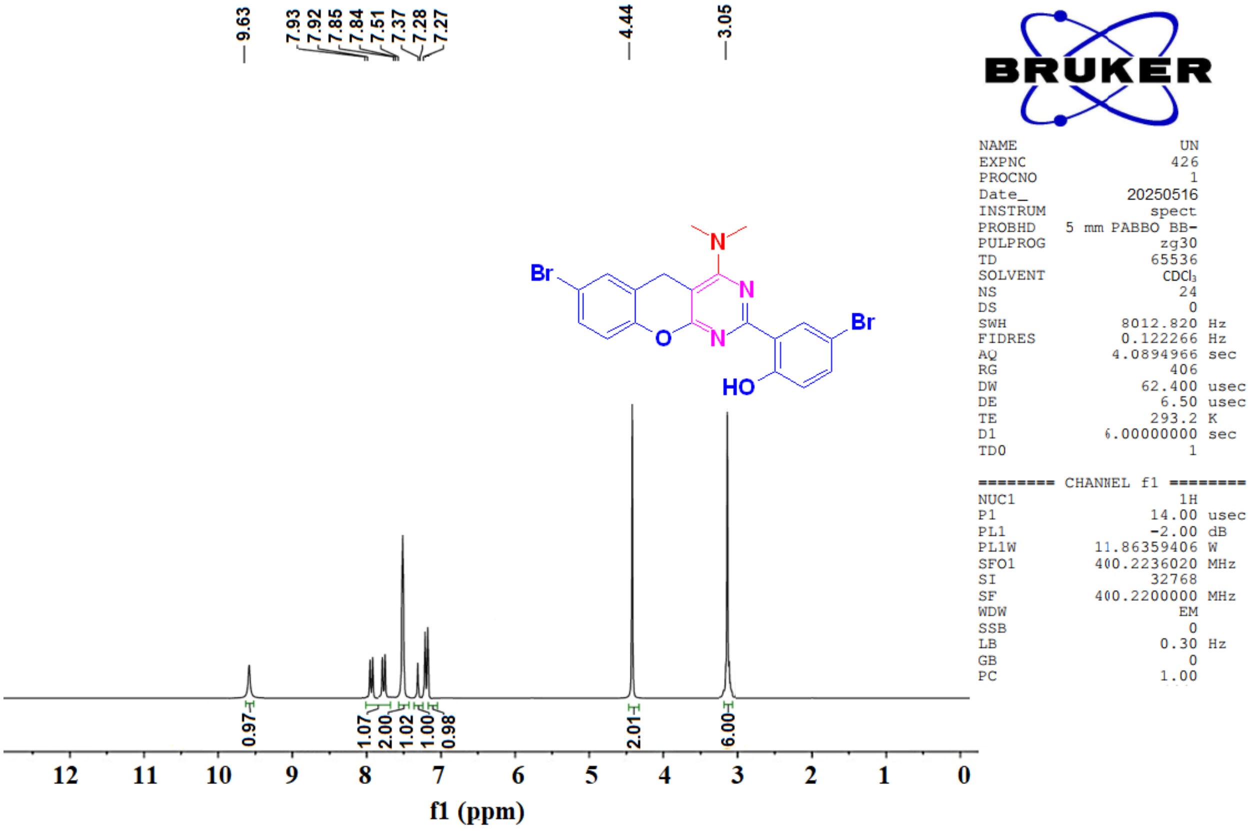
9.63
7.93
7.92
7.85
7.84
7.51
7.37
7.28
7.27
4.44
3.05
BRUKER
NAME UN
EXPNC 426
PROCNO 1
Date_ 20250516
INSTRUM spect
PROBHD 5 mm PABBO BB-
PULPROG zg30
TD 65536
SOLVENT CDCl3
NS 24
DS 0
SWH 8012.820 Hz
FIDRES 0.122266 Hz
AQ 4.0894966 sec
RG 406
DW 62.400 usec
DE 6.50 usec
TE 293.2 K
D1 6.00000000 sec
TD0 1
======== CHANNEL f1 ========
NUC1 1H
P1 14.00 usec
PL1 -2.00 dB
PL1W 11.86359406 W
SFO1 400.2236020 MHz
SI 32768
SF 400.2200000 MHz
WDW EM
SSB 0
LB 0.30 Hz
GB 0
PC 1.00
Br
Br
N
N
N
O
HO
0.97
1.07
2.00
1.02
1.00
0.98
2.01
6.00
12 11 10 9 8 7 6 5 4 3 2 1 0
f1 (ppm)

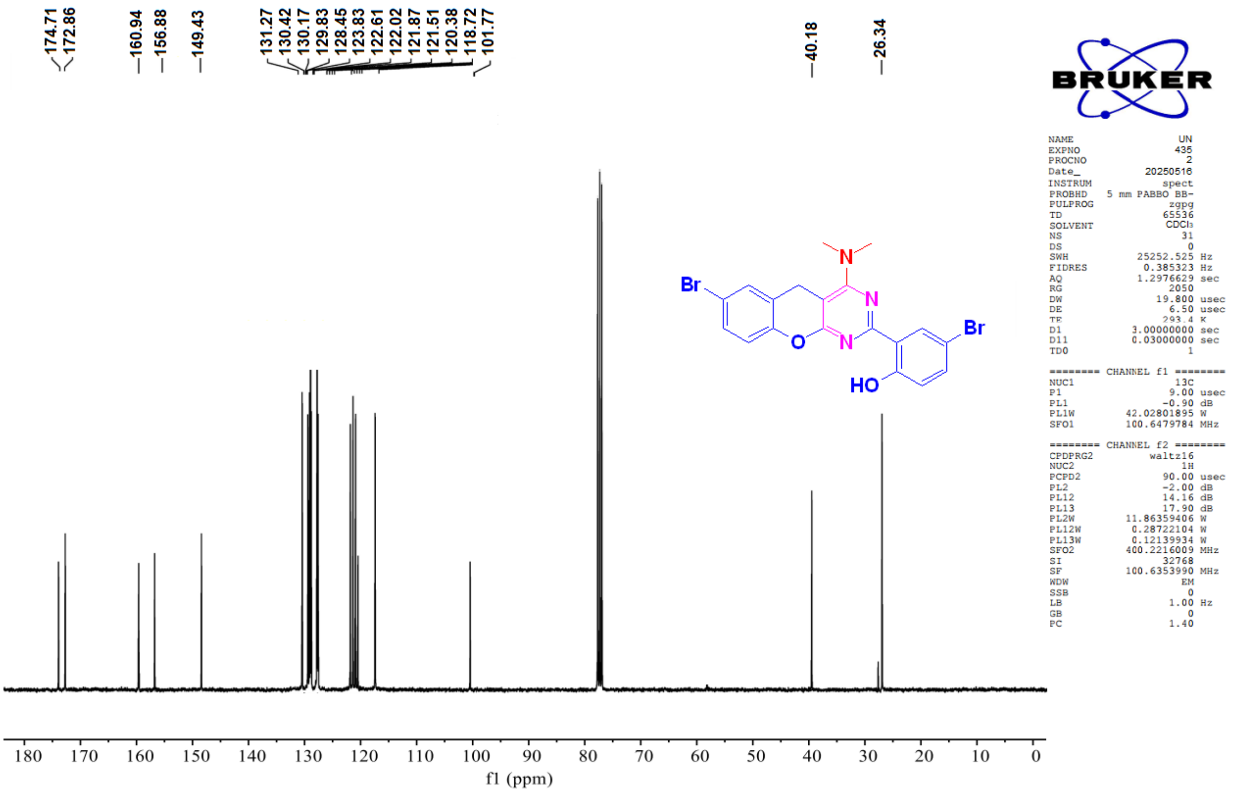
174.71
172.86
160.94
156.88
149.43
131.27
130.42
130.17
129.83
128.45
123.83
122.61
122.02
121.87
121.51
120.38
118.72
101.77
40.18
26.34
Br
N
N
N
O
Br
HO
NAME UN
EXPNO 435
PROCNO 2
Date_ 20250516
INSTRUM spect
PROBHD 5 mm PABBO BB-
PULPROG zgpg
TD 65536
SOLVENT CDCl3
NS 31
DS 0
SWH 25252.525 Hz
FIDRES 0.385323 Hz
AQ 1.2976629 sec
RG 2050
DW 19.800 usec
DE 6.50 usec
D1 3.00000000 sec
D11 0.03000000 sec
TD0 1
======== CHANNEL f1 ========
NUC1 13C
P1 9.00 usec
PL1 -0.90 dB
PL1W 42.02801895 W
SFO1 100.6479784 MHz
======== CHANNEL f2 ========
CPDPRG2 waltz16
NUC2 1H
PCPD2 90.00 usec
PL2 -2.00 dB
PL12 14.16 dB
PL13 17.90 dB
PL2W 11.86359406 W
PL12W 0.28722104 W
PL13W 0.12139934 W
SFO2 400.2216009 MHz
SI 32768
SF 100.6353990 MHz
WDW EM
SSB 0
LB 1.00 Hz
GB 0
PC 1.40
180 170 160 150 140 130 120 110 100 90 80 70 60 50 40 30 20 10 0
f1 (ppm)

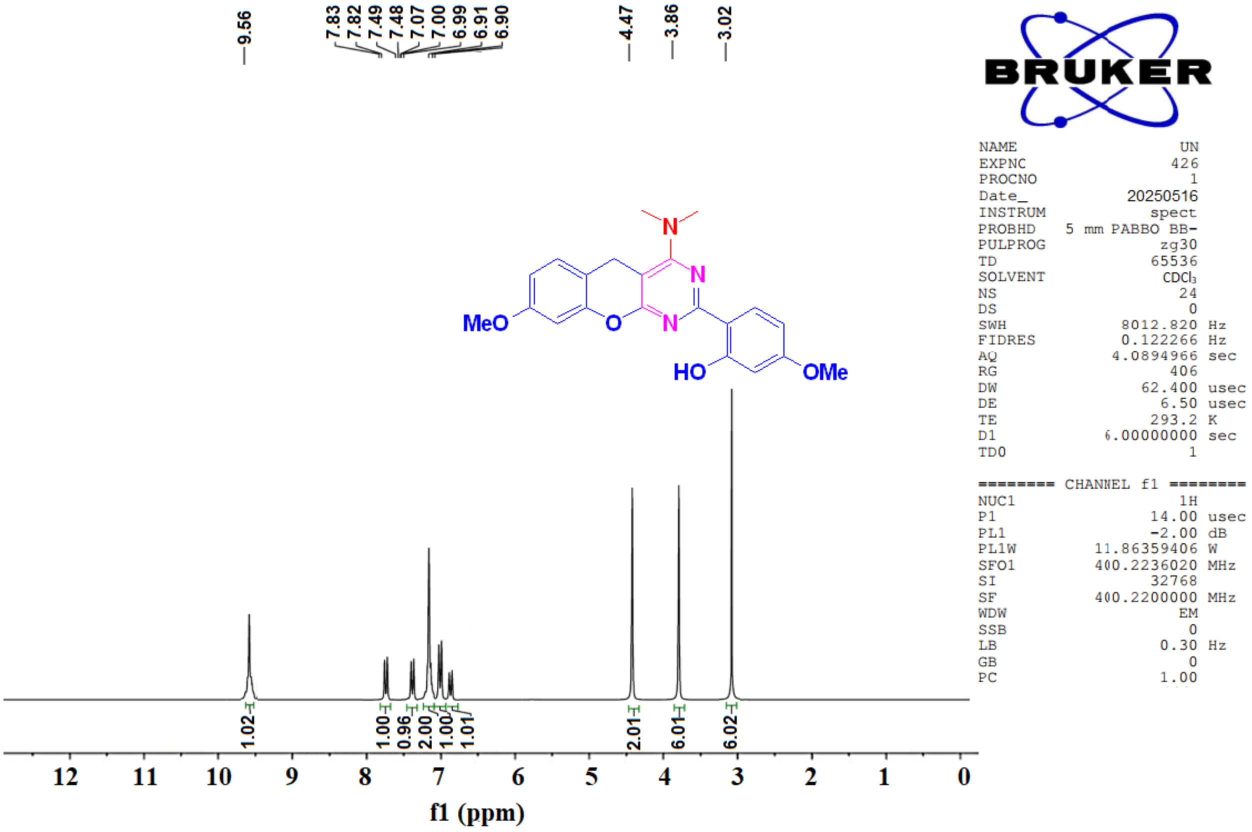
9.56
7.83
7.82
7.49
7.48
7.07
7.00
6.99
6.91
6.90
4.47
3.86
3.02
N
N
MeO
O
N
HO
OMe
NAME UN
EXPNC 426
PROCNO 1
Date_ 20250516
INSTRUM spect
PROBHD 5 mm PABBO BB-
PULPROG zg30
TD 65536
SOLVENT CDCl3
NS 24
DS 0
SWH 8012.820 Hz
FIDRES 0.122266 Hz
AQ 4.0894966 sec
RG 406
DW 62.400 usec
DE 6.50 usec
TE 293.2 K
D1 6.00000000 sec
TD0 1
======== CHANNEL f1 ========
NUC1 1H
P1 14.00 usec
PL1 -2.00 dB
PL1W 11.86359406 W
SFO1 400.2236020 MHz
SI 32768
SF 400.2200000 MHz
WDW EM
SSB 0
LB 0.30 Hz
GB 0
PC 1.00
1.02
1.00
0.96
2.00
1.00
1.01
2.01
6.01
6.02
12 11 10 9 8 7 6 5 4 3 2 1 0
f1 (ppm)

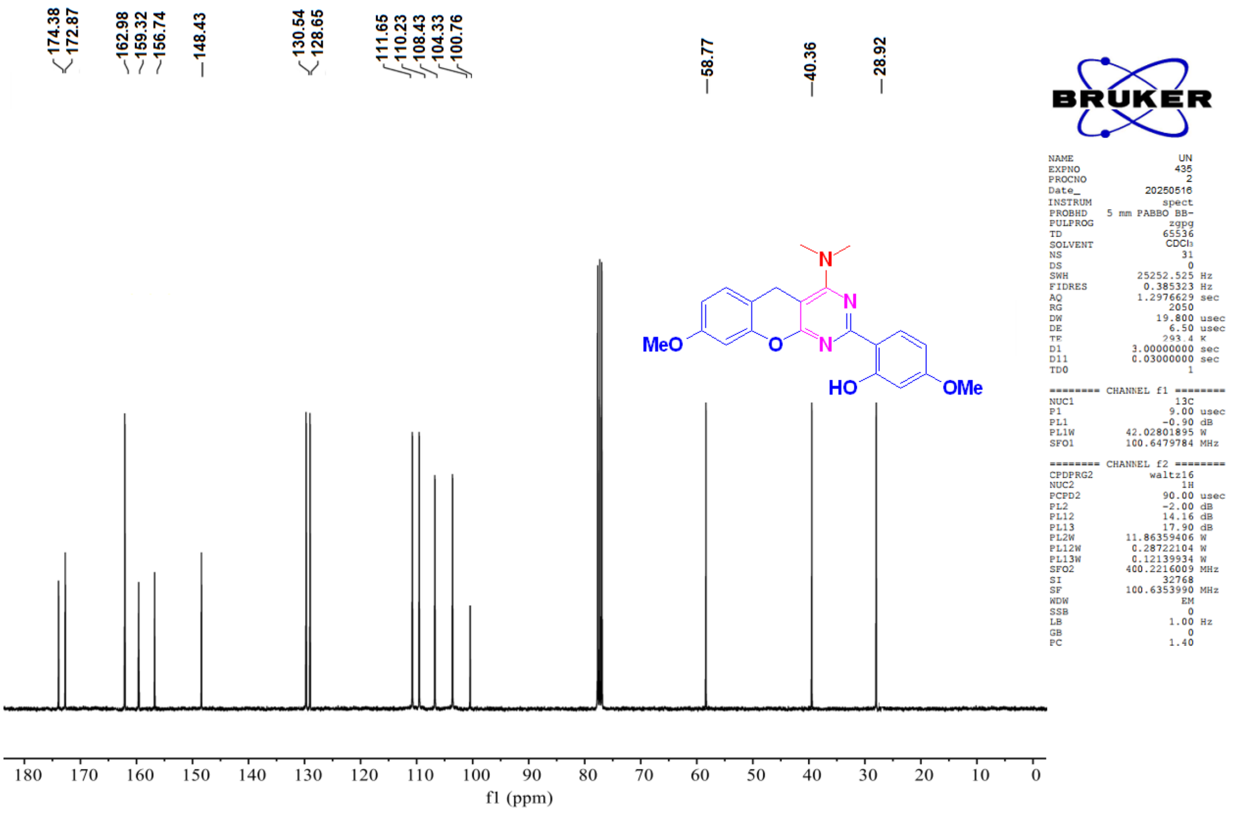
174.38
172.87
162.98
159.32
156.74
148.43
130.54
128.65
111.65
110.23
108.43
104.33
100.76
58.77
40.36
28.92
N
N
N
O
MeO
HO
OMe
BRUKER
NAME UN
EXPNO 435
PROCNO 2
Date_ 20250516
INSTRUM spect
PROBHD 5 mm PABBO BB-
PULPROG zgpg
TD 65536
SOLVENT CDCl3
NS 31
DS 0
SWH 25252.525 Hz
FIDRES 0.385323 Hz
AQ 1.2976629 sec
RG 2050
DW 19.800 usec
DE 6.50 usec
D1 3.00000000 sec
D11 0.03000000 sec
TD0 1
======== CHANNEL f1 ========
NUC1 13C
P1 9.00 usec
PL1 -0.90 dB
PL1W 42.02801895 W
SFO1 100.6479784 MHz
======== CHANNEL f2 ========
CPDPRG2 waltz16
NUC2 1H
PCPD2 90.00 usec
PL2 -2.00 dB
PL12 14.16 dB
PL13 17.90 dB
PL2W 11.86359406 W
PL12W 0.28722104 W
PL13W 0.12139934 W
SFO2 400.2216009 MHz
SI 32768
SF 100.6353990 MHz
WDW EM
SSB 0
LB 1.00 Hz
GB 0
PC 1.40
180 170 160 150 140 130 120 110 100 90 80 70 60 50 40 30 20 10 0
f1 (ppm)

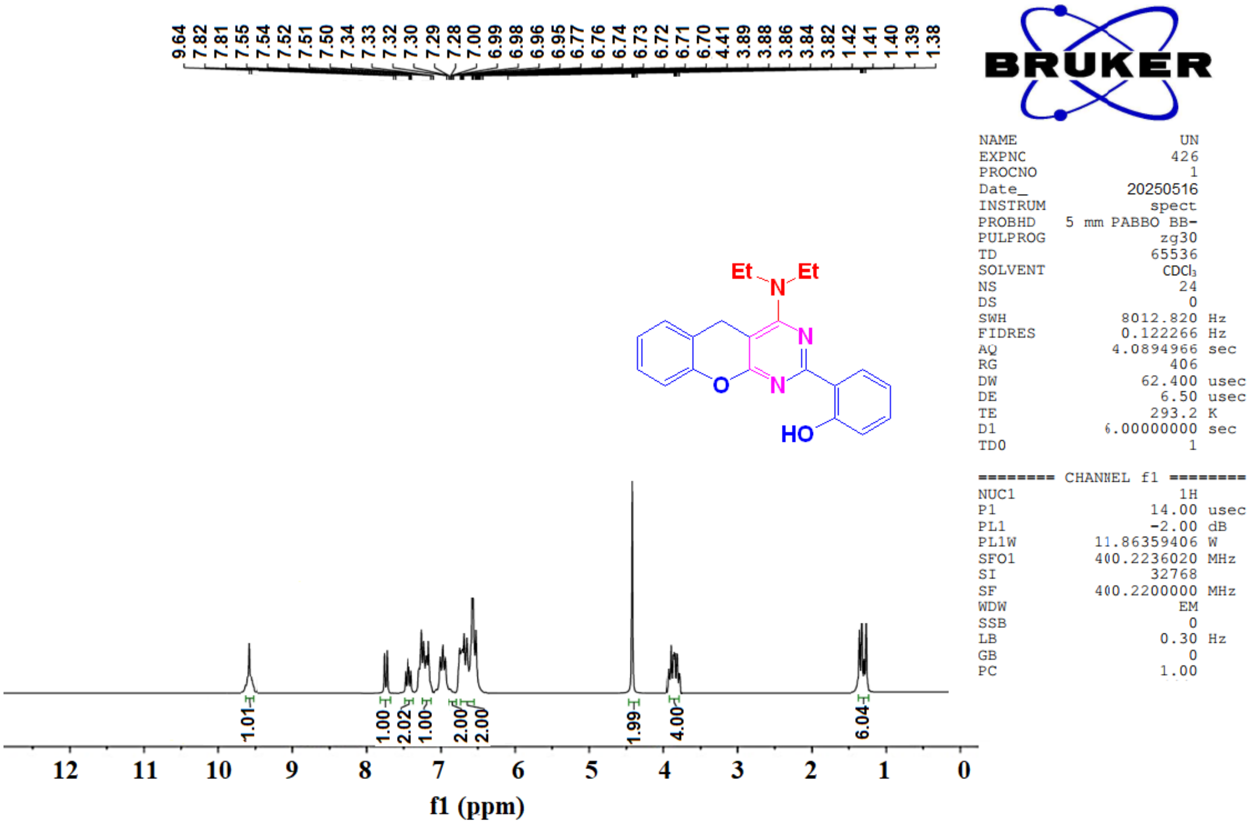
9.64 7.82 7.81 7.55 7.54 7.52 7.51 7.50 7.34 7.33 7.32 7.30 7.29 7.28 7.00 6.99 6.98 6.96 6.95 6.77 6.76 6.74 6.73 6.72 6.71 6.70 4.41 3.89 3.88 3.86 3.84 3.82 1.42 1.41 1.40 1.39 1.38
Et
N
Et
N
N
O
HO
BRUKER
NAME UN
EXPNC 426
PROCNO 1
Date_ 20250516
INSTRUM spect
PROBHD 5 mm PABBO BB-
PULPROG zg30
TD 65536
SOLVENT CDCl3
NS 24
DS 0
SWH 8012.820 Hz
FIDRES 0.122266 Hz
AQ 4.0894966 sec
RG 406
DW 62.400 usec
DE 6.50 usec
TE 293.2 K
D1 6.00000000 sec
TD0 1
======== CHANNEL f1 ========
NUC1 1H
P1 14.00 usec
PL1 -2.00 dB
PL1W 11.86359406 W
SFO1 400.2236020 MHz
SI 32768
SF 400.2200000 MHz
WDW EM
SSB 0
LB 0.30 Hz
GB 0
PC 1.00
1.01
1.00
2.02
1.00
2.00
2.00
1.99
4.00
6.04
12 11 10 9 8 7 6 5 4 3 2 1 0
f1 (ppm)

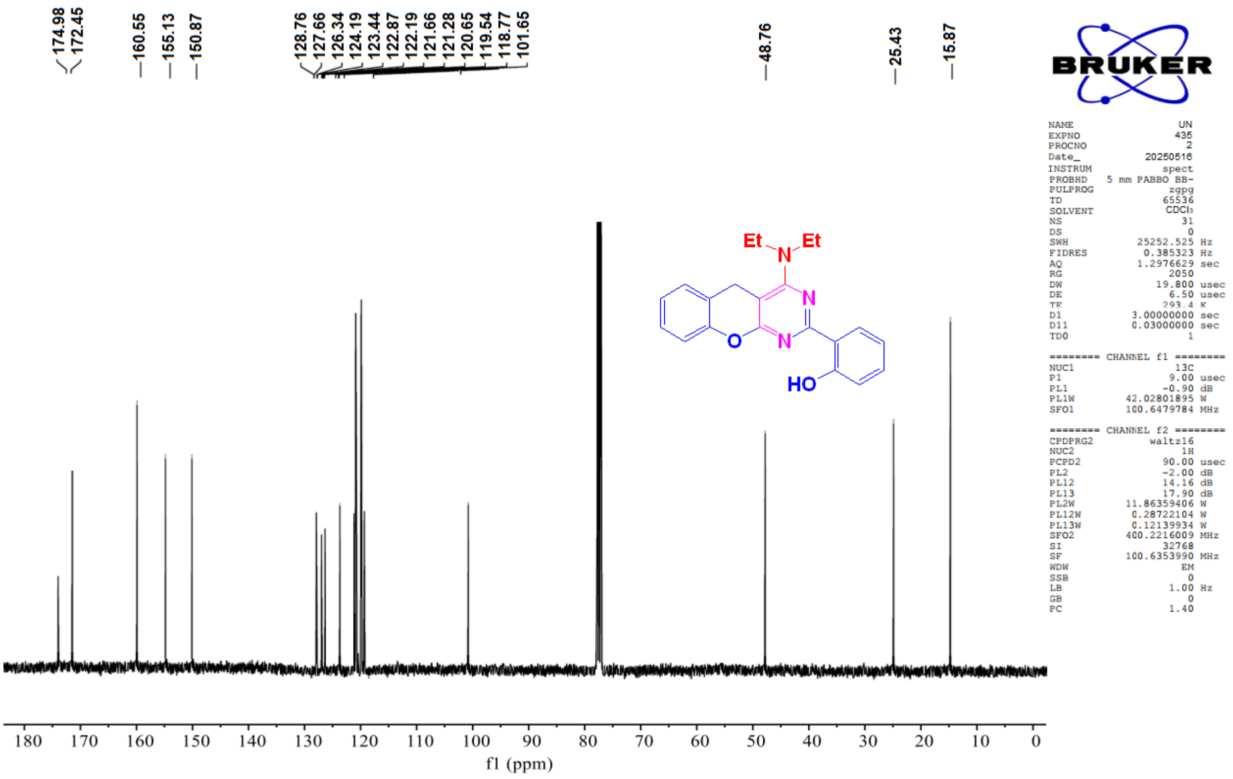
174.98
172.45
160.55
155.13
150.87
128.76
127.66
126.34
124.19
123.44
122.87
122.19
121.66
121.28
120.65
119.54
118.77
101.65
48.76
25.43
15.87
Et
N
Et
N
N
O
HO
BRUKER
NAME UN
EXPNO 435
PROCNO 2
Date_ 20250516
INSTRUM spect
PROBHD 5 mm PABBO BB-
PULPROG zgpg
TD 65536
SOLVENT CDCl3
NS 31
DS 0
SWH 25252.525 Hz
FIDRES 0.385323 Hz
AQ 1.2976629 sec
RG 2050
DW 19.800 usec
DE 6.50 usec
TE 293.4 K
D1 3.00000000 sec
D11 0.03000000 sec
TD0 1
======== CHANNEL f1 ========
NUC1 13C
P1 9.00 usec
PL1 -0.90 dB
PL1W 42.02801895 W
SFO1 100.6479784 MHz
======== CHANNEL f2 ========
CPDPRG2 waltz16
NUC2 1H
PCPD2 90.00 usec
PL2 -2.00 dB
PL12 14.16 dB
PL13 17.90 dB
PL2W 11.86359406 W
PL12W 0.28722104 W
PL13W 0.12139934 W
SFO2 400.2216009 MHz
SI 32768
SF 100.6353990 MHz
WDW EM
SSB 0
LB 1.00 Hz
GB 0
PC 1.40
180 170 160 150 140 130 120 110 100 90 80 70 60 50 40 30 20 10 0
f1 (ppm)

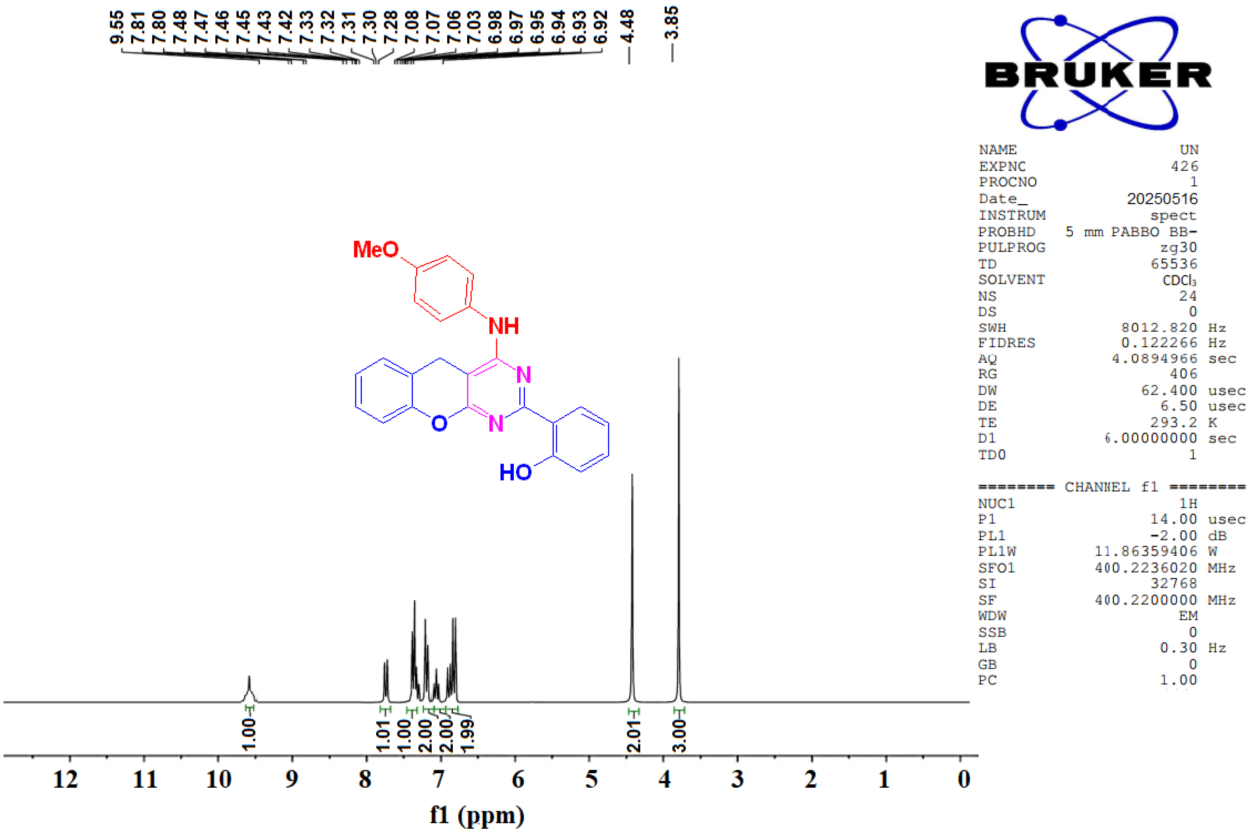
9.55
7.81
7.80
7.48
7.47
7.46
7.45
7.43
7.42
7.33
7.32
7.31
7.30
7.28
7.08
7.07
7.06
7.03
6.98
6.97
6.95
6.94
6.93
6.92
4.48
3.85
MeO
NH
N
O
N
HO
BRUKER
NAME UN
EXPNO 426
PROCNO 1
Date_ 20250516
INSTRUM spect
PROBHD 5 mm PABBO BB-
PULPROG zg30
TD 65536
SOLVENT CDCl3
NS 24
DS 0
SWH 8012.820 Hz
FIDRES 0.122266 Hz
AQ 4.0894966 sec
RG 406
DW 62.400 usec
DE 6.50 usec
TE 293.2 K
D1 6.00000000 sec
TD0 1
======== CHANNEL f1 ========
NUC1 1H
P1 14.00 usec
PL1 -2.00 dB
PL1W 11.86359406 W
SFO1 400.2236020 MHz
SI 32768
SF 400.2200000 MHz
WDW EM
SSB 0
LB 0.30 Hz
GB 0
PC 1.00
1.00
1.01
1.00
2.00
2.00
1.99
2.01
3.00
12 11 10 9 8 7 6 5 4 3 2 1 0
f1 (ppm)

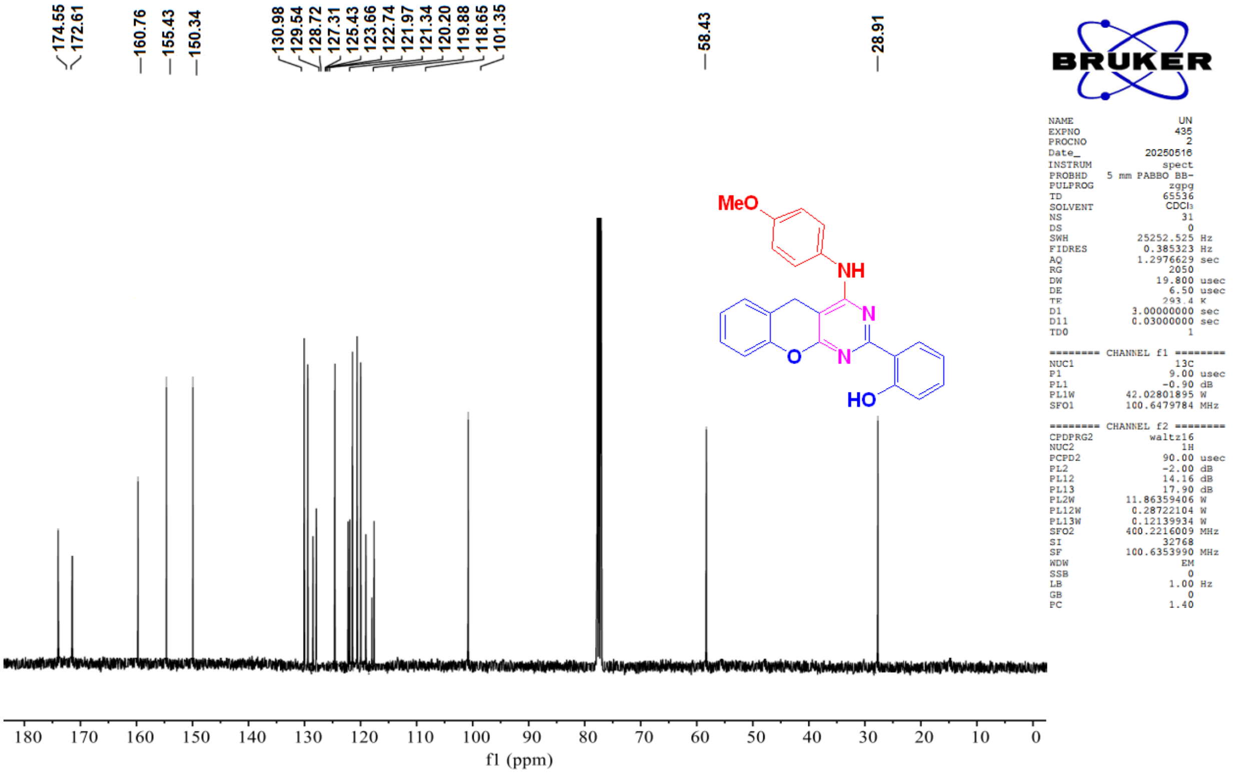
174.55
172.61
160.76
155.43
150.34
130.98
129.54
128.72
127.31
125.43
123.66
122.74
121.97
121.34
120.20
119.88
118.65
101.35
58.43
28.91
BRUKER
MeO
NH
N
O
N
HO
NAME UN
EXPNO 435
PROCNO 2
Date_ 20250516
INSTRUM spect
PROBHD 5 mm PABBO BB-
PULPROG zgpg
TD 65536
SOLVENT CDCl3
NS 31
DS 0
SWH 25252.525 Hz
FIDRES 0.385323 Hz
AQ 1.2976629 sec
RG 2050
DW 19.800 usec
DE 6.50 usec
D1 3.00000000 sec
D11 0.03000000 sec
TD0 1
======== CHANNEL f1 ========
NUC1 13C
P1 9.00 usec
PL1 -0.90 dB
PL1W 42.02801895 W
SFO1 100.6479784 MHz
======== CHANNEL f2 ========
CPDPRG2 waltz16
NUC2 1H
PCPD2 90.00 usec
PL2 -2.00 dB
PL12 14.16 dB
PL13 17.90 dB
PL2W 11.86359406 W
PL12W 0.28722104 W
PL13W 0.12139934 W
SFO2 400.2216009 MHz
SI 32768
SF 100.6353990 MHz
WDW EM
SSB 0
LB 1.00 Hz
GB 0
PC 1.40
180 170 160 150 140 130 120 110 100 90 80 70 60 50 40 30 20 10 0
f1 (ppm)
